# Trends and cross-country inequality in the global burden of nutritional deficiencies in children, with projections to 2035: results from the Global Burden of Disease study 2021

**DOI:** 10.3389/fnut.2025.1615593

**Published:** 2025-07-29

**Authors:** Shihao Zhuang, Meijiang Ruan, Qin Chen, Qiaomei Wang, Ting Chen, Hao Wang, Shanshan Liu, Qiudan Chen, Chengchen Zhang, Li Hong

**Affiliations:** ^1^College of Clinical Medicine for Obstetrics & Gynecology and Pediatrics, Fujian Medical University, Fuzhou, China; ^2^Fujian Children's Hospital, Fuzhou, China; ^3^Fujian Branch of Shanghai Children's Medical Center Affiliated to Shanghai Jiaotong University School of Medicine, Fuzhou, China; ^4^Fujian Maternity and Child Health Hospital, College of Clinical Medicine for Obstetrics & Gynecology and Pediatrics, Fujian Medical University, Fuzhou, China; ^5^Fujian Maternity and Child Health Hospital, Affiliated Hospital of Fujian Medical University, Fuzhou, China; ^6^The Second Affiliated Hospital of Fujian University of Traditional Chinese Medicine (The Second People's Hospital of Fujian Province), Fuzhou, China; ^7^Xiamen Hong'ai Hospital, Xiamen, China; ^8^School of Medicine, Shanghai University, Shanghai, China; ^9^School of Public Health, Shandong University, Jinan, China; ^10^Department of Clinical Laboratory, Fuzhou Pulmonary Hospital of Fujian, Fuzhou, China; ^11^Department of Clinical Laboratory, Central Laboratory, Jing'an District Central Hospital of Shanghai, Fudan University, Shanghai, China; ^12^Shanghai Children's Medical Center, School of Medicine, Shanghai Jiao Tong University, Shanghai, China; ^13^Department of Clinical Nutrition, Shanghai Children's Medical Center, School of Medicine, Shanghai Jiao Tong University, Shanghai, China; ^14^Fujian Children's Hospital (Fujian Branch of Shanghai Children's Medical Center), College of Clinical Medicine for Obstetrics & Gynecology and Pediatrics, Fujian Medical University, Fuzhou, China

**Keywords:** nutritional deficiencies, children's health, Global Burden of Disease, Frontier analysis, Bayesian age-period-cohort model

## Abstract

**Background:**

Nutritional deficiencies in children are a significant global health concern, contributing to considerable morbidity and mortality. This study evaluates the burden of children's nutritional deficiencies from 1990 to 2021, focusing on key indicators and exploring regional disparities and the role of socio-economic factors.

**Methods:**

Data from the Global Burden of Disease 2021 study were analyzed for children's nutritional deficiencies across 204 countries and territories. Age-standardized rates (ASRs) for prevalence (ASPR), incidence (ASIR), DALYs (ASDR), and mortality (ASMR) were calculated. Trends were assessed using estimated annual percentage changes (EAPC), and decomposition analysis was conducted to evaluate the drivers of changes in the burden of nutritional deficiencies. Projections to 2035 were made using the Bayesian age-period-cohort model and Health inequality was assessed to analyze transnational health inequality.

**Results:**

From 1990 to 2021, the global incidence of children's nutritional deficiencies decreased by 51.51%, with the age-standardized incidence rate (ASIR) dropping to 11,741.75 per 100,000. Global prevalence decreased by 18.44%, and DALYs dropped by 59.57%. Deaths due to nutritional deficiencies reduced by 80.56%. Despite these global improvements, significant regional disparities persisted. Sub-Saharan Africa reported the highest ASIR and ASPR, while high-SDI regions such as North America and Australasia exhibited significantly lower rates. Projections for 2035 indicate continued declines in global incidence, prevalence, DALYs, and mortality, with age-standardized rates expected to decrease annually. By 2035, the ASIR, ASPR, ASDR, and ASMR are projected to reach 7,469.67, 26,386.33, 306.95, and 1.73 per 100,000, respectively. However, disparities in age-standardized rates between high- and low-SDI regions are expected to persist. Health inequality analysis revealed a significant negative correlation between SDI and the burden of nutritional deficiencies, with countries in lower SDI categories facing disproportionately high burdens compared to those with higher SDI.

**Conclusions:**

Although the global burden of children's nutritional deficiencies is projected to continue declining in both age-standardized rates and total cases, the burden remains disproportionately high in low-SDI regions. These regions face greater challenges in addressing nutritional deficiencies, and targeted interventions aimed at reducing these inequalities are essential. Addressing the significant disparities between low- and high-SDI countries will be crucial for further reducing the global burden of children's nutritional deficiencies.

## Introduction

Nutritional deficiencies are a significant global public health issue (1), particularly among children. Malnutrition directly impacts children's growth and development, as well as immune system function, leading to increased morbidity, mortality, and disability-adjusted life years (DALYs) (2). Diseases resulting from malnutrition include, but are not limited to, anemia, night blindness, immune dysfunction, and developmental delays. These conditions significantly reduce the quality of life for children, affect their long-term health development, and, in some cases, may even lead to early death (3).

Globally, especially in low- and middle-income countries, deficiencies in key micronutrients such as vitamin A, iron, and iodine remain widespread. These deficiencies have significantly impacted children's growth and immune function in these regions, leading to a substantial public health burden (4, 5). Nutritional deficiencies are a key factor affecting child health, with millions of children dying annually from nutrition-related diseases (6). Despite significant progress in nutrition interventions, food fortification, and supplementation over the past few decades, particularly in public health sectors of low-income countries, the overall burden of childhood nutritional deficiencies has been on the decline globally (7, 8).

Many countries have improved children's awareness of healthy diets and reduced the incidence and mortality of nutritional deficiencies by strengthening community-based nutrition interventions and school nutrition programs (9, 10). However, existing research has primarily focused on certain nutrient deficiencies and specific regions (11, 12). While these studies provide valuable data, most lack a comprehensive analysis of children as a population, and in-depth exploration remains insufficient. This has led to a lack of a thorough understanding of the burden of childhood malnutrition across different regions and income levels, and has failed to effectively provide policymakers with complete intervention guidelines. The health disparities between different regions globally, especially cross-national health inequalities, remain an important issue that needs further exploration in the public health field.

To provide further epidemiological evidence, track the progress of malnutrition disease management, and implement targeted prevention and control strategies, it is essential to conduct a comprehensive analysis of childhood malnutrition across cross-national health inequalities. Therefore, we utilized data from the 2021 Global Burden of Disease, Injuries, and Risk Factors Study (GBD) to report the incidence, prevalence, DALYs, and mortality rates of global childhood malnutrition from 1990 to 2021, as well as disease burden stratified by age group and Sociodemographic Index (SDI) across regions and globally. Additionally, we analyzed cross-national health inequalities in childhood malnutrition burden and projected the global trend of childhood malnutrition burden up to 2035.

## Methods

### Data acquisition

This study utilizes data from the GBD 2021 database, a comprehensive resource assesseing the global and regional impact of 371 diseases, injuries, and conditions, as well as 88 risk factors, across 204 countries and territories from 1990 to 2021 (13). The GBD 2021 dataset integrates contributions from 11,500 collaborators worldwide, providing detailed insights into the incidence, prevalence, mortality, and disability-adjusted life years (DALYs) associated with various health conditions. For this analysis, specific data on nutritional deficiencies were extracted from the Global Health Data Exchange (GHDx) platform (https://vizhub.healthdata.org/gbd-results/), including information on incidence, prevalence, DALYs, and mortality, disaggregated by age, gender, and geographic location. To explore the impact of economic and social factors on the burden of nutritional deficiencies, additional data (https://ghdx.healthdata.org/gbd-2021) were retrieved, including the SDI. The SDI is a composite measure based on per capita income, education level, and fertility rate, which categorizes countries into five development levels: low, low-middle, middle, high-middle, and high, providing a framework for analyzing health disparities across different socio-economic contexts.

### Data sources and disease model

In the GBD 2021, the modeling of nutritional deficiencies relied on multiple data sources, including vital registration data, verbal autopsy data, and monitoring data, all of which were cleaned and standardized to ensure accuracy. Different types of nutritional deficiencies were defined using specific ICD codes, such as protein-energy malnutrition (PEM) with codes E40-E46.9, vitamin B12 deficiency anemia with codes D51-D52.0, and iodine deficiency, which focuses on symptoms like visible goiter and intellectual disabilities. The modeling strategy primarily employed the CODEm model, which first generated overall mortality estimates for nutritional deficiencies, and then broke these down into subcategories to ensure that data trends for different age groups were accurately captured. Additionally, vitamin A deficiency, iodine deficiency, and iron deficiency were modeled using the ST-GPR and DisMod-MR 2.1 models, integrating relevant socio-economic and health service data to enhance the accuracy of prevalence estimates. For PEM, the classification was based on weight-for-height *z*-scores (WHZ), differentiating between states with and without edema to further refine disease burden estimates. The model parameters, such as smoothing coefficients, regression weights, and priors for the Bayesian models, were calibrated using a maximum likelihood approach. The model assumptions were validated through cross-validation techniques to assess predictive performance against observed data. Additional modeling details are provided in the GBD 2021 methods appendices (https://www.healthdata.org/gbd/methods-appendices-2021).

### Estimation of disease burden

In this study, data on nutritional deficiencies in children were extracted and stratified into three age groups according to the World Health Organization's child age classification standards: <5, 5–9, and 10–14 years. In 2021, the impact of nutritional deficiencies on disease burden was assessed using key indicators such as incidence, prevalence, DALYs, and mortality. The study analyzed the influence of various demographic characteristics (such as age and gender) and socio-economic factors (such as SDI) on the global and regional distribution of disease burden. To assess long-term trends, the estimated annual percentage change (EAPC) from 1990 to 2021 was calculated. The EAPC was computed based on the natural logarithm of DALYs and mortality rates, using a log-linear regression model with the formula: ln(ASR) = *a* + *bx* + *e*, where ln(ASR) is the natural logarithm of the age-standardized rate (ASRs), *x* is the year, *a* is the intercept, *b* is the slope, and *e* is the error term. The formula for EAPC is: EAPC = (exp(*b*) – 1) × 100, with the 95% confidence interval (CI) estimated through regression. If the upper limit of the CI is below 0, the trend is considered to be decreasing; if the lower limit of the CI is above 0, the trend is considered to be increasing (14).

### Decomposition analysis

To explore the drivers of changes in the disease burden of nutritional deficiencies, the study employed the Das Gupta decomposition analysis method, which assessed the specific impact of demographic aging, population growth, and epidemiological changes on disease burden over the past few decades (15). This method allows for the independent analysis of each factor's contribution to changes in disease burden while holding other variables constant. For example, the analysis examined how factors such as aging and population growth have influenced the changes in the burden of micronutrient deficiencies, quantifying the extent of their impact. Through this analysis, the combined effects of these factors are clearly revealed, helping to understand how they collectively shape trends in disease burden. Unlike traditional linear regression methods, decomposition analysis focuses on independently assessing the role of each factor, providing more precise scientific evidence for developing effective interventions for nutritional deficiencies.

### Predictive analysis

To guide public health policy and resource allocation, we employed the Bayesian age-period-cohort (BAPC) analysis model to predict the future burden of nutritional deficiencies in children. The BAPC model, implemented using the INLA (23.09.09) and BAPC (0.0.36) packages in R, allowed us to forecast the incidence, prevalence, DALYs and mortality of disease through 2035. This model considers the effects of age, period, and cohort, providing a comprehensive approach to understanding future trends in disease burden.

### Cross-country inequality analysis

This study utilized the slope inequality index (SII) and concentration index (CI), as defined by the World Health Organization, to assess health burden inequalities across countries and regions (16). The SII measures absolute inequality by performing regression analysis on the relationship between DALYs and the relative position of the SDI. The concentration index, on the other hand, evaluates relative inequality by matching the cumulative proportion of DALYs with the cumulative population distribution sorted by SDI, and performing numerical integration through the Lorenz curve. Both indices offer valuable insights into the extent and direction of health inequalities within and between countries. The SII is particularly useful for understanding disparities in absolute terms, while the CI captures relative inequalities, highlighting the unequal distribution of health outcomes across socio-economic strata. To better control for bias and heterogeneity, the study employed robust regression models (rlm) instead of conventional linear regression models (lm), which helped minimize the influence of data heterogeneity or extreme values, providing a more accurate representation of health inequality. Additionally, the study compared changes in health inequality between 1990 and 2021 across 204 countries and territories, offering a comprehensive analysis of global health disparities.

### Statistics analysis

In this study, the incidence, prevalence, DALYs, and mortality were presented as predictions per 100,000 population, including their 95% confidence intervals (CIs), and the EAPCs were also presented with their 95% CIs. All analyses and graphical representations were performed using the statistical computing software RStudio (Version 4.3.3 for Windows) and JD_GBDR (V2.22, Jingding Medical Technology Co., Ltd.). A *P*-value of < 0.05 was considered statistically significant.

### Ethical approval

For GBD studies, the Institutional Review Board of the University of Washington reviewed and approved a waiver of informed consent (https://www.healthdata.org/research-analysis/gbd).

## Results

### Global trends

**Figure 1:**
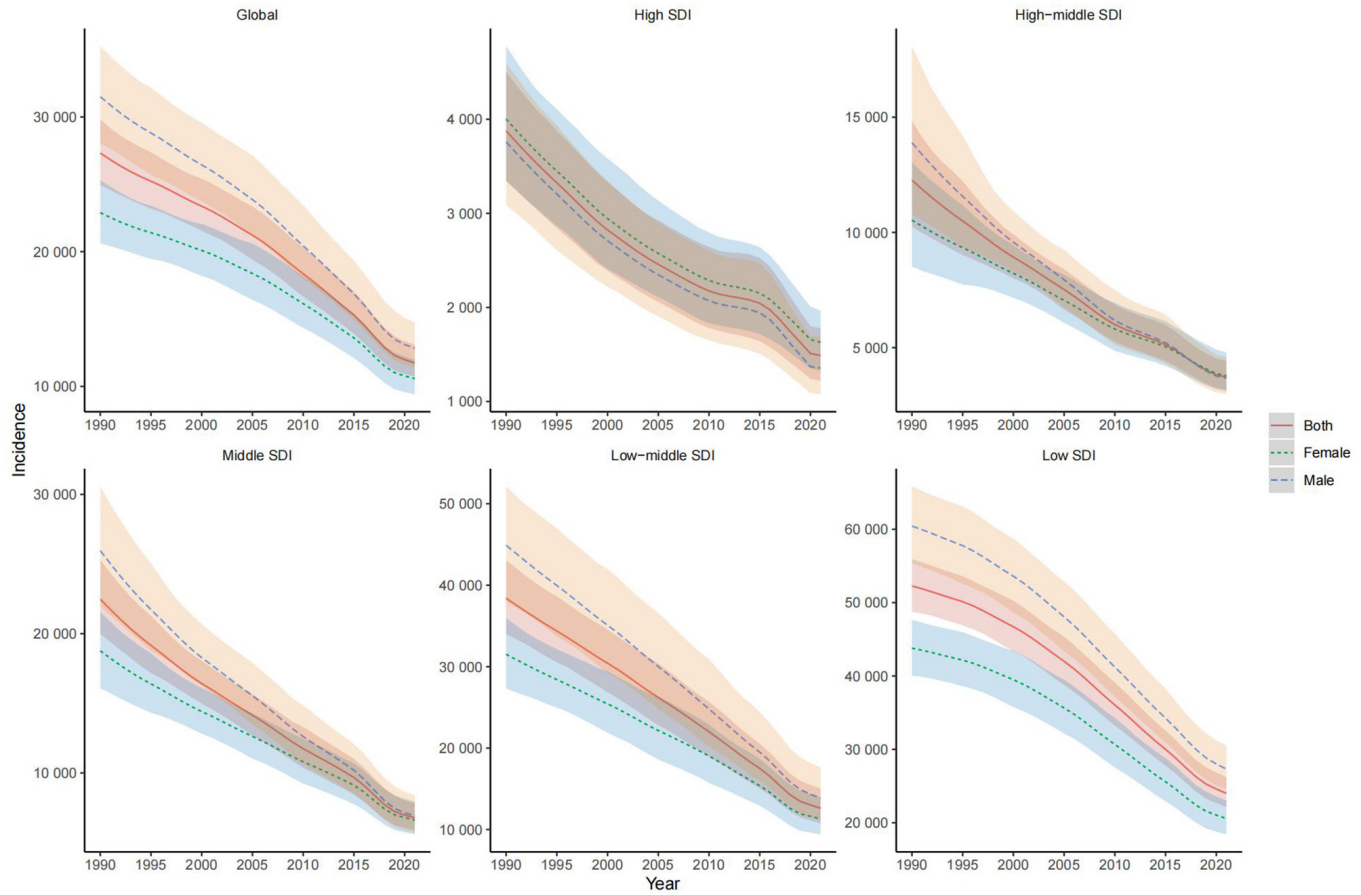
Temporal trend of age standardized incidence rate of nutritional deficiencies, globally and by sociodemographic index, from 1990 to 2021.

In 2021, the global incidence of nutritional deficiencies in children was 231.62 million cases (95% CI: 211.12 million, 258.47 million), a decrease of 51.51% compared to 1990. The ASIR was 11,741.75 cases per 100,000, with an average annual decline of 2.66% since 1990 (Table 1, Figure 1). The global prevalence in 2021 was 576.75 million cases (95% CI: 281.42 million, 301.37 million), down 18.44% from 1990. However, the ASPR was 29,138.41 cases per 100,000, with an average annual decline of 1.05% over the same period (Supplementary Table S1, Supplementary Figure S1). For DALYs related to children's nutritional deficiencies, the global total in 2021 was 22.78 million (95% CI: 17.19 million, 30.10 million), a decrease of 59.57% from 1990. The ASDR was 1,169.19 per 100,000, showing an average annual decline of 3.38% since 1990 (Supplementary Table S2, Supplementary Figure S2). The number of deaths in children due to nutritional deficiencies in 2021 reached 83,824.62 (95% CI: 64,402.08, 104,264.06), a decrease of 80.56% from 1990. However, the ASMR was 4.42 per 100,000, with an average annual decline of 5.52% over the same period (Supplementary Table S3, Supplementary Figure S3). Over the past three decades, significant regional differences were observed in ASIR, ASPR, ASDR, and ASMR, particularly between different SDI levels and genders (Figures 1, Supplementary Figure S1–S3).

**Table 1:** Age standardized incidence rate (ASIR) of nutritional deficiencies in 1990 and 2021, and estimated annual percentage change (EAPC) from 1990 to 2021 at the global and regional level.

**Group**	**1990**		**2021**		**1990−2021**
	**Incident cases, 000s (95% CI)**	**ASIRs per 100,000 (95% CI)**	**Incident cases, 000s (95% CI)**	**ASIRs per 100,000 (95% CI)**	**EAPC, % (95% CI)**
Global	477,650.347 (436,460.205, 520,784.672)	27,319.179 (24,955.091, 29,797.052)	231,618.972 (211,115.521, 258,467.093)	11,741.747 (10,707.77, 13,090.378)	−2.660 (−2.885, −2.433)
**SDI**					
High	7,146.24 (6,151.584, 8,310.725)	3,877.9 (3,341.956, 4,504.555)	2,537.38 (2,075.281, 3,033.406)	1,489.527 (1,219.622, 1,778.755)	−2.760 (−2.897, −2.623)
High-middle	33,350.963 (27,813.971, 40,369.831)	12,267.493 (10,245.319, 14,830.448)	8,428.116 (7,161.541, 10,034.537)	3,725.366 (3,172.129, 4,423.754)	−3.735 (−3.853, −3.617)
Middle	129,455.909 (115,019.412, 145,773.294)	22,471.991 (19,970.913, 25,298.597)	37,300.995 (32,329.145, 43,164.05)	6,778.42 (5,882.407, 7,826.814)	−3.660 (−3.848, −3.472)
Low-middle	184,012.179 (163,096.902, 205,971.958)	38,394.129 (33,976.635, 43,049.22)	71,860.926 (61,196.814, 85,798.732)	12,612.269 (10,757.699, 15,031.603)	−3.507 (−3.736, −3.278)
Low	123,348.301 (115,251.032, 131,756.43)	52,266.288 (48,742.714, 55,922.308)	111,324.543 (102,675.095, 121,453.773)	24,008.26 (22,135.14, 26,204.855)	−2.578 (−2.789, −2.367)
**Regions**					
Andean Latin America	2,158.037 (1,737.477, 2,651.087)	14,474.985 (11,641.117, 17,798.325)	1,040.385 (788.103, 1,356.054)	5,766.542 (4,374.937, 7,505.901)	−3.311 (−3.554, −3.068)
Australasia	34.849 (25.403, 46.017)	766.572 (558.753, 1,011.13)	28.471 (20.944, 37.413)	501.174 (369.289, 656.847)	−0.929 (−1.147, −0.709)
Caribbean	1,868.283 (1,573.081, 2,207.24)	16,244.184 (13,645.341, 19,232.995)	1,003.619 (765.36, 1,322.006)	8,798.114 (6,726.49, 11,565.157)	−2.147 (−2.221, −2.074)
Central Asia	3,070.178 (2,532.896, 3,683.032)	11,971.857 (9,848.875, 14,421.026)	1,781.511 (1,446.8, 2,160.226)	6,384.989 (5,180.682, 7,748.365)	−2.104 (−2.284, −1.924)
Central Europe	5,879.197 (5,082.268, 6,800.183)	20,227.311 (17,540.182, 23,326.965)	1,391.07 (1,185.805, 1,629.143)	7,925.541 (6,771.768, 9,262.209)	−3.152 (−3.284, −3.020)
Central Latin America	12,006.235 (10,117.443, 14,094.22)	18,594.977 (15,657.379, 21,847.507)	4,304.182 (3,321.977, 5,717.532)	6,889.182 (5,350.399, 9,097.286)	−3.090 (−3.179, −3.001)
Central Sub-Saharan Africa	12,786.568 (10,863.838, 14,815.275)	48,736.559 (40,956.416, 56,964.837)	17,838.402 (14,384.719, 21,975.447)	30,225.604 (24,324.477, 37,294.973)	−1.466 (−1.881, −1.050)
East Asia	61,142.423 (44,765.461, 80,497.884)	18,527.901 (13,567.023, 24,403.691)	10,306.918 (7,396.883, 14,339.426)	3,890.708 (2,816.426, 5,383.596)	−4.806 (−4.931, −4.680)
Eastern Europe	1,372.873 (1,068.762, 1,684.629)	2,719.605 (2,117.044, 3,335.952)	510.109 (384.549, 652.896)	1,570.53 (1,185.114, 2,005.464)	−1.395 (−1.629, −1.161)
Eastern Sub-Saharan Africa	48,944.744 (45,623.444, 52,347.15)	52,710.613 (48,987.913, 56,525.548)	41,911.47 (37,480.594, 46,667.617)	23,372.71 (20,892.624, 26,035.414)	−2.797 (−2.984, −2.610)
High-income Asia Pacific	1,200.693 (919.441, 1,530.641)	3,562.434 (2,747.134, 4,509.038)	345.187 (257.496, 441.058)	1,623.23 (1,212.466, 2,064.755)	−2.150 (−2.304, −1.996)
High-income North America	1,398.38 (1,062.747, 1,799.976)	2,270.479 (1,725.152, 2,922.249)	744.364 (550.763, 998.261)	1,137.803 (843.607, 1,523.053)	−2.210 (−2.529, −1.890)
North Africa and Middle East	26,931.357 (24,568.467, 29,572.492)	18,925.178 (17,252.542, 20,799.493)	15,143.533 (13,121.282, 17,439.478)	8,402.443 (7,289.864, 9,661.5)	−2.541 (−2.802, −2.279)
Oceania	699.815 (589.812, 837.294)	25,680.119 (21,589.192, 30,810.558)	824.655 (655.634, 1,046.242)	15,784.8 (12,508.744, 20,105.083)	−1.143 (−1.330, −0.956)
South Asia	173,039.269 (143,852.475, 203,962.332)	39,434.721 (32,699.506, 46,582.173)	61,670.806 (46,151.063, 83,019.968)	12,697.176 (9,573.887, 16,956.942)	−3.497 (−3.821, −3.172)
Southeast Asia	56,478.326 (49,400.755, 63,931.809)	33,336.025 (29,196.313, 37,686.821)	16,998.486 (13,919.528, 20,757.741)	10,086.446 (8,276.635, 12,278.2)	−3.657 (−3.777, −3.537)
Southern Latin America	2,418.544 (1,859.107, 3,079.277)	16,250.165 (12,507.052, 20,670.429)	1,088.562 (736.448, 1,560.077)	7,618.617 (5,207.238, 10,838.114)	−2.307 (−2.506, −2.108)
Southern Sub-Saharan Africa	6,216.306 (5,242.08, 7,384.783)	29,867.663 (25,140.626, 35,547.659)	3,137.307 (2,485.648, 3,965.102)	13,177.49 (10,462.903, 16,624.9)	−2.572 (−2.653, −2.490)
Tropical Latin America	14,674.237 (11,554.353, 18,143.107)	27,491.296 (21,751.97, 33,840.354)	5,326.335 (3,489.384, 7,717.455)	10,627.134 (6,973.633, 15,383.695)	−3.222 (−3.334, −3.109)
Western Europe	2,246.766 (1,919.737, 2,633.274)	3,206.203 (2,742.016, 3,752.851)	971.14 (793.965, 1,166.547)	1,438.304 (1,177.197, 1,725.138)	−2.003 (−2.225, −1.781)
Western Sub-Saharan Africa	43,083.266 (40,315.393, 46,323.967)	47,712.666 (44,580.626, 51,403.974)	45,252.459 (40,818.019, 50,008.387)	20,761.969 (18,713.122, 22,969.481)	−2.719(−2.800, −2.638)

ASIR, age standardized incidence rate; EAPC, estimated annual percentage change; SDI, socio-demographic index; 95% CI, 95% confidence interval.

### Regional trends

In 2021, analysis of 21 GBD regions revealed regional differences in the incidence, prevalence, DALYs, and deaths related to children's nutritional deficiencies. Central Sub-Saharan Africa (30,225.60), Eastern Sub-Saharan Africa (23,372.71), and Western Sub-Saharan Africa (20,761.97) reported the highest ASIR, whereas Australasia (501.17), High-income North America (1,137.80), and Western Europe (1,438.30) had the lowest ASIR (Table 1). Meanwhile, Western Sub-Saharan Africa (45,290.90), Central Sub-Saharan Africa (43,548.74), and South Asia (43,395.25) reported the highest ASPR, while High-income North America (3,203.87), Australasia (4,693.40), and High-income Asia Pacific (4,812.74) had the lowest ASPR (Supplementary Table S1). Additionally, Southern Sub-Saharan Africa (2,485.63), Eastern Sub-Saharan Africa (2,254.98), and Western Sub-Saharan Africa (2,171.98) reported the highest ASDR, while High-income Asia Pacific (42.61), Australasia (45.51), and High-income North America (52.66) had the lowest ASDR (Supplementary Table S2). In terms of mortality, Southern Sub-Saharan Africa (16.35), Eastern Sub-Saharan Africa (15.46), and Western Sub-Saharan Africa (9.76) had the highest ASMR, whereas Australasia (0.01), High-income Asia Pacific (0.02), and Western Europe (0.02) had the lowest ASMR (Supplementary Table S3). Supplementary Figure S4 displays the gender distribution of ASIR, ASPR, ASDR, and ASMR across GBD regions in 2021, and Supplementary Figure S5 shows the distribution differences of these indicators in 1990 and 2021 across regions.

From 1990 to 2021, the EAPC in ASIR was most significant in East Asia (−4.81%), Southeast Asia (−3.66%), and South Asia (−3.50%; Table 1). The trends in changes for ASPR, ASDR, and ASMR were similar, as shown in Supplementary Tables S1–S3. The EAPCs for ASIR, ASPR, ASDR, and ASMR by gender across regions are shown in Supplementary Figure S6.

### National trends

**Figure 2:**
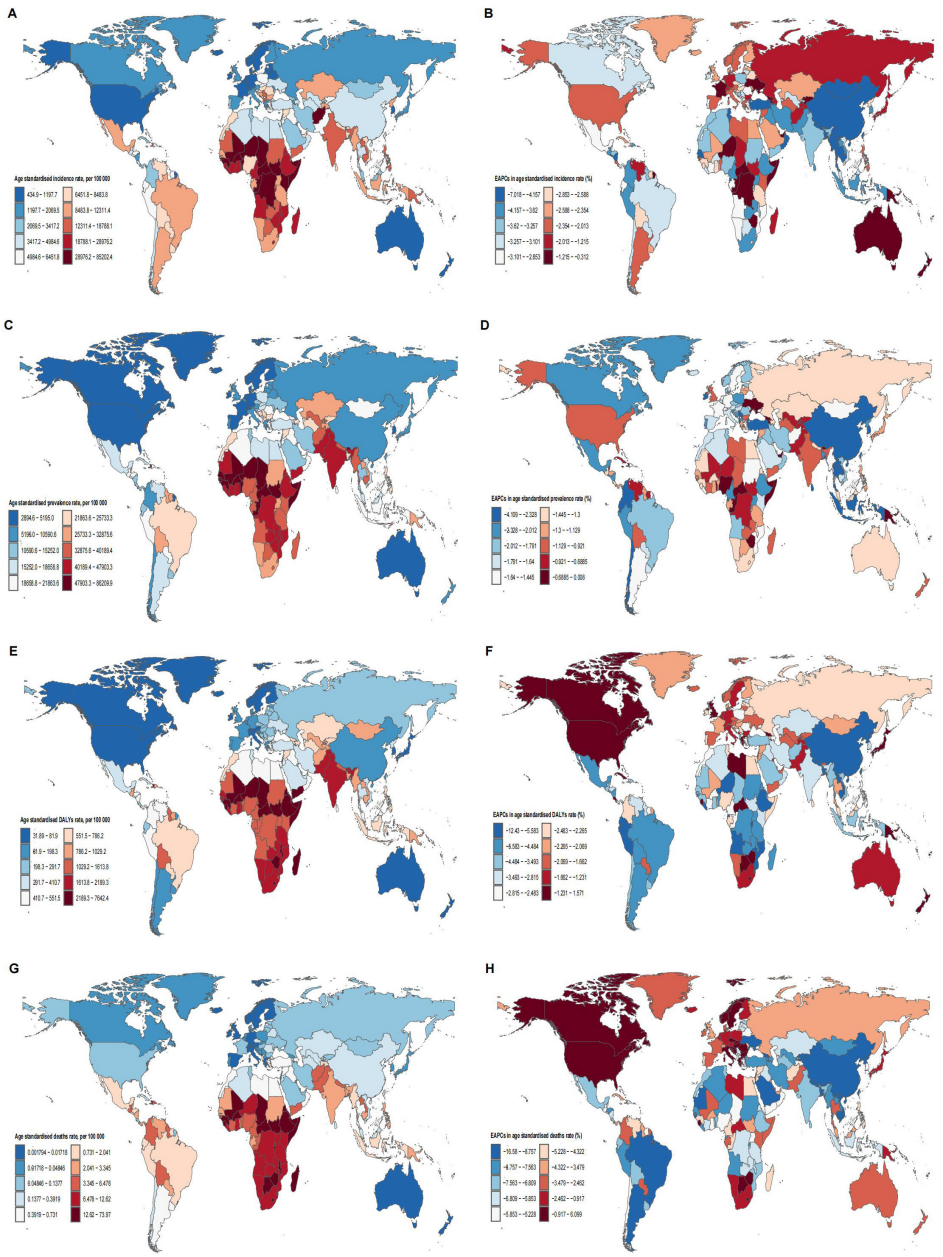
Map of **(A)** age standardized incidence rate in 2021; **(B)** EAPC in age standardized incidence rate from 1990 to 2021; **(C)** age standardized prevalence rate in 2021; **(D)** EAPC in age standardized prevalencee rate from 1990 to 2021; **(E)** age standardized DALYs rate in 2021; **(F)** EAPC in age standardized DALYs rate from 1990 to 2021; **(G)** age standardized deaths rate in 2021; **(H)** EAPC in age standardized deaths rate from 1990 to 2021; of nutritional deficiencies for 204 countries and territories.

In 2021, the ASIR of children's nutritional deficiencies showed significant variation, ranging from 434.92 to 85,202.39 per 100,000. Somalia had the highest ASIR at 85,202.39, followed by Niger (63,339.43) and Chad (49,323.23). In contrast, Australia had the lowest ASIR at 434.92, with Switzerland (734.48) and Germany (778.89) also reporting low rates (Figure 2A, Supplementary Table S4). The ASPR ranged from 2,894.63 to 86,209.91 per 100,000, with Somalia (86,209.91), Niger (62,638.68), and Mali (64,482.33) reporting the highest prevalence, while Canada (2,894.63), the United States (3,235.68), and Monaco (3,452.37) had the lowest (Figure 2C, Supplementary Table S4). The highest ASDR were reported in Sierra Leone (7,642.39), Mali (6,420.61), and South Sudan (6,186.12), while Singapore (31.89), the Republic of Korea (35.05), and Australia (40.13) had the lowest (Figure 2E, Supplementary Table S4). The ASMR ranged from 0.01 to 73.97 per 100,000, with Sierra Leone (73.97), South Sudan (57.17), and Mali (48.30) having the highest, while Andorra, Greece, and Austria had the lowest at 0.01 (Figure 2G, Supplementary Table S4).

From 1990 to 2021, the countries with the greatest decline in ASIR were Equatorial Guinea (−7.02%), Maldives (−6.49%), and Nicaragua (−6.28%; Figure 2B, Supplementary Table S4). The fastest decline in ASPR was observed in China (−4.11%), Equatorial Guinea (−3.38%), and the Republic of Korea (−3.16%; Figure 2D, Supplementary Table S4). However, the largest increase in ASDR occurred in Zimbabwe (1.57%), Canada (1.38%), and the United States (0.71%), while the greatest decreases were seen in the Democratic People's Republic of Korea (−12.43%), Bangladesh (−8.04%), and Angola (−7.52%; Figure 2F, Supplementary Table S4). ASMR saw the most significant increases in Norway (6.10%), Bosnia and Herzegovina (2.60%), and Zimbabwe (2.12%), while the largest decreases were observed in the Democratic People's Republic of Korea (−16.58%), China (−11.75%), and Bangladesh (−11.27%; Figure 2H, Supplementary Table S4).

### Association with the SDI

**Figure 3:**
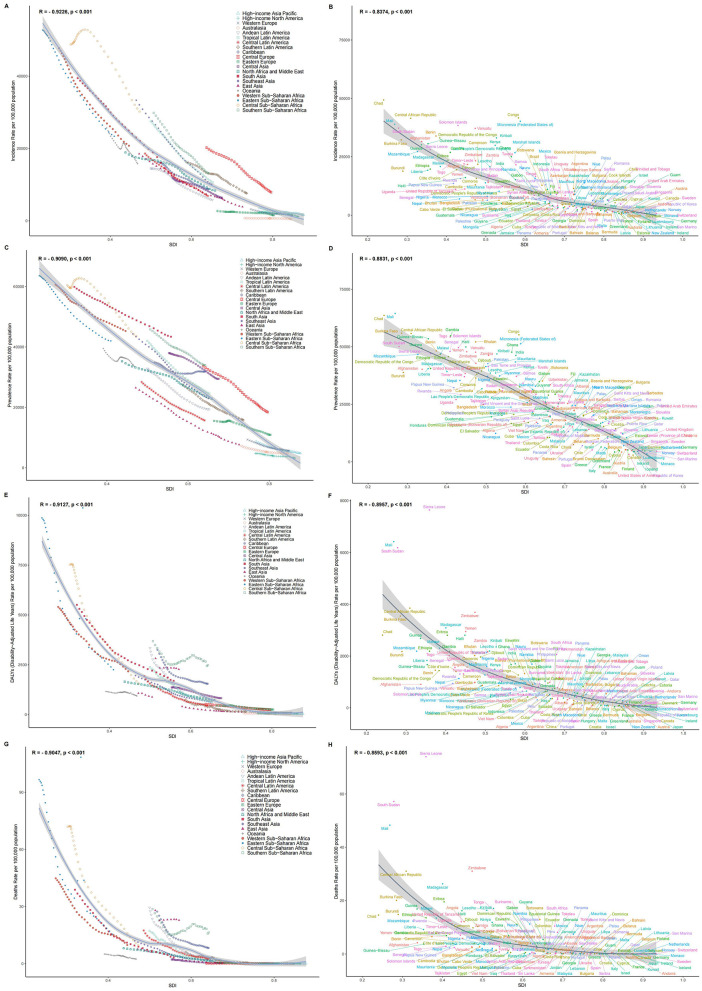
**(A)** Age standardized incidence rate for 21 regions from 1990 to 2021 by sociodemographic index; **(B)** age standardized incidence rate for 204 countries and territories in 2021 by sociodemographic index; **(C)** age standardized prevalence rate for 21 regions from 1990 to 2021 by sociodemographic index; **(D)** age standardized prevalence rate for 204 countries and territories in 2021 by sociodemographic index; **(E)** age standardized DALYs rate for 21 regions from 1990 to 2021 by sociodemographic index; **(F)** age standardized DALYs rate for 204 countries and territories in 2021 by sociodemographic index; **(G)** age standardized deaths rate for 21 regions of for 21 regions from 1990 to 2021 by sociodemographic index; **(H)** age standardized deaths rate for 204 countries and territories in 2021 by sociodemographic index.

At the regional level, from 1990 to 2021, there was a significant negative correlation between the ASIR of children's nutritional deficiencies and the SDI. Specifically, the ASIR in Central Sub-Saharan Africa was significantly higher than expected based on its SDI, while Oceania's ASIR consistently remained below the expected value (Figure 3A). Similar patterns were observed for the relationships between SDI and the ASPR, ASDR, and ASMR (Figures 3C, E, G).

**Figure 4:**
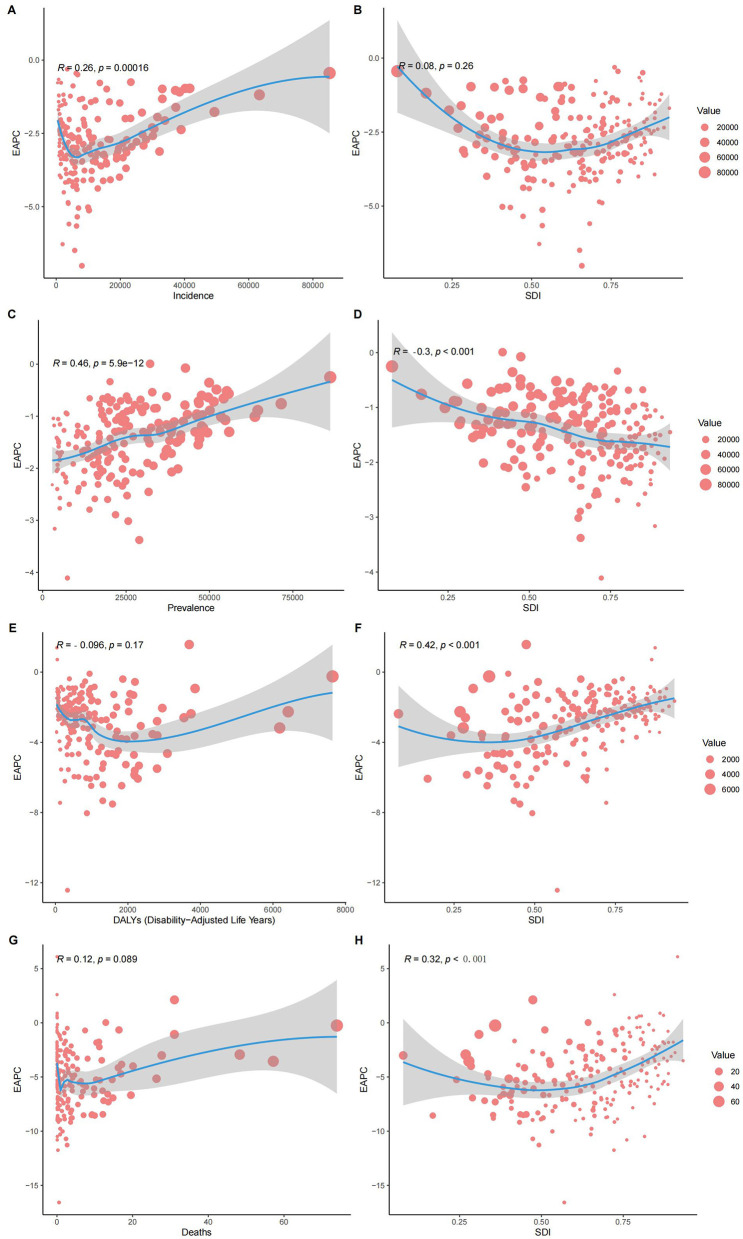
Correlation of **(A)** EAPC and age standardized incidence rate; **(B)** EAPC and age standardized incidence rate; **(C)** EAPC and age standardized prevalence; **(D)** EAPC and age standardized prevalence; **(E)** EAPC and standardized DALYs rates; **(F)** EAPC and standardized DALYs rates; **(G)** EAPC and standardized mortality rates; **(H)** EAPC and standardized mortality rates, for 204 countries and territories.

At the national level, in 2021, the ASIR for children's nutritional deficiencies generally decreased with increasing SDI. Somalia's ASIR was much higher than expected, while Australia's ASIR was significantly lower than expected (Figure 3B). Similar national-level patterns were observed for the relationships between SDI and ASPR, ASDR, and ASMR (Figures 3D, F, H). The relationship between the estimated annual percentage change (EAPC) and ASRs showed a positive correlation (Figures 4A, C, E, G). Furthermore, the EAPC for ASIR, ASDR, and ASMR had a positive correlation with SDI (Figures 4B, F, H), while the EAPC for ASPR showed a negative correlation with SDI (Figure 4D).

### Decomposition analysis

**Figure 5:**
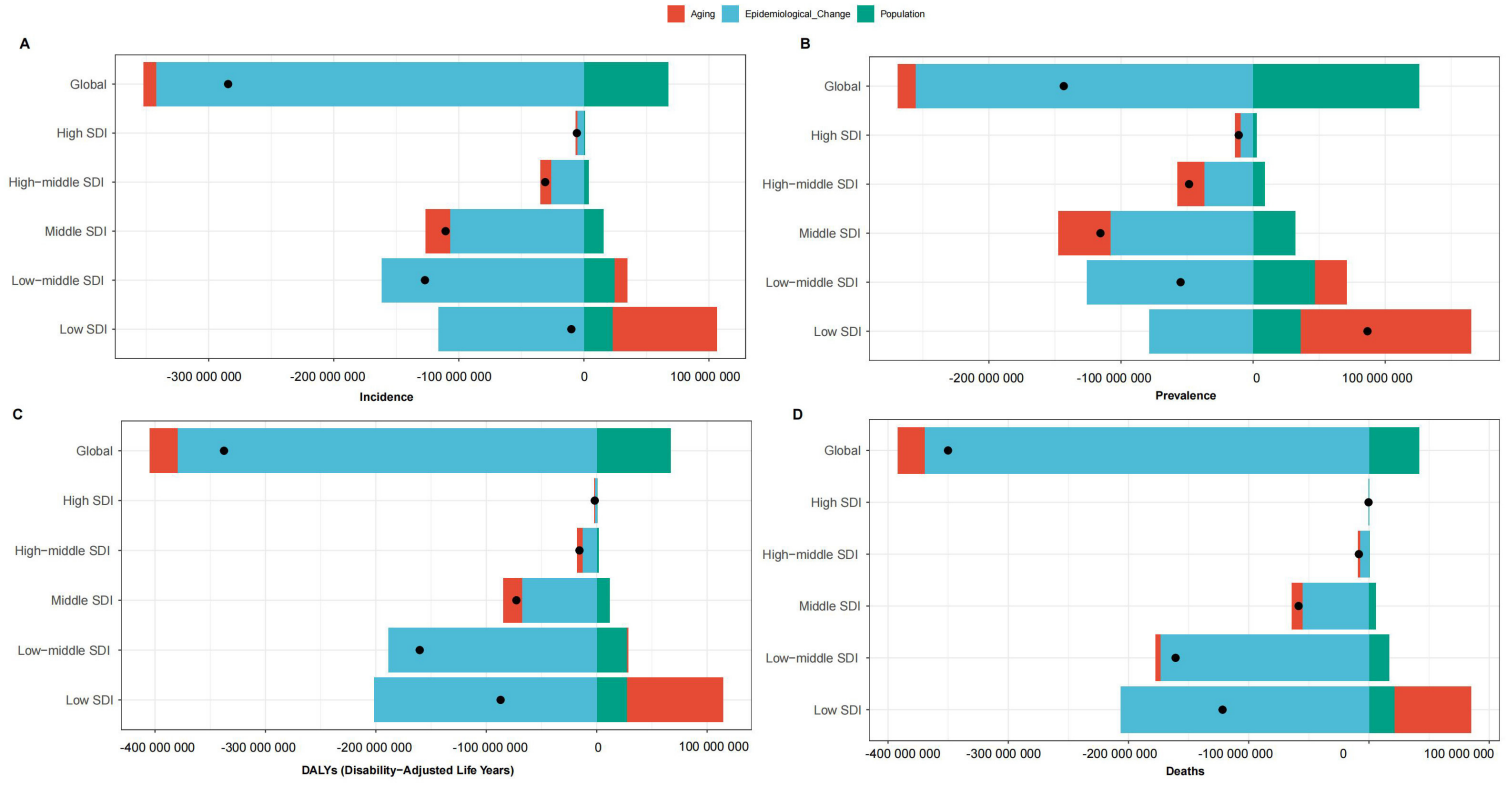
Decomposition analysis of **(A)** incidence cases; **(B)** prevalence cases; **(C)** DALYs cases; **(D)** deaths cases of nutritional deficiencies globally and SDI levels from 1990 to 2021.

To explore the drivers of changes in the disease burden of children's nutritional deficiencies, we assessed the specific impact of demographic aging, population growth, and epidemiological changes over the past few decades. Specifically, between 1990 and 2021, the global decline in incident cases of children's nutritional deficiencies was primarily driven by epidemiological changes (Figure 5A). This pattern was similarly observed for prevalent cases, DALY cases, and death cases, where epidemiological change was the dominant factor driving the decline (Figures 5B–D). In regions with different SDI levels, we observed that the decline in incident cases, prevalent cases, DALY cases, and death cases was also predominantly driven by epidemiological change.

### Predictive analysis

**Figure 6:**
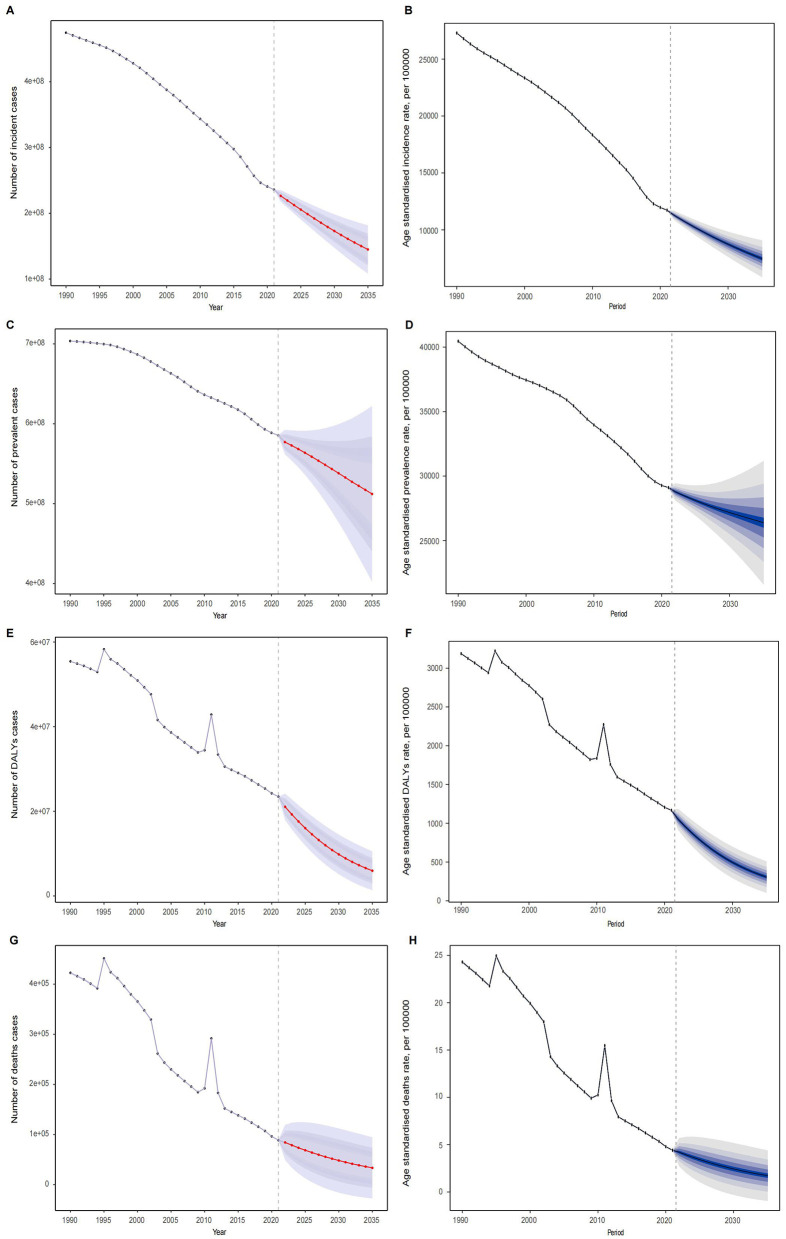
Predictive analysis of **(A)** incidence cases; **(B)** age standardized incidence cases; **(C)** prevalence cases; **(D)** age standardized prevalence cases; **(E)** DALYs cases; **(F)** age standardized DALYs cases; **(G)** deaths cases; **(H)** age standardized deaths cases of nutritional deficiencies globally from 1990 to 2035.

Figure 6 provides detailed predictions for the incidence, prevalence, DALYs, and mortality rates of children's nutritional deficiencies by 2035. It is expected that the global total for incidence, prevalence, DALYs, and deaths will gradually decrease, with projections of 144,982,655.55 incident cases, 512,145,321.77 prevalent cases, 5,957,750.19 DALYs, and 33,512.68 deaths by 2035 (Figures 6A, C, E, G). In contrast, the age-standardized rates (ASRs) for these indicators are expected to show an average annual decline (Figures 6B, D, F, H). By 2035, the ASIR, ASPR, ASDR, and ASMR are projected to decrease to 7,469.67, 26,386.33, 306.95, and 1.73 per 100,000, respectively. Additionally, the predictions for cases and rates for different age groups are shown in Supplementary Figures S7, S8.

### Cross-country inequality analysis

**Figure 7:**
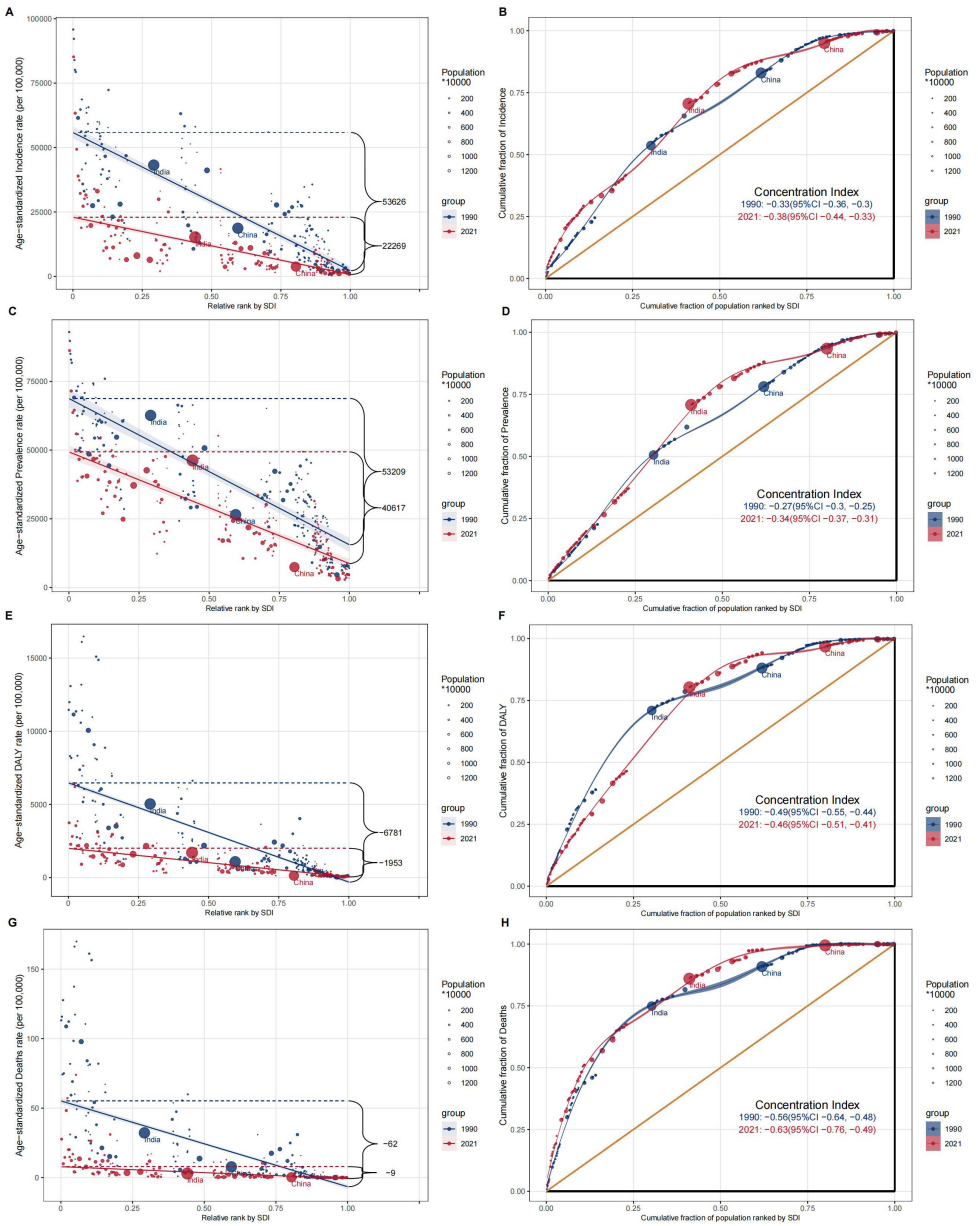
Cross-country inequality analysis of **(A, B)** incidence cases; **(C, D)** prevalence cases; **(E, F)** DALYs cases; **(G, H)** deaths cases of nutritional deficiencies for 204 countries and territories.

Significant absolute and relative inequality related to SDI was observed in the burden of children's nutritional deficiencies, with these disparities showing a notable decline over time (Figure 7). Notably, incidence rates were disproportionately concentrated in countries with lower SDI. As indicated by the inequality slope index, the gap in prevalence between the highest and lowest SDI countries decreased from 53,626 per 100,000 in 1990 to 22,269 per 100,000 in 2021 (Figures 7A, B). However, the concentration index, which measures relative inequality, expanded from −0.33 in 1990 to −0.38 in 2021. Similar patterns were observed for prevalence (Figures 7C, D), DALYs (Figures 7E, F), and deaths (Figures 7G, H), highlighting the unequal distribution of the burden of children's nutritional deficiencies across countries with different SDI levels.

## Discussion

### Principal findings

From 1990 to 2021, the global burden of children's nutritional deficiencies showed significant changes, with a 51.51% decrease in incidence, an 18.44% reduction in prevalence, a 59.57% decrease in DALYs, and an 80.56% decrease in deaths. Despite these changes, ASIR, ASPR, ASDR, and ASMR continued to decline globally, reflecting some success in public health interventions. Significant regional disparities were observed, with countries in Sub-Saharan Africa, such as Somalia, reporting much higher ASIR and ASPR compared to high-SDI countries like Australia and Switzerland, which had significantly lower rates. Similar trends were observed for ASDR, ASMR, and DALYs, with a higher burden in low-SDI regions. Projections for 2035 suggest that total incidence, prevalence, DALYs, and deaths will continue to decline, though age-standardized rates are expected to decrease, highlighting the ongoing challenge of disparities in disease burden between high- and low-SDI countries. These findings underscore the need for region-specific strategies to reduce inequalities and ensure equitable access to healthcare, especially in low-SDI regions where the burden of nutritional deficiencies remains high despite overall improvements.

### Comparison with other studies

From 1990 to 2021, the global burden of childhood nutritional deficiencies has significantly declined, with reductions in incidence, prevalence, DALYs, and mortality. This positive trend reflects the success of global public health interventions, particularly those focused on nutritional supplementation, food fortification, and gradual improvements in healthcare systems. In recent years, numerous countries worldwide have implemented extensive nutritional supplementation and fortification programs, such as those targeting vitamin A, iron, iodine, and folic acid (17). These measures have notably reduced child morbidity and mortality caused by nutritional deficiencies. Additionally, improvements in maternal health, enhanced quality and coverage of delivery healthcare services, and efforts to promote maternal and child health have further diminished the negative impacts of malnutrition on children (18). Similar findings have been observed in other studies, which report a significant reduction in child malnutrition rates in countries with strong maternal and child health programs (18). With global economic growth, especially in lower- and middle-income countries, and improvements in public health systems, more children have gained access to nutritional interventions and health monitoring. Many middle-income countries have introduced community-based nutrition programs, strengthening the management of child nutrition and further alleviating the burden of nutritional deficiencies. For example, countries such as India and China have reduced the incidence and mortality of nutritional deficiencies by strengthening school nutrition programs and promoting community health education, thus raising children's awareness of healthy eating (19).

However, despite the overall decline in the global burden of childhood nutritional deficiencies, low-income and middle-income countries continue to face significant challenges. These trends are consistent with findings from a recent study by Rahman et al. (20), which highlights the ongoing struggle in Sub-Saharan Africa and South Asia due to socio-economic barriers. Sub-Saharan Africa and South Asia remain high-burden regions, and according to the World Health Organization's report, this is closely linked to poverty, a lack of healthcare resources, and insufficient nutrition education. In these regions, childhood nutritional deficiencies remain a major contributor to child mortality (21, 22). Therefore, despite significant progress made globally, there is still a need to intensify support for these regions, particularly in improving food security and enhancing basic healthcare services. Additionally, with the intensification of global warming and climate change, food security issues are becoming increasingly severe, which may have long-term effects on children's nutritional status (23, 24). Extreme weather events, declining crop yields, and rising food prices caused by climate change may further exacerbate nutritional problems in low-income regions. These findings align with concerns raised by other researchers, who emphasized the heightened vulnerability of children in climate-affected regions to nutritional deficiencies (25). Consequently, in addition to traditional nutritional interventions, it is crucial to consider how policies can address the impact of climate change on children's health.

There are significant differences in the burden of childhood nutritional deficiencies across countries, particularly in terms of incidence rates. Somalia, Niger, and Chad have the highest rates, while Australia, Switzerland, and Germany report the lowest. These disparities highlight the uneven progress in addressing nutritional deficiencies, influenced by factors such as economic development, healthcare access, nutrition interventions, and socio-cultural factors. In countries with high incidence rates, such as Somalia, Niger, and Chad, nutritional deficiencies remain a major issue due to poverty, food insecurity, inadequate healthcare, and limited public health resources (26). A study further supports this (27), indicating that the lack of effective public health policies in these regions exacerbates the nutritional crisis. Low education levels, lack of health knowledge, and insufficient focus on children's nutrition also contribute to the problem (28). Many African countries, especially in Sub-Saharan Africa, continue to struggle with malnutrition. Studies show that children lacking key nutrients like vitamin A, iron, and iodine often suffer from growth delays, weakened immunity, and other health problems, negatively affecting their overall health and survival (29). In contrast, high-income countries like Australia, Switzerland, and Germany have lower rates of childhood nutritional deficiencies, thanks to strong public health systems, higher education levels, and widespread nutrition interventions, all of which have helped reduce the burden of malnutrition (30, 31).

The analysis of the results indicates that from 1990 to 2021, the primary driver of the reduction in the burden of childhood nutritional deficiencies was epidemiological changes. These changes influenced incidence, prevalence, DALYs, and mortality rates. This finding suggests that public health interventions, improvements in healthcare services, and enhanced nutritional status have collectively contributed to the global reduction in nutritional deficiencies. The epidemiological changes are largely linked to the expansion of targeted nutrition programs, including supplementation and food fortification strategies. These measures have played a crucial role in addressing deficiencies in micronutrients such as vitamin A, iron, and iodine (32). Similar trends have been observed in other studies, which found that supplementation programs contributed to a reduction in iron-deficiency anemia among children in low- and middle-income countries (33). These interventions have significantly reduced the incidence of severe malnutrition and its associated complications, contributing to the decline in incidence and mortality rates, and thereby reducing DALYs. Furthermore, promoting breastfeeding and nutrition education has likely played a role in improving children's nutritional status, further driving the decrease in morbidity and prevalence rates (34).

It is expected that by 2035, the global incidence, DALYs, and mortality rates of childhood nutritional deficiencies will continue to decline, with age-standardized rates also continuing to decrease. This projection reflects the ongoing global efforts and the effectiveness of public health interventions in addressing childhood nutritional deficiencies. In recent years, the progress of nutrition supplementation, food fortification, and health education, especially in low- and middle-income countries, has significantly improved children's nutritional status and reduced the burden of diseases related to nutritional deficiencies (35). Additionally, with the continuous improvement of global health systems, particularly in low- and middle-income countries, the coverage and effectiveness of nutrition intervention programs have also improved. The success of these interventions has been particularly evident in South Asia, where nutrition-focused policies have contributed to a reduction in childhood stunting rates, as highlighted by recent studies (36). Improvements in public health policies, such as strengthening maternal and child health and promoting infant feeding policies, are expected to continue to drive improvements in nutritional status over the coming decades (37, 38). These interventions not only help reduce the burden of nutritional deficiencies but also have a positive impact on children's long-term health, contributing to the sustained decline in age-standardized rates.

However, regional disparities still persist. Cross-country inequality analyses reveal significant health inequalities associated with the SDI. While absolute inequalities have somewhat decreased, relative inequalities remain prominent. This highlights the global progress in addressing childhood nutritional deficiencies, yet health gaps between different SDI regions are still evident. On one hand, as global health resources increase, the absolute burden in low-SDI regions has declined, particularly due to the widespread implementation of nutrition intervention programs, food fortification, and supplementation (39). In line with this, an analysis has demonstrated that SDI is a significant predictor of health outcomes in nutrition-related diseases (40). However, low-SDI countries still face challenges such as inadequate socioeconomic conditions, healthcare services, and health infrastructure, which result in a relatively heavier burden. Despite progress, the gap between these countries and high-income nations remains substantial. On the other hand, improvements in high-income countries have been more pronounced, driven by stronger public health systems, better nutrition interventions, and higher education levels. However, as investments in nutrition interventions continue to rise in high-SDI countries, the improvements in these nations may surpass those in low-SDI countries, leading to a widening of global health inequalities (41). Therefore, while absolute inequalities have decreased, the persistent health disparities, particularly relative inequalities between countries and regions, remain an important issue that requires ongoing attention.

### Strengths and limitations of the current study

This study provides a comprehensive analysis of the global burden of children's nutritional deficiencies using data from the GBD 2021. It offers valuable insights into trends in incidence, prevalence, DALYs, and mortality across global, regional, and national levels. By using age-standardized rates and incorporating the SDI, the study highlights health disparities and projects future trends through 2035 to guide policy and resource allocation. However, there are several limitations. Data gaps and varying case definitions in the GBD methodology may introduce bias, and confidence intervals used instead of uncertainty intervals require cautious interpretation. Limited data from low- and middle-income countries may reduce accuracy, as variations in healthcare quality and socio-economic factors could lead to underreporting. Additionally, the lack of subnational data limits the ability to assess intra-country disparities. The predictions made in the study are based on current trends, which may not fully account for unforeseen changes in socio-political or economic conditions, potentially limiting the accuracy of future projections. Despite these limitations, the study provides the most up-to-date estimates of the global burden, offering a foundation for targeted health policies.

## Conclusions

This study, using GBD 2021 data, reveals the global burden of children's nutritional deficiencies and its regional disparities. While the global burden has decreased overall, low-SDI regions continue to face significant health challenges. Globally, the incidence, prevalence, DALYs, and mortality associated with children's nutritional deficiencies have all shown a declining trend. However, the burden remains disproportionately high in low-SDI regions, particularly in Sub-Saharan Africa, highlighting the ongoing need for improved public health resources and nutrition interventions in these areas. Despite these challenges, with continued global health efforts, the burden of nutritional deficiencies in children is projected to decline further in the coming years. To further reduce this burden, especially in low-SDI regions, targeted interventions, better resource allocation, and efforts to address health inequalities are crucial. These findings should inform global nutrition policies and guide the prioritization of resources to reduce disparities in children's health worldwide.

## Data Availability

The datasets presented in this study can be found in online repositories. The names of the repository/repositories and accession number(s) can be found in the article/Supplementary material.

## Supplementary material

Supplementary Figure S1 Temporal trend of age standardized prevalence rate of of nutritional deficiencies, globally and by sociodemographic index, from 1990 to 2021.

Supplementary Figure S2 Temporal trend of age standardized DALYs rate of of nutritional deficiencies, globally and by sociodemographic index, from 1990 to 2021.

Supplementary Figure S3 Temporal trend of age standardized deaths rate of of nutritional deficiencies, globally and by sociodemographic index, from 1990 to 2021.

Supplementary Figure S4 Sex differences in **(A)** incidence; **(B)** prevalence; **(C)** DALYs; **(D)** deaths, of nutritional deficiencies for 21 regions in 2021.

Supplementary Figure S5 Year differences in **(A)** incidence; **(B)** prevalence; **(C)** DALYs; **(D)** deaths, of nutritional deficiencies for 21 regions in 1990 and 2021.

Supplementary Figure S6 EAPC of **(A)** incidence; **(B)** prevalence; **(C)** DALYs; **(D)** deaths, of nutritional deficiencies for 21 regions from 1990 to 2021.

Supplementary Figure S7 Predictive analysis for age groups cases of nutritional deficiencies globally from 2022 to 2035.

Supplementary Figure S8 Predictive analysis for age groups rates of nutritional deficiencies globally from 2022 to 2035.

## References

[1] Macdonald IA van der Beek EM Binia A. Achievements, challenges, and future direction in early life nutrition. Nestle Nutr Inst Workshop Ser. (2024) 100:1–15. 10.1159/000540138 39586241

[2] Ibrahim MK Zambruni M Melby CL Melby PC. Impact of childhood malnutrition on host defense and infection. Clin Microbiol Rev. (2017) 30:919–71. 10.1128/CMR.00119-16 28768707 PMC5608884

[3] Vassilopoulou E Venter C Roth-Walter F. Malnutrition and allergies: tipping the immune balance towards health. J Clin Med. (2024) 13:4713. 10.3390/jcm13164713 39200855 PMC11355500

[4] Harika R Faber M Samuel F Mulugeta A Kimiywe J Eilander A. Are low intakes and deficiencies in iron, vitamin A, zinc, and iodine of public health concern in Ethiopian, Kenyan, Nigerian, and South African Children and adolescents? Food Nutr Bull. (2017) 38:405–27. 10.1177/0379572117715818 28682645

[5] Armitage AE Moretti D. The importance of iron status for young children in low- and middle-income countries: a narrative review. Pharmaceuticals. (2019) 12:59. 10.3390/ph12020059 30995720 PMC6631790

[6] Darnton-Hill I Kennedy E Cogill B Hossain SM. Solutions to nutrition-related health problems of preschool children: education and nutritional policies for children. J Pediatr Gastroenterol Nutr. (2006) 43(Suppl 3):S54–65. 10.1097/01.mpg.0000255851.30400.e7 17204980

[7] World Health Organization (WHO). Reducing Stunting in Children: Equity Considerations for Achieving the Global Nutrition Targets 2025. World Health Organization (2018). Available online at: https://apps.who.int/iris/bitstream/handle/10665/260202/9789241513647-eng.pdf (Accessed April 12, 2025).

[8] Scott N Delport D Hainsworth S Pearson R Morgan C Huang S. Ending malnutrition in all its forms requires scaling up proven nutrition interventions and much more: a 129-country analysis. BMC Med. (2020) 18:356. 10.1186/s12916-020-01786-5 33183301 PMC7661178

[9] Shenavar R Sajjadi SF Farmani A Zarmehrparirouy M Azadbakht L. Improvement in anthropometric measurements of malnourished children by means of complementary food and nutritional education in Fars province, Iran: a community-based intervention. Front Nutr. (2022) 9:813449. 10.3389/fnut.2022.813449 35308266 PMC8924542

[10] Pappas G Agha A Rafique G Khan KS Badruddin SH Peermohamed H. Community-based approaches to combating malnutrition and poor education among girls in resource-poor settings: report of a large scale intervention in Pakistan. Rural Remote Health. (2008) 8:820. 10.22605/RRH820 18785799

[11] Fernández-Gaxiola AC Neufeld LM García-Guerra A. Considerations for correction of micronutrient deficiencies through supplementation in pregnant women and children under-5 in Latin America. Food Nutr Bull. (2024) 45(2_suppl):S47–54. 10.1177/03795721231219824 38186006

[12] Chamanoor M Juneja RK Sami S Arefin S Al-Sabbagh D Thota AN. Disparities in place of death among malnourished individuals in the United States. Cureus. (2024) 16:e55503. 10.7759/cureus.55503 38571833 PMC10990269

[13] GBD 2021 Diseases and Injuries Collaborators. Global incidence, prevalence, years lived with disability (YLDs), disability-adjusted life-years (DALYs), and healthy life expectancy (HALE) for 371 diseases and injuries in 204 countries and territories and 811 subnational locations, 1990-2021: a systematic analysis for the Global Burden of Disease Study 2021. Lancet. (2024) 403:2133–61. 10.1016/S0140-6736(24)00757-8 38642570 PMC11122111

[14] Yang F Lodder P Huang N Liu X Fu M Guo J. Thirty-year trends of depressive disorders in 204 countries and territories from 1990 to 2019: an age-period-cohort analysis. Psychiatry Res. (2023) 328:115433. 10.1016/j.psychres.2023.115433 37651839

[15] Das Gupta P. A general method of decomposing a difference between two rates into several components. Demograph. (1978) 15:99–112. 10.2307/2060493 631402

[16] World Health Organization. Handbook on Health Inequality Monitoring: with A Special Focus on Low-and Middle-income Countries. World Health Organization (2013).26387506

[17] Kashi B Godin CM Kurzawa ZA Verney AMJ Busch-Hallen JF De-Regil LM. Multiple micronutrient supplements are more cost-effective than iron and folic acid: modeling results from 3 high-burden Asian countries. J Nutr. (2019) 149:1222–9. 10.1093/jn/nxz052 31131412

[18] Keats EC Das JK Salam RA Lassi ZS Imdad A Black RE Bhutta ZA. Effective interventions to address maternal and child malnutrition: an update of the evidence. Lancet Child Adolesc Health. (2021) 5:367–84. 10.1016/S2352-4642(20)30274-1 33691083

[19] Sharma N Gupta M Aggarwal AK Gorle M. Effectiveness of a culturally appropriate nutrition educational intervention delivered through health services to improve growth and complementary feeding of infants: a quasi-experimental study from Chandigarh, India. PLoS ONE. (2020) 15:e0229755. 10.1371/journal.pone.0229755 32182241 PMC7077818

[20] Rahman MA Kundu S Rashid HO Tohan MM Islam MA. Socio-economic inequalities in and factors associated with minimum dietary diversity among children aged 6-23 months in South Asia: a decomposition analysis. BMJ Open. (2023) 13:e072775. Published 2023 Dec 20. 10.1136/bmjopen-2023-072775 38128933 PMC10749007

[21] Bain LE Awah PK Geraldine N Kindong NP Sigal Y Bernard N Tanjeko AT. Malnutrition in Sub-Saharan Africa: burden, causes and prospects. Pan Afr Med J. (2013) 15:120. 10.11604/pamj.2013.15.120.2535 24255726 PMC3830470

[22] Harding KL Aguayo VM Webb P. Hidden hunger in South Asia: a review of recent trends and persistent challenges. Public Health Nutr. (2018) 21:785–95. 10.1017/S1368980017003202 29256361 PMC5851053

[23] Ahmed Hanifi SMM Menon N Quisumbing A. The impact of climate change on children's nutritional status in coastal Bangladesh. Soc Sci Med. (2022) 294:114704. 10.1016/j.socscimed.2022.114704 35030394

[24] Ahdoot S Baum CR Cataletto MB Hogan P Wu CB Bernstein A. Climate change and children's health: building a healthy future for every child. Pediatrics. (2024) 153:e2023065505. 10.1542/peds.2023-065504 38374808

[25] Otorkpa OJ Yusuf AM Aborode AT. Climate and conflict-induced child nutrition crisis in Sub-Saharan Africa. Confl Health. (2024) 18:59. 10.1186/s13031-024-00621-5 39367467 PMC11453013

[26] Wudil AH Usman M Rosak-Szyrocka J Pilar L Boye M. Reversing years for global food security: a review of the food security situation in sub-Saharan Africa (SSA). Int J Environ Res Public Health. (2022) 19:14836. 10.3390/ijerph192214836 36429555 PMC9690952

[27] McMichael AJ Woodruff RE Hales S. Climate change and human health: present and future risks [published correction appears in Lancet. 2006 Sep 2;368(9538):842]. Lancet. (2006) 367:859–69. 10.1016/S0140-6736(06)68079-3 16530580

[28] Sanders LM Shaw JS Guez G Baur C Rudd R. Health literacy and child health promotion: implications for research, clinical care, and public policy. Pediatrics. (2009) 124(Suppl 3):S306–14. 10.1542/peds.2009-1162G 19861485

[29] Bailey RL West KP Jr Black RE. The epidemiology of global micronutrient deficiencies. Ann Nutr Metab. (2015) 66(Suppl 2):22–33. 10.1159/000371618 26045325

[30] Shahid SM Bishop KS. Comprehensive approaches to improving nutrition: future prospects. Nutrients. (2019) 11:1760. 10.3390/nu11081760 31370182 PMC6723295

[31] Mader S Rubach M Schaecke W Röger C Feldhoffer I Thalmeier EM. Healthy nutrition in Germany: a survey analysis of social causes, obesity and socioeconomic status. Public Health Nutr. (2020) 23:2109–23. 10.1017/S1368980019004877 32338236 PMC10200647

[32] Allen LH. Micronutrient research, programs, and policy: from meta-analyses to metabolomics. Adv Nutr. (2014) 5:344S−51S. 10.3945/an.113.005421 24829487 PMC4013192

[33] Pasricha SR Drakesmith H Black J Hipgrave D Biggs BA. Control of iron deficiency anemia in low- and middle-income countries. Blood. (2013) 121:2607–17. 10.1182/blood-2012-09-453522 23355536

[34] Majamanda J Maureen D Munkhondia TM Carrier J. The effectiveness of community-based nutrition education on the nutrition status of under-five children in developing countries. a systematic review. Malawi Med J. (2014) 26:115–8. 26167260 PMC4325345

[35] Habib A Kureishy S Soofi S Hussain I Rizvi A Ahmed I. Prevalence and risk factors for iron deficiency anemia among children under five and women of reproductive age in Pakistan: findings from the National Nutrition Survey 2018. Nutrients. (2023) 15:3361. 10.3390/nu15153361 37571298 PMC10421143

[36] Choedon T Brennan E Joe W Lelijveld N Huse O Zorbas C. Nutritional status of school-age children (5-19 years) in South Asia: a scoping review. Matern Child Nutr. (2024) 20:e13607. 10.1111/mcn.13607 38095279 PMC10981491

[37] Piper JD Mazhanga C Mwapaura M Mapako G Mapurisa I Mashedze T. The Sanitation Hygiene Infant Nutrition Efficacy (SHINE) Trial: protocol for school-age follow-up. Wellcome Open Res. (2023) 8:306. 10.12688/wellcomeopenres.19463.1 38031545 PMC10685067

[38] Medin AC Vik FN Helle C Helland SH Wills AK Osorio NG. Scaling up evidence-based digital early life nutrition interventions in a county setting: an implementation trial - protocol for Phase 2 of the *Nutrition Now* project. Front Public Health. (2024) 11:1326787. 10.3389/fpubh.2023.1326787 38264256 PMC10803599

[39] Yu Y Li H Hu NX Wu XH Huang XY Lin HT. Global burden and health inequality of nutritional deficiencies from 1990 to 2019. Front Nutr. (2024) 11:1470713. 10.3389/fnut.2024.1470713 39385781 PMC11461340

[40] GBD 2017 Diet Collaborators. Health effects of dietary risks in 195 countries, 1990-2017: a systematic analysis for the Global Burden of Disease Study 2017. Lancet. (2019) 393:1958–72. 30954305 10.1016/S0140-6736(19)30041-8 PMC6899507

[41] Watts N Amann M Arnell N Ayeb-Karlsson S Belesova K Berry H. The 2018 report of the Lancet Countdown on health and climate change: shaping the health of nations for centuries to come. Lancet. (2018) 392:2479–514. 10.1016/S0140-6736(18)32594-7 30503045 PMC7616804

