# Supplementary figures and images for: Trends and cross-country inequality in the global burden of nutritional deficiencies in children, with projections to 2035: results from the Global Burden of Disease study 2021
Source: Front Nutr. 2025 Jul 29;12:1615593. doi: 10.3389/fnut.2025.1615593 (PMC12340229; doi:10.3389/fnut.2025.1615593)

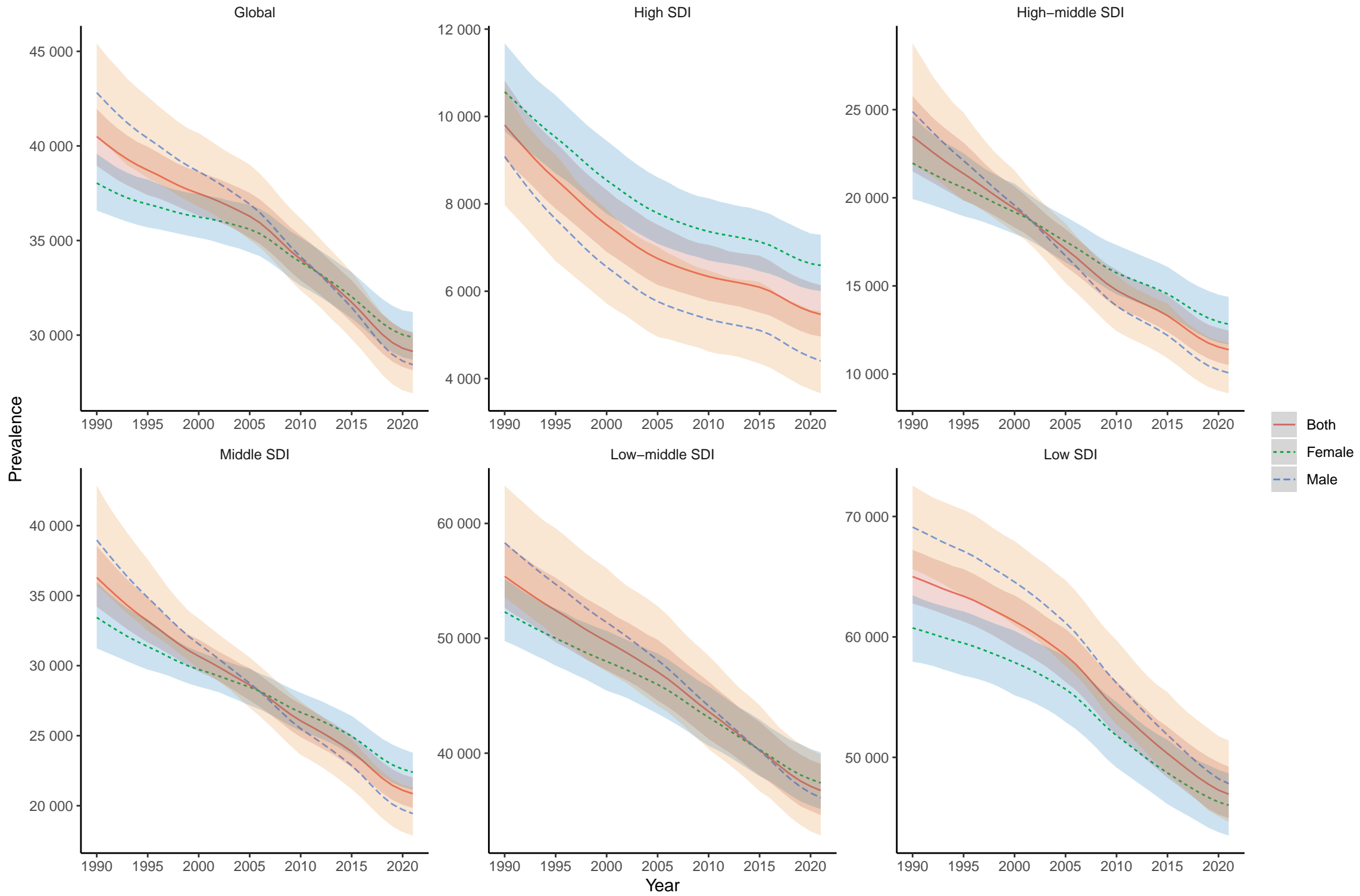

Supplement: Supplementary Figure S1 — Temporal trend of age standardized prevalence rate of of nutritional deficiencies, globally and by sociodemographic index, from 1990 to 2021. [file Image_1.pdf]

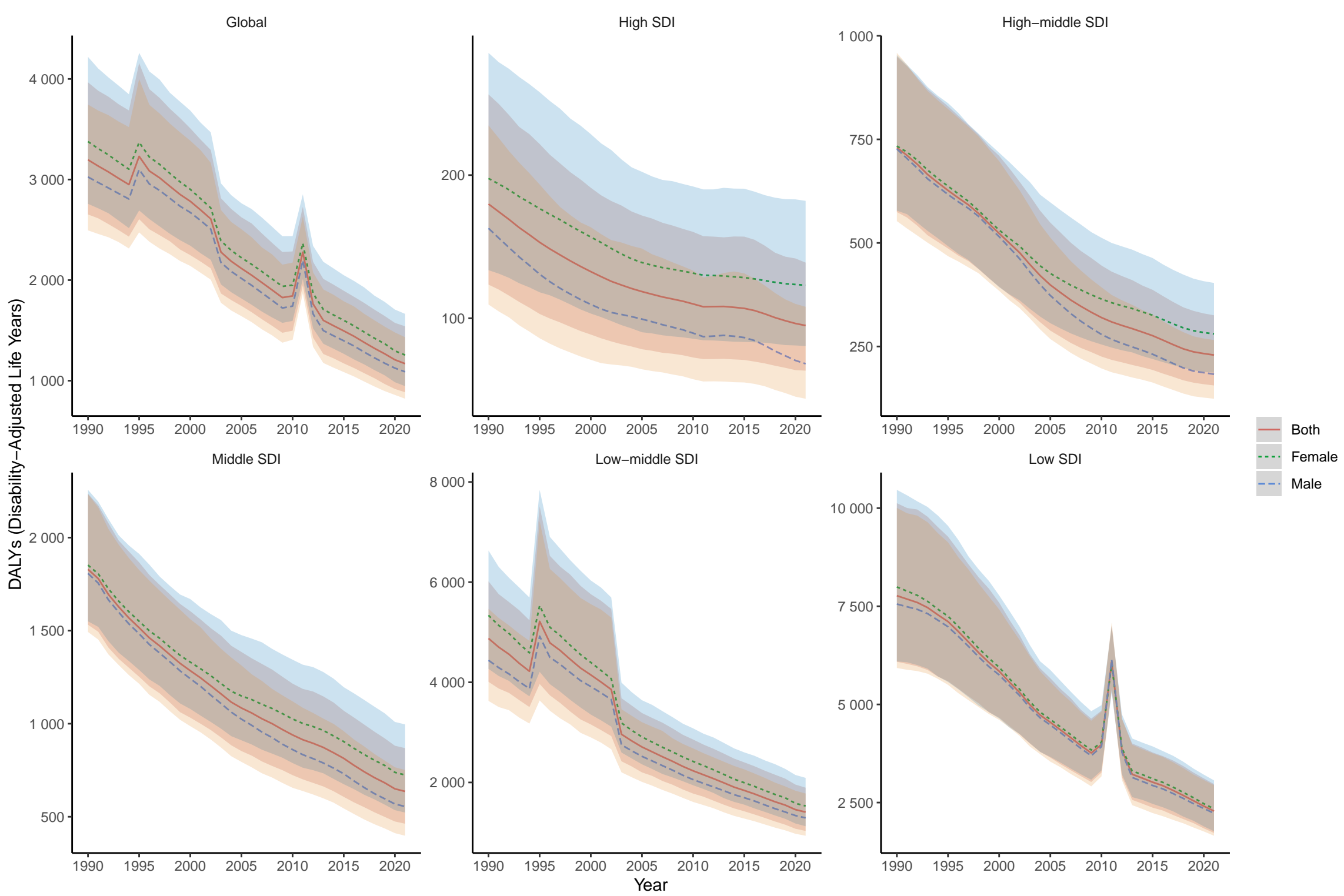

Supplement: Supplementary Figure S2 — Temporal trend of age standardized DALYs rate of of nutritional deficiencies, globally and by sociodemographic index, from 1990 to 2021. [file Image_2.pdf]

Deaths

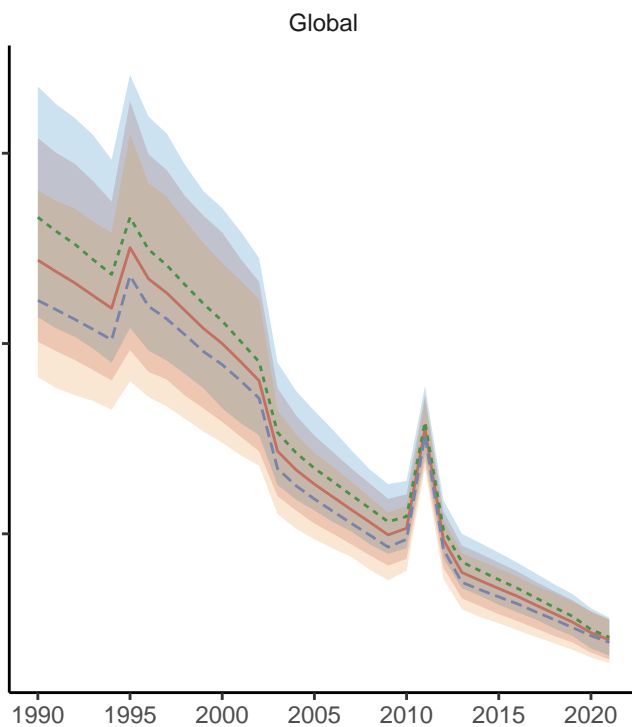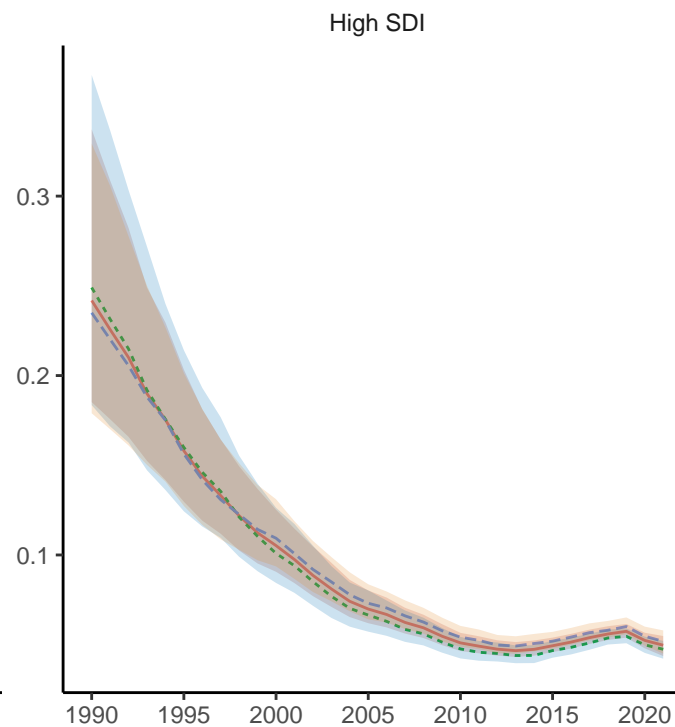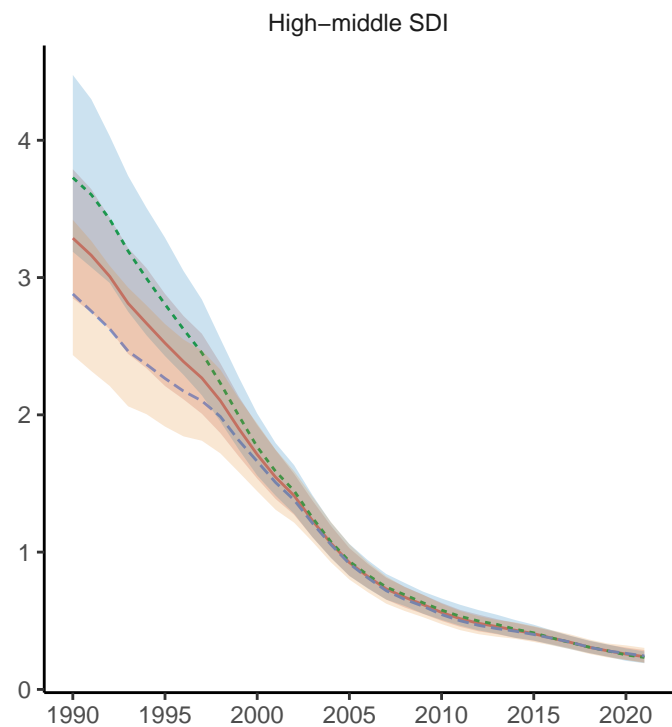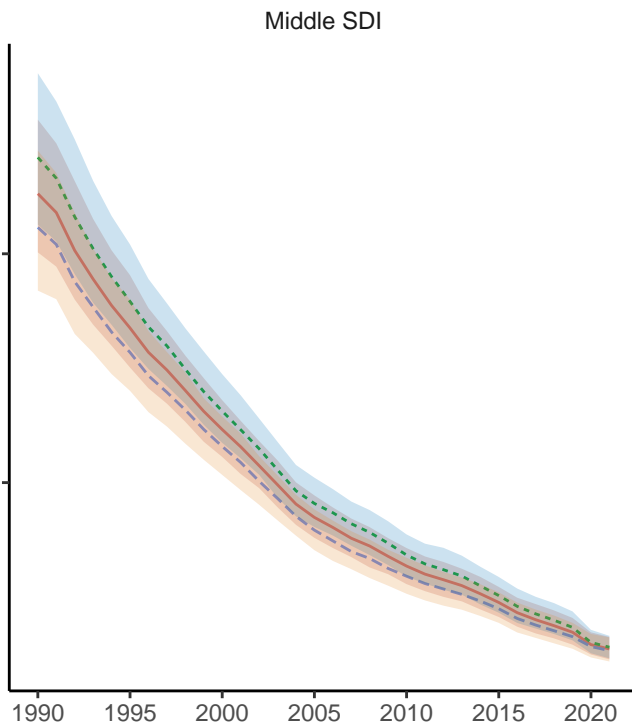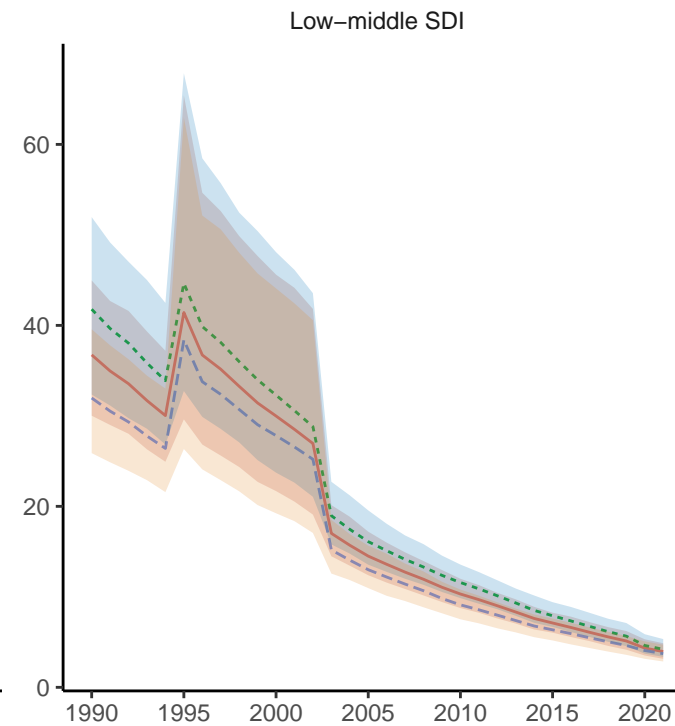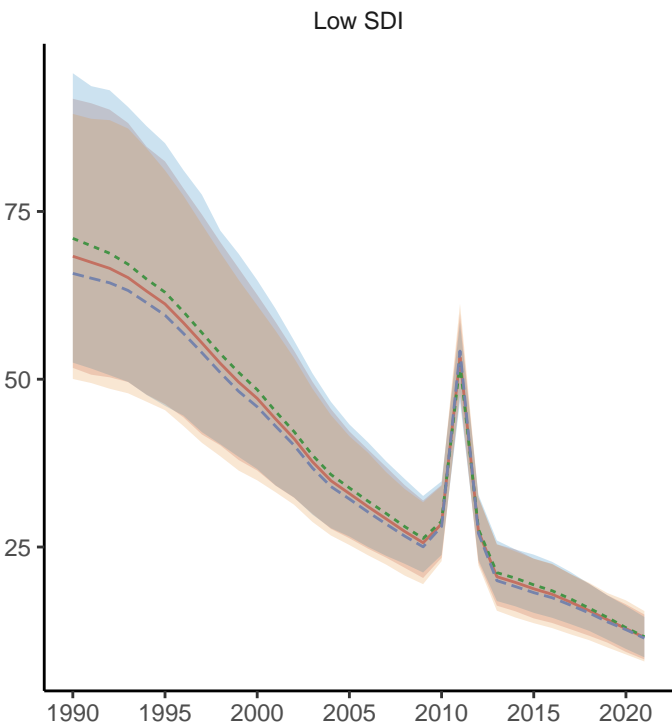

Both  
Female  
Male

Supplement: Supplementary Figure S3 — Temporal trend of age standardized deaths rate of of nutritional deficiencies, globally and by sociodemographic index, from 1990 to 2021. [file Image_3.pdf]

A

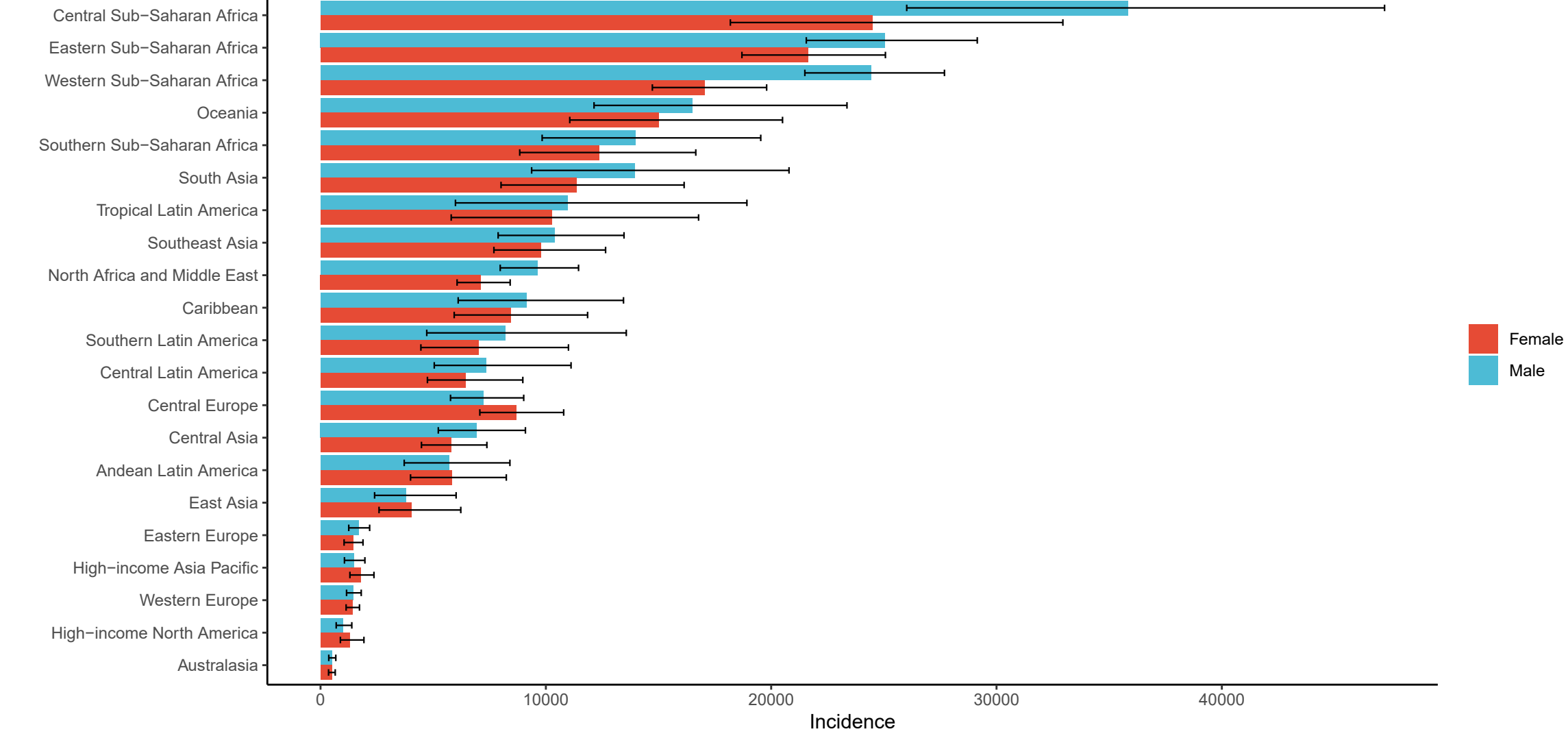

B

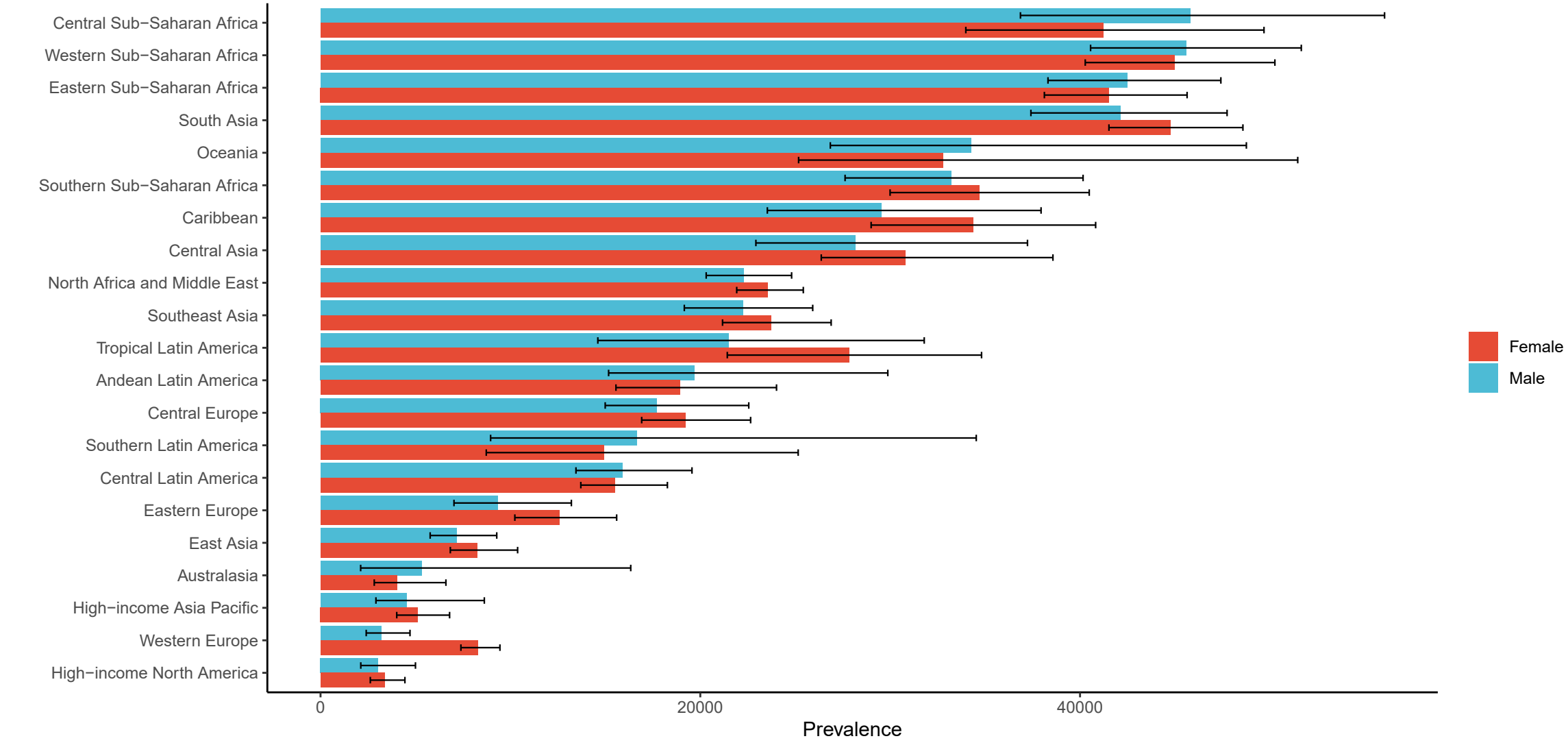

C

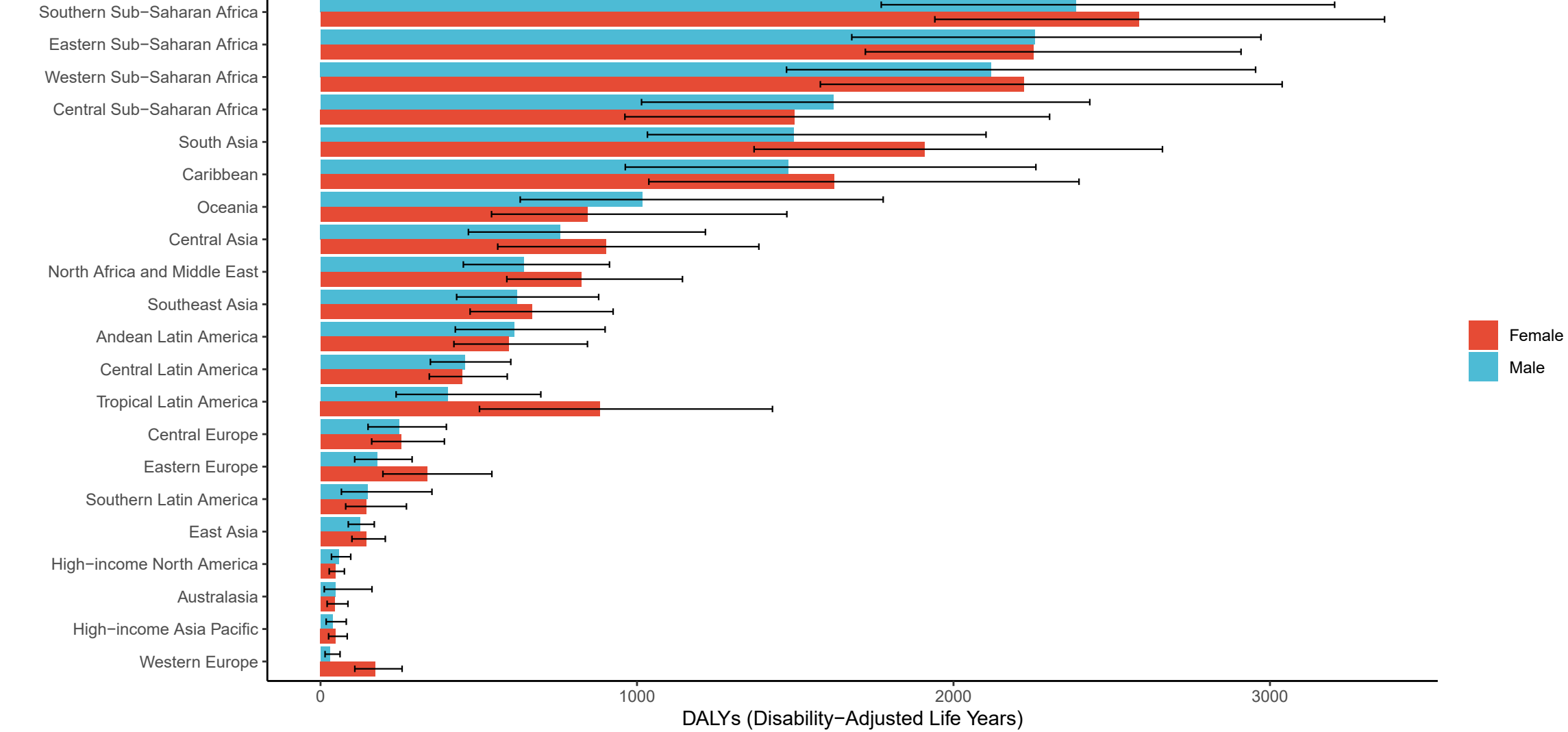

D

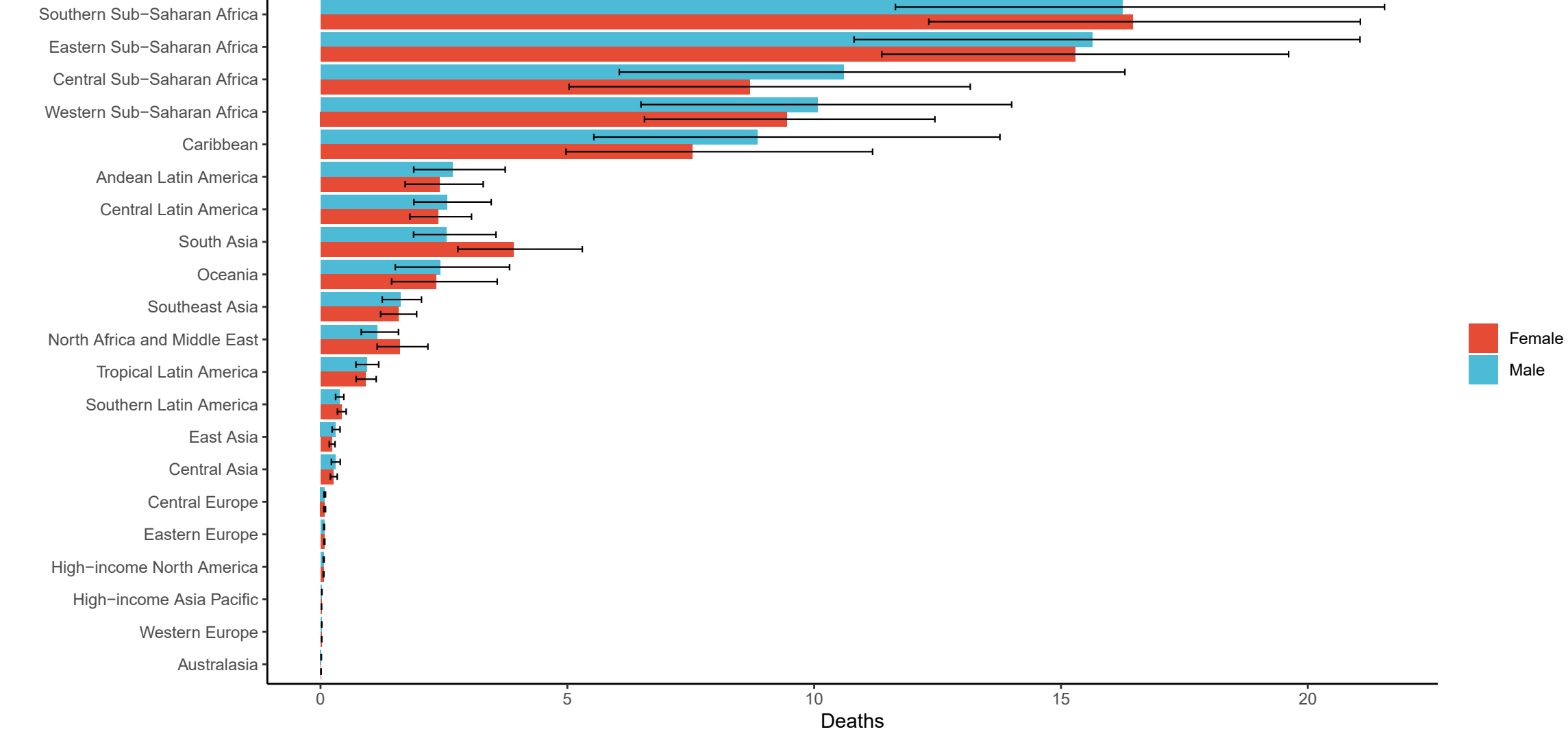

Supplement: Supplementary Figure S4 — Sex differences in (A) incidence; (B) prevalence; (C) DALYs; (D) deaths, of nutritional deficiencies for 21 regions in 2021. [file Image_4.pdf]

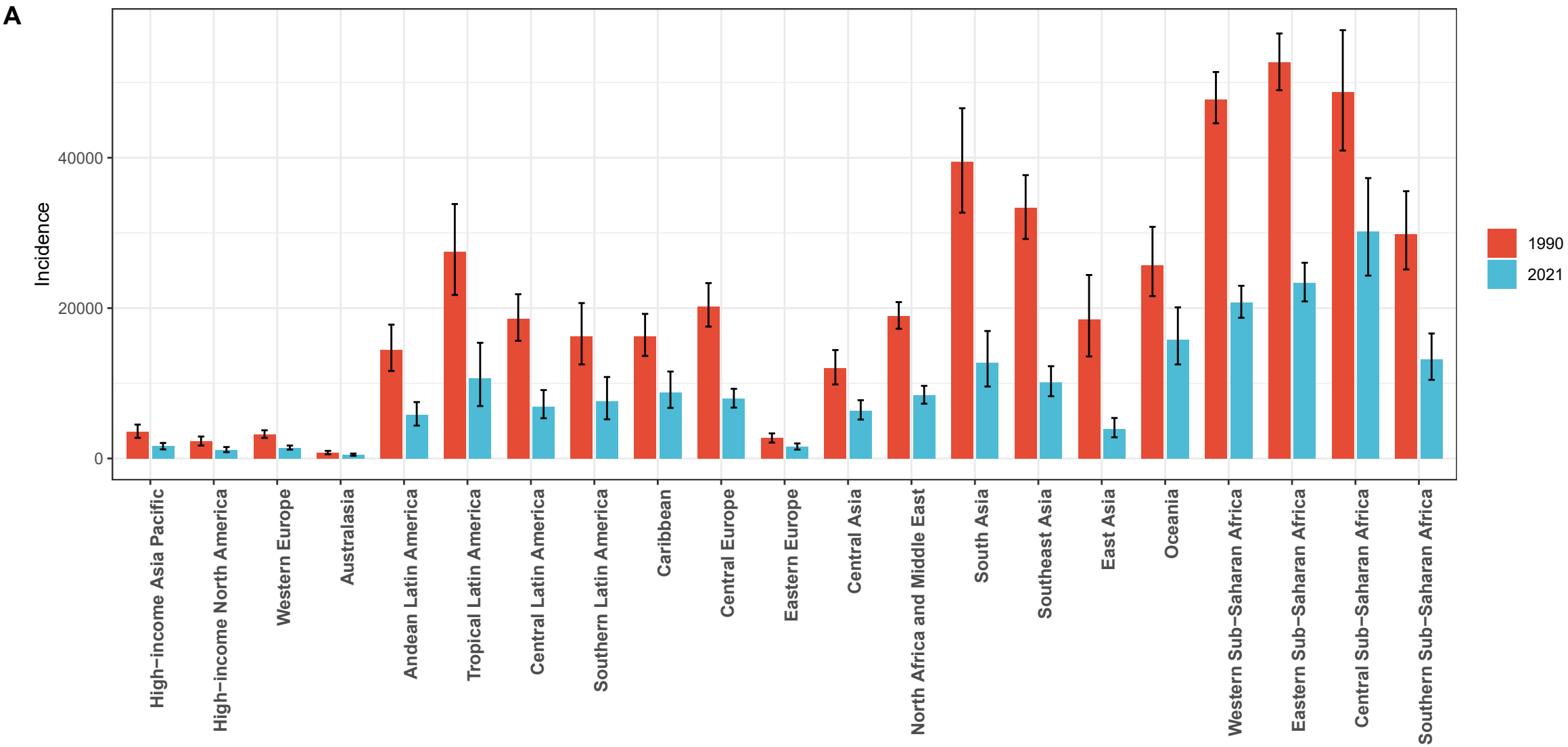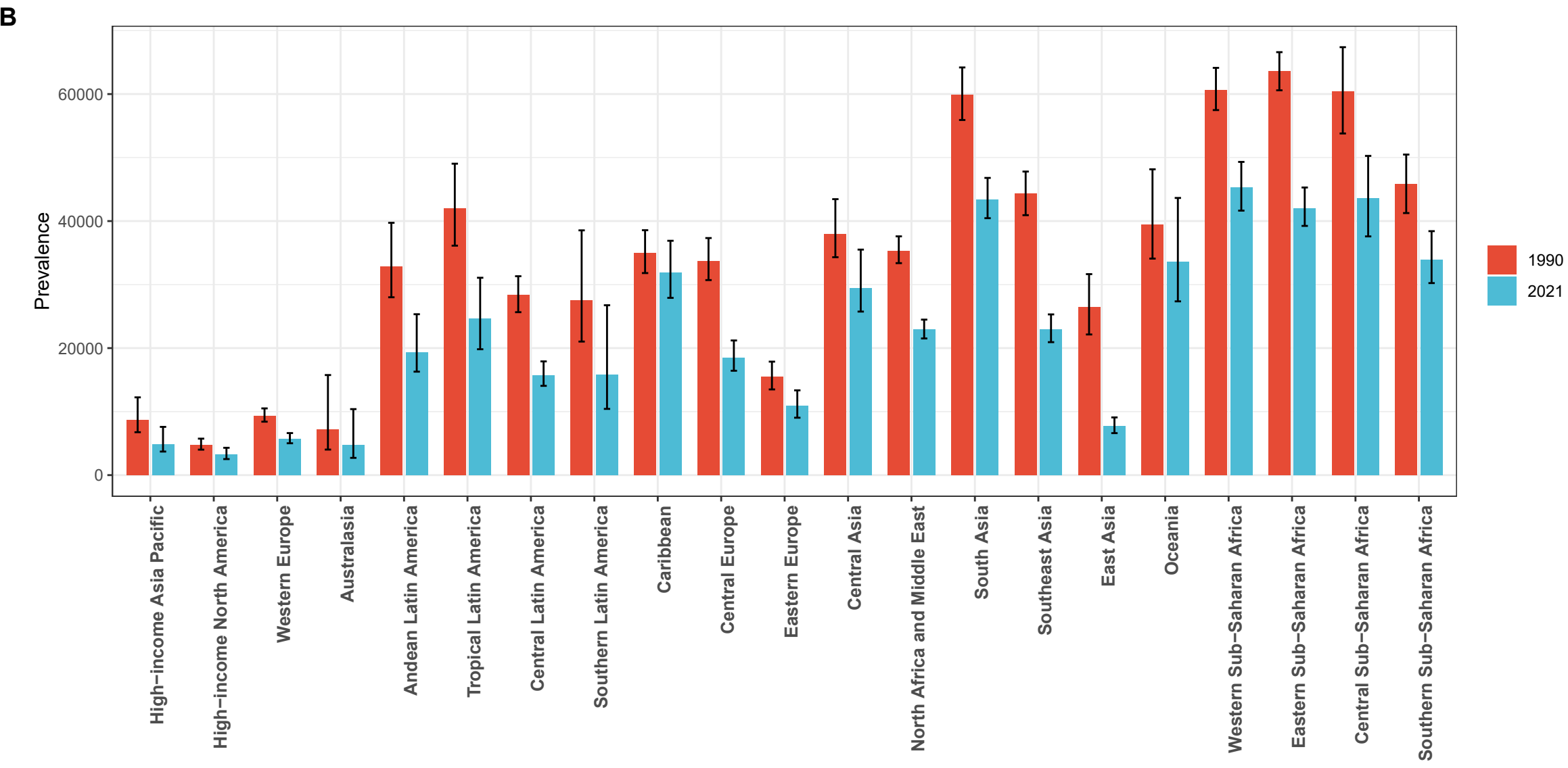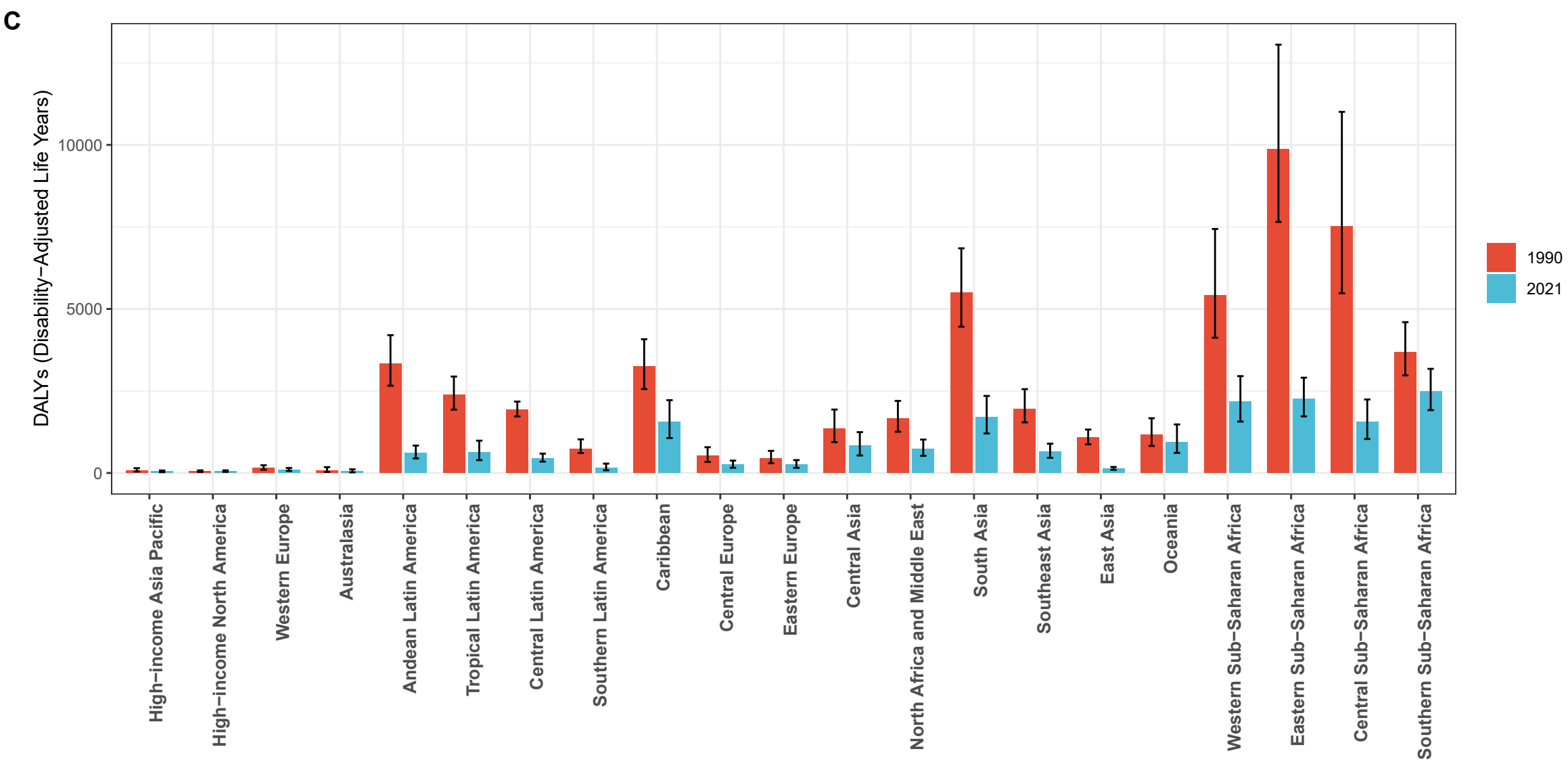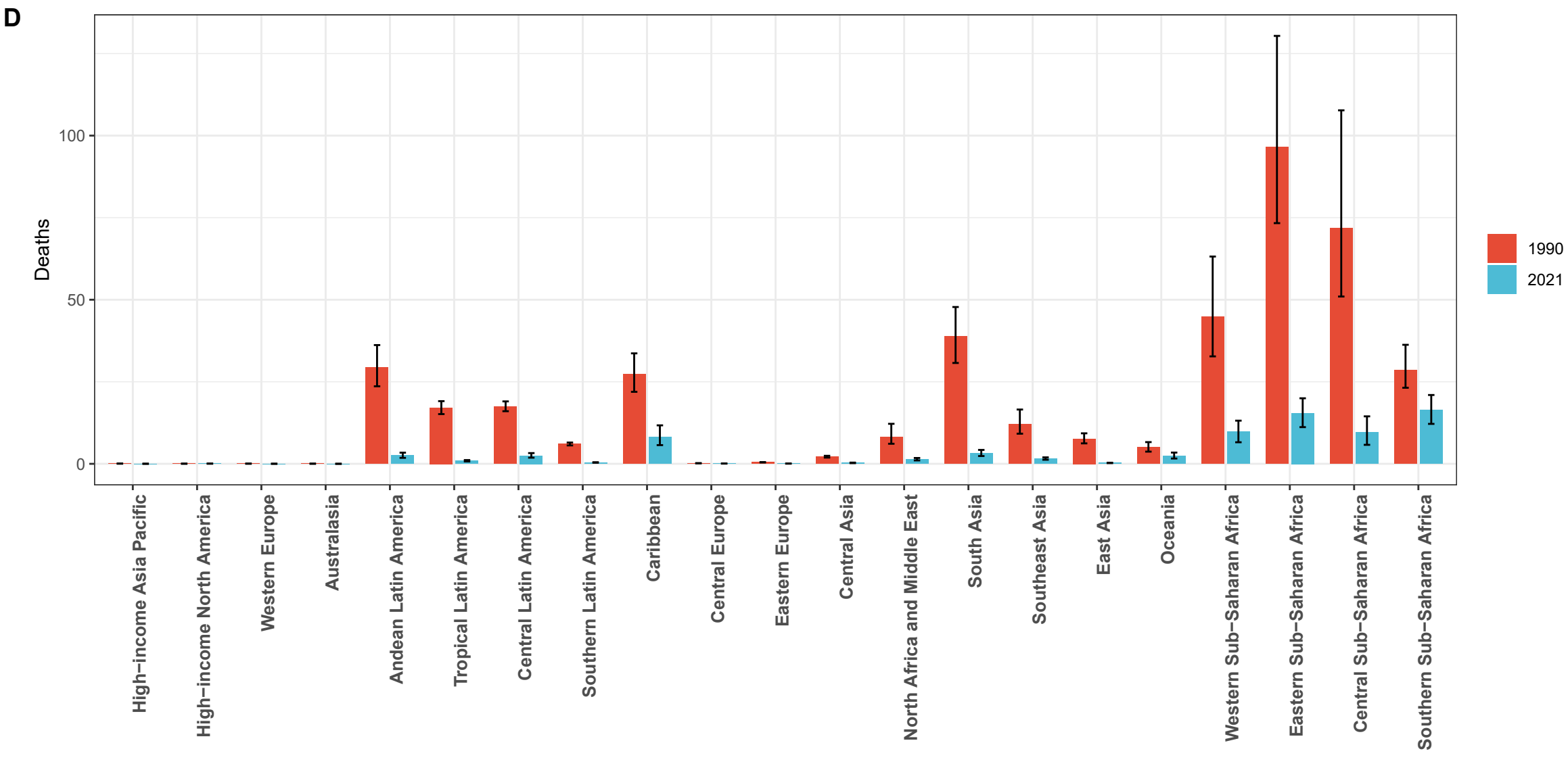

Supplement: Supplementary Figure S5 — Year differences in (A) incidence; (B) prevalence; (C) DALYs; (D) deaths, of nutritional deficiencies for 21 regions in 1990 and 2021. [file Image_5.pdf]

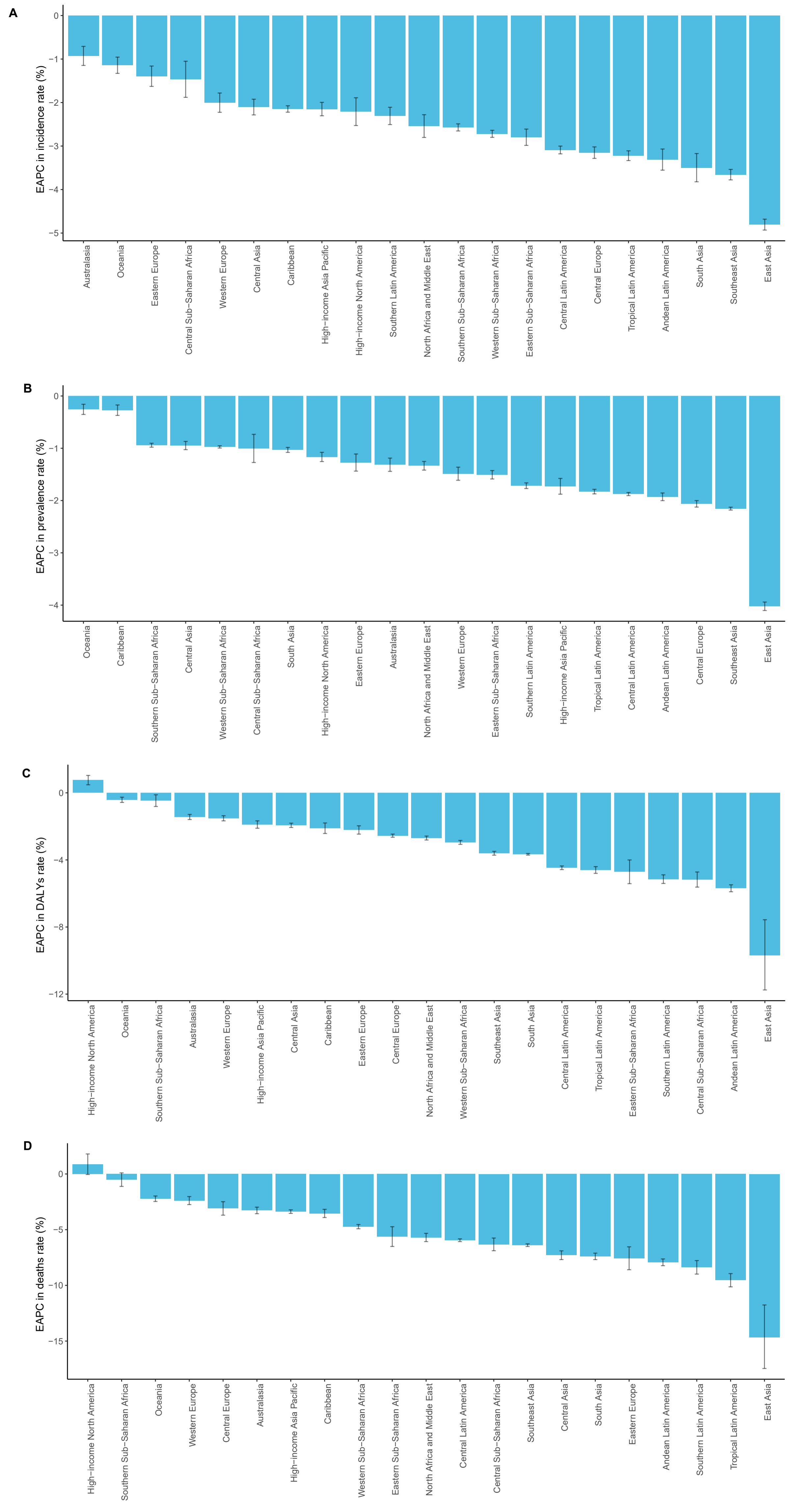

Supplement: Supplementary Figure S6 — EAPC of (A) incidence; (B) prevalence; (C) DALYs; (D) deaths, of nutritional deficiencies for 21 regions from 1990 to 2021. [file Image_6.pdf]

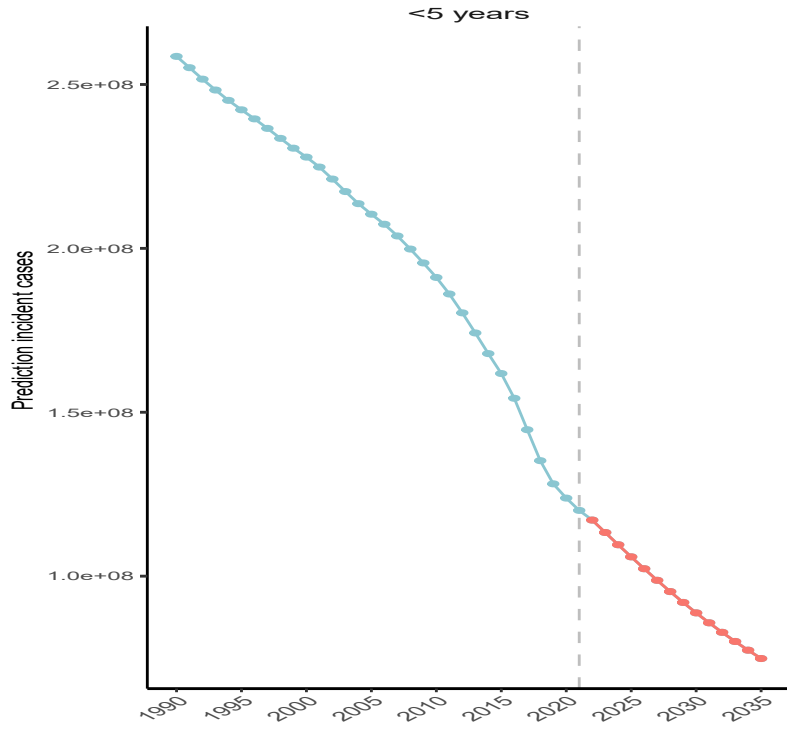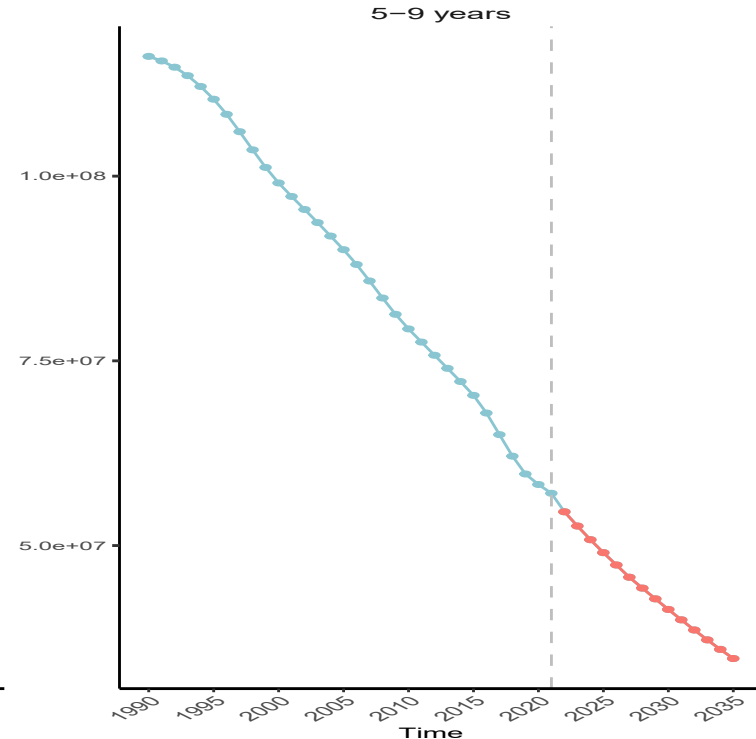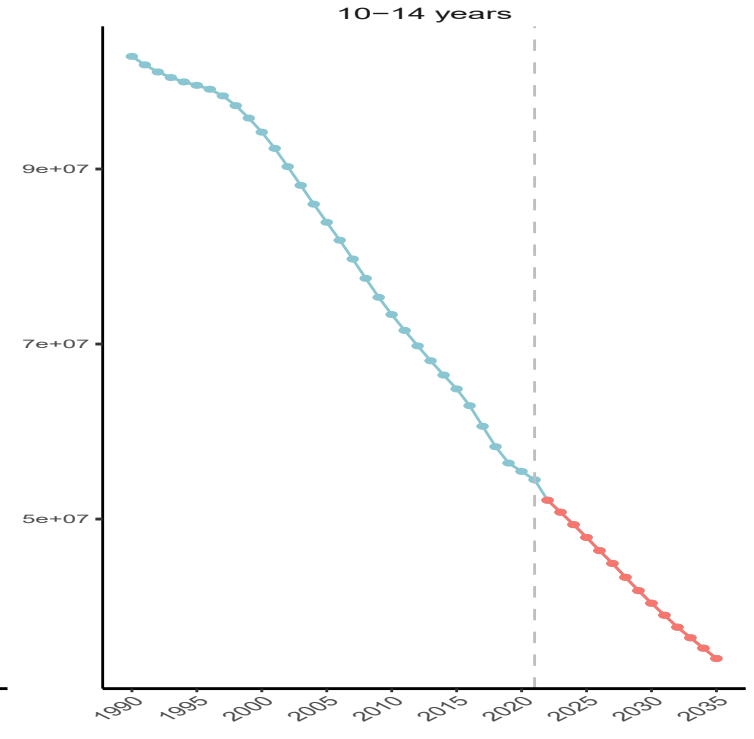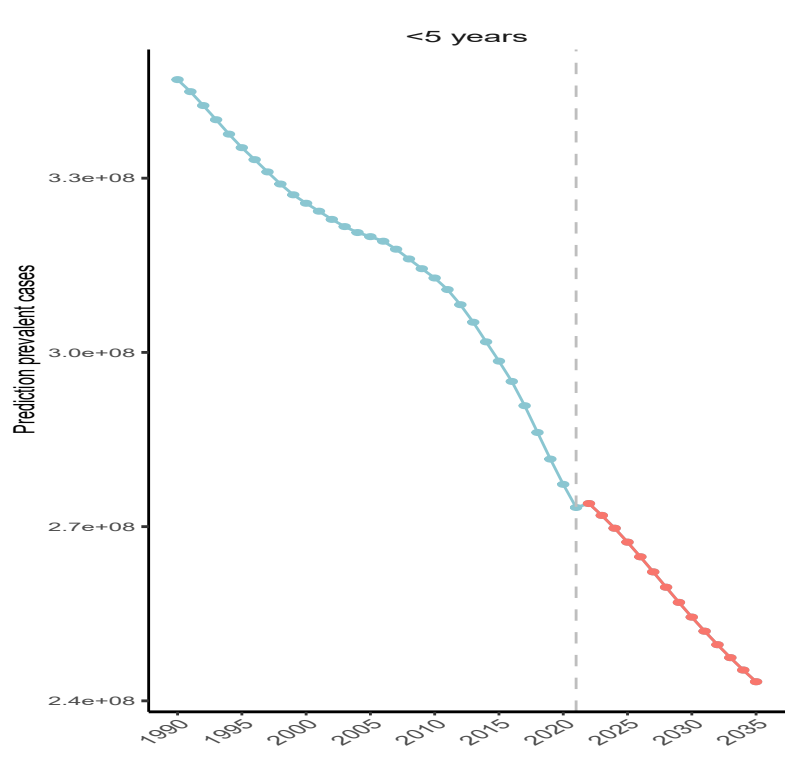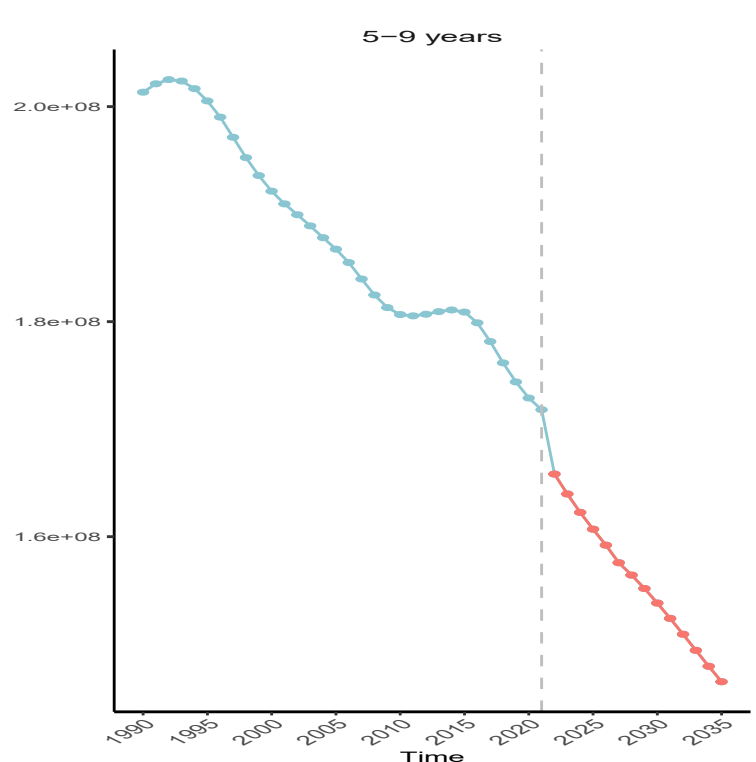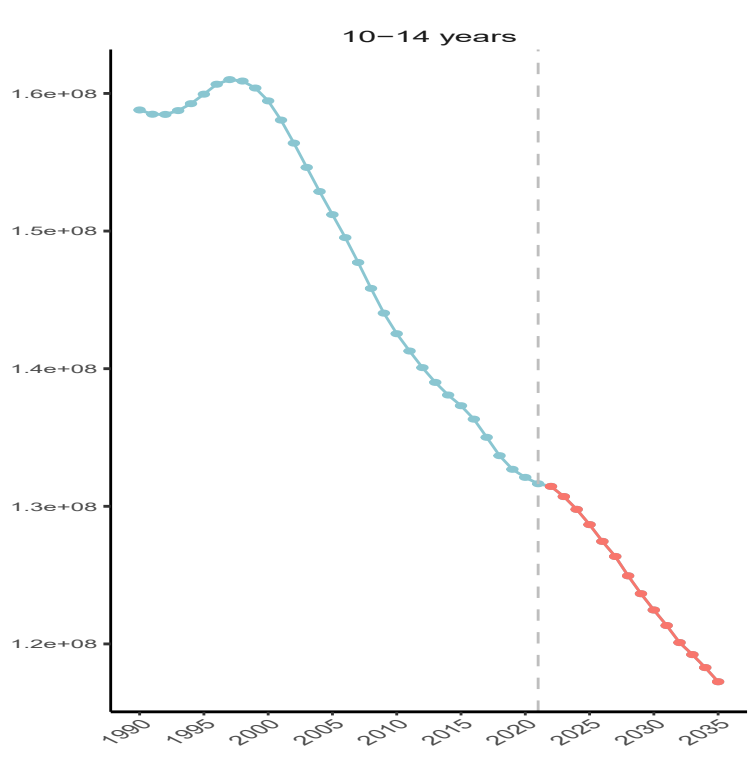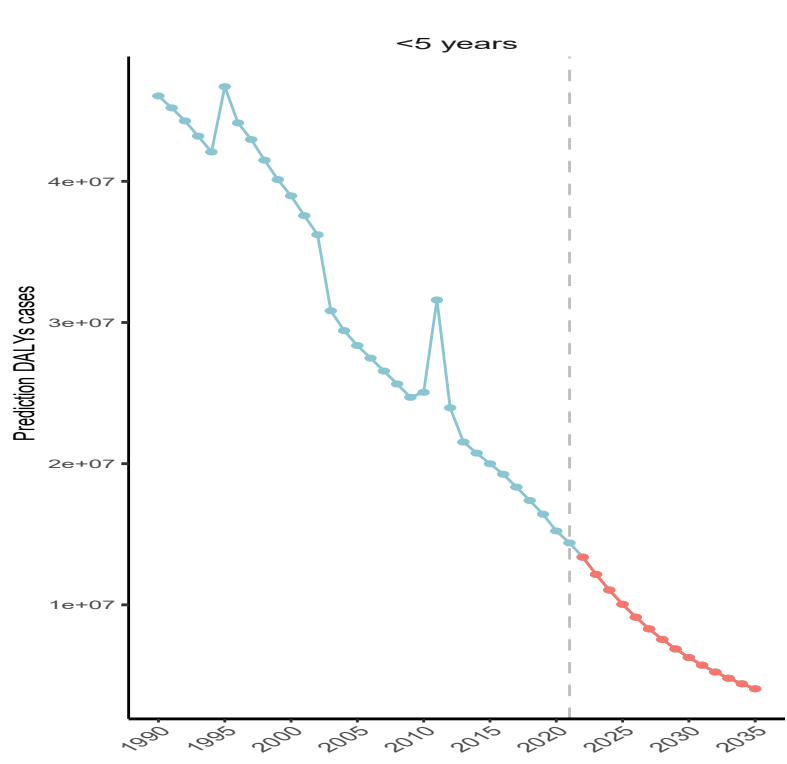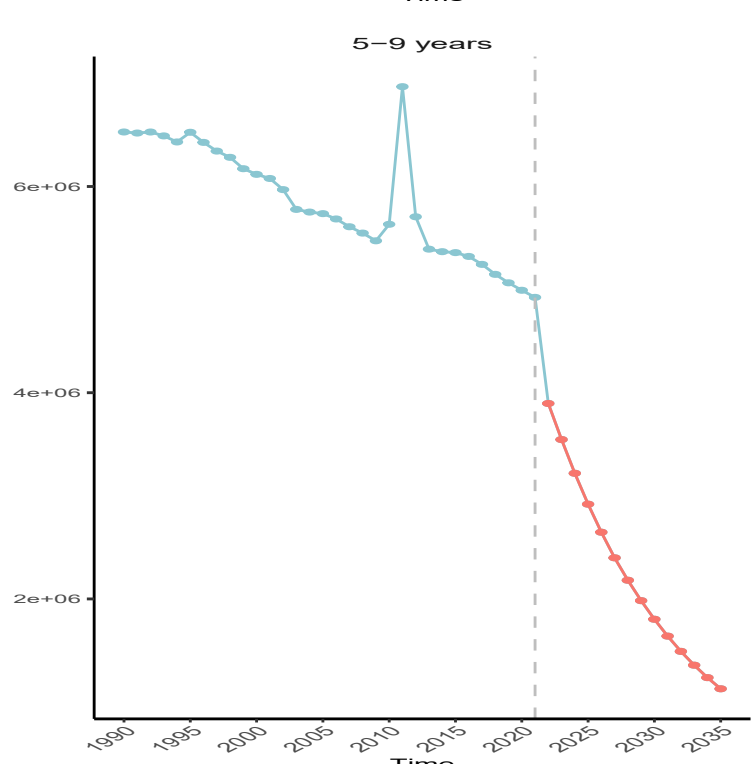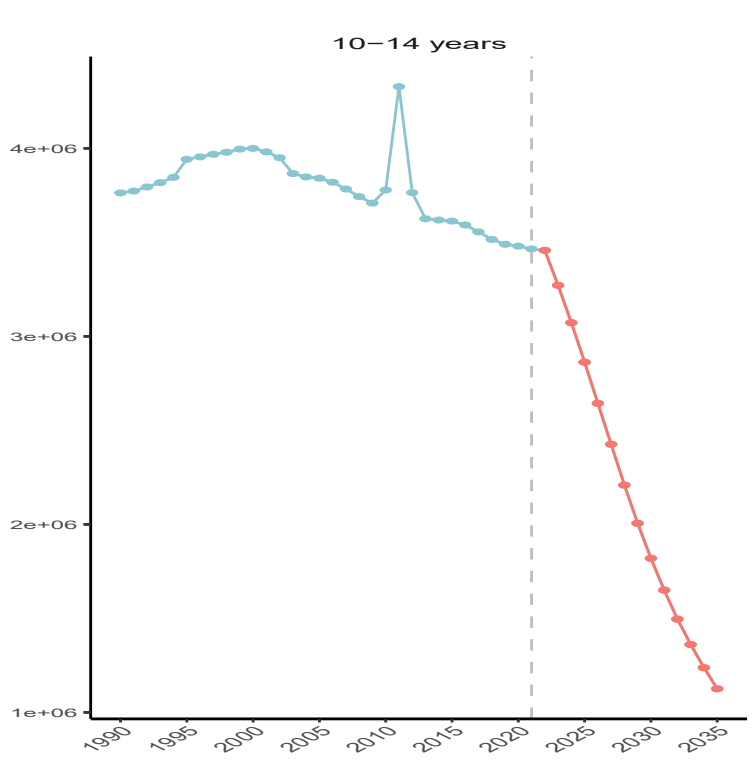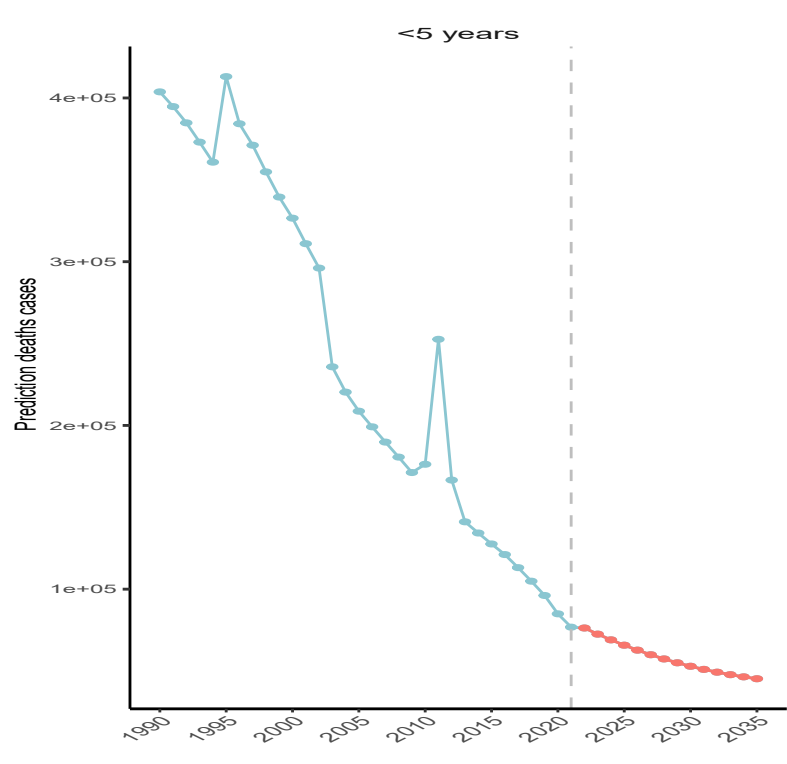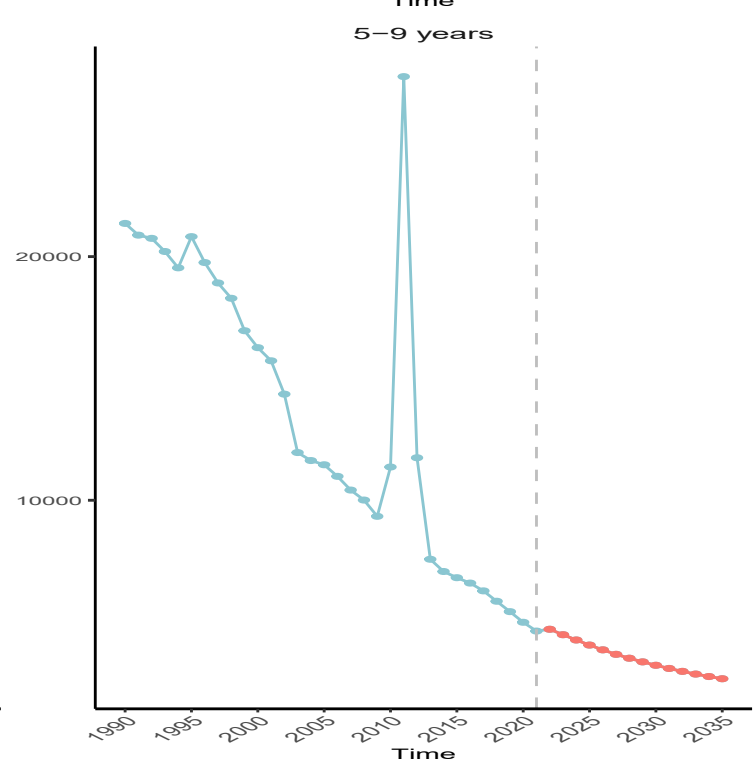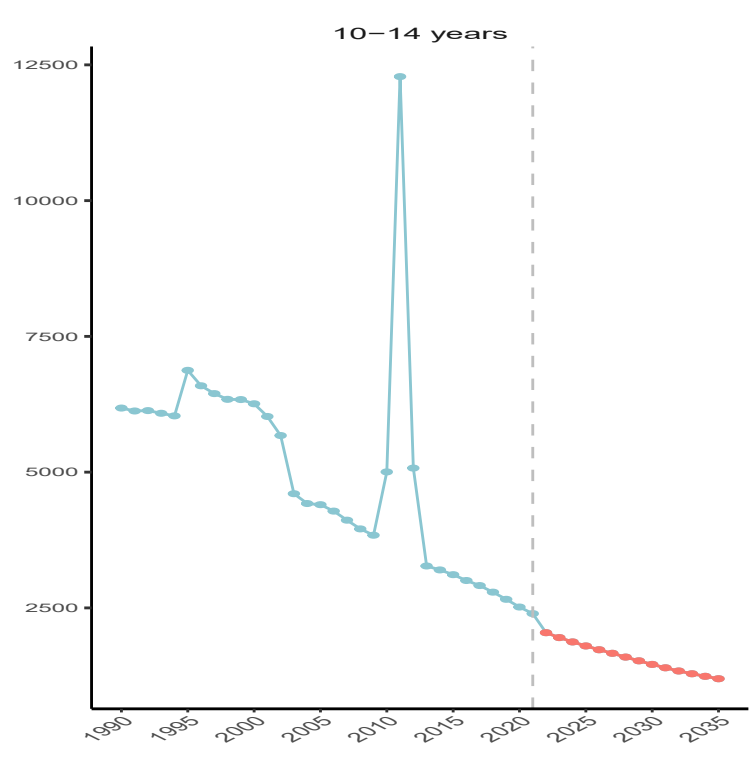

Supplement: Supplementary Figure S7 — Predictive analysis for age groups cases of nutritional deficiencies globally from 2022 to 2035. [file Image_7.pdf]

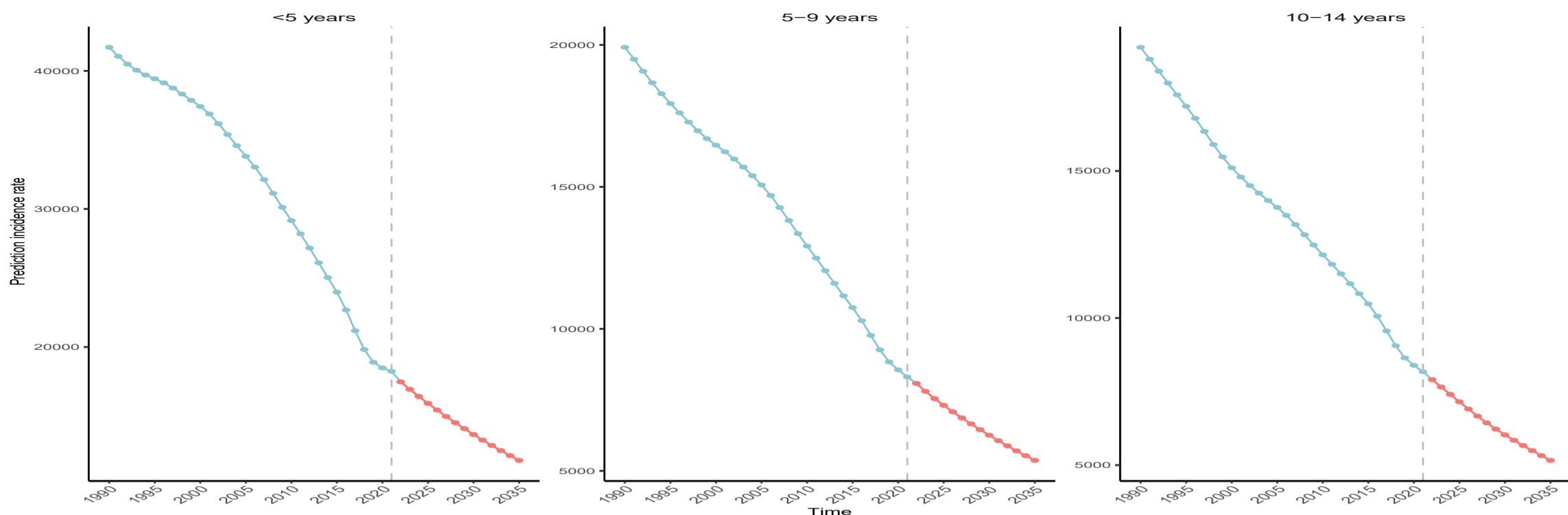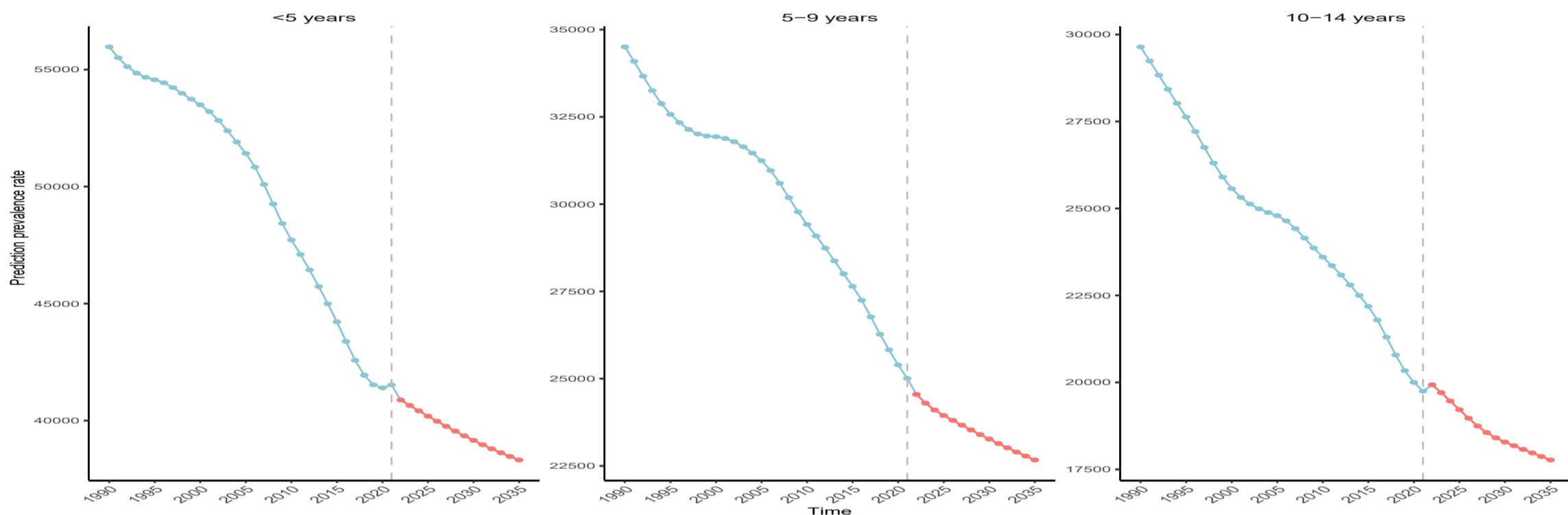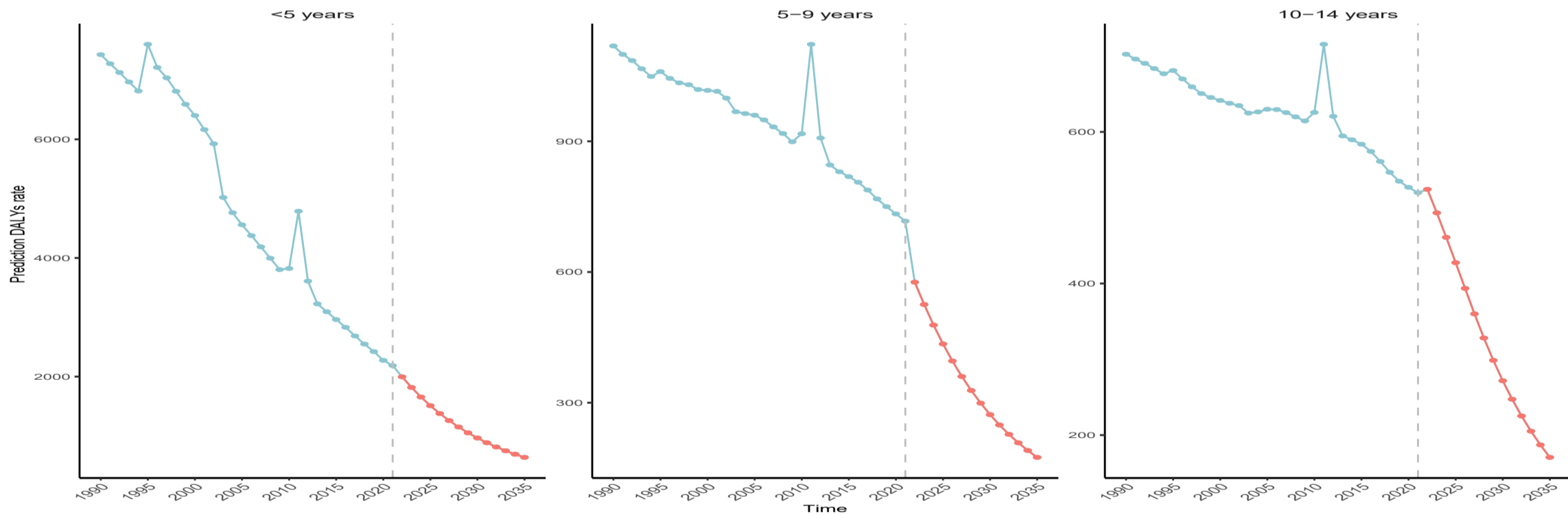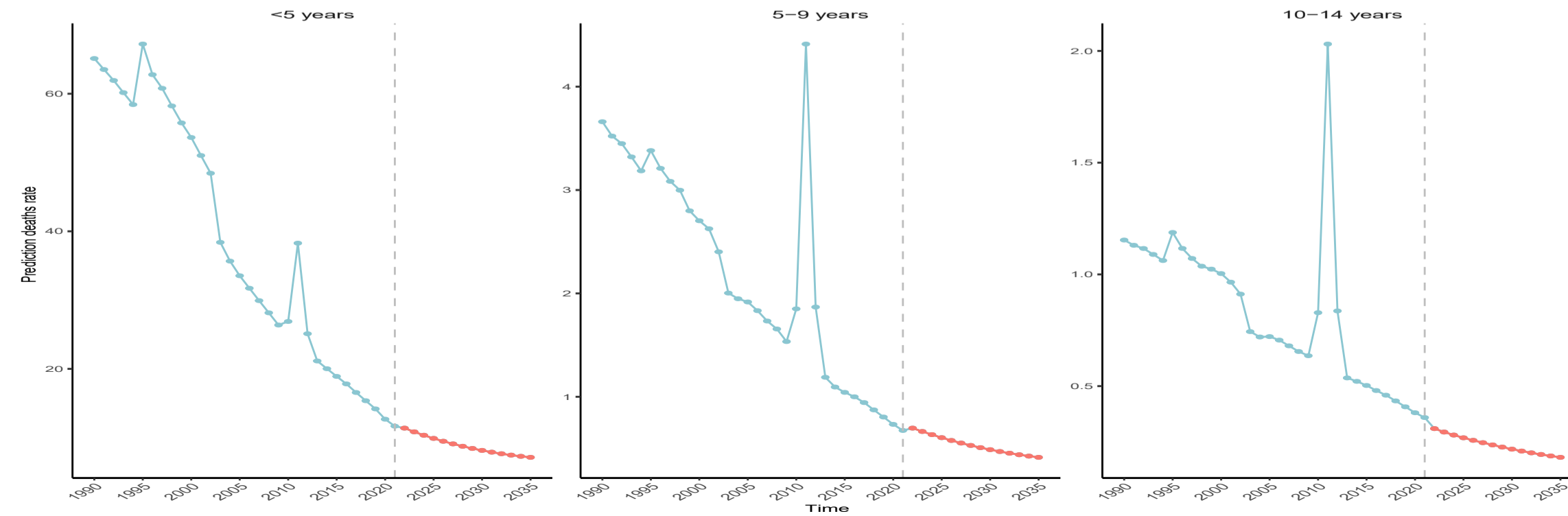

Supplement: Supplementary Figure S8 — Predictive analysis for age groups rates of nutritional deficiencies globally from 2022 to 2035. [file Image_8.pdf]
