# Supplementary material for: Trends and cross-country inequality in the global burden of nutritional deficiencies in children, with projections to 2035: results from the Global Burden of Disease study 2021
Source: Front Nutr. 2025 Jul 29;12:1615593. doi: 10.3389/fnut.2025.1615593 (PMC12340229; doi:10.3389/fnut.2025.1615593)
Supplement: Supplementary file 9 [file Table_1.docx]

**Table S1** Age standardized prevalence rate (ASPR) of nutritional deficiencies in 1990 and 2021, and estimated annual percentage change (EAPC) from 1990 to 2021 at the global and regional level.

| Group | **1990** | | **2021** | | **1990-2021** |
| --- | --- | --- | --- | --- | --- |
|  | **Prevalent cases, 000s**  **(95% CI)** | **ASPRs**  **per 100 000**  **(95% CI)** | **Prevalent cases, 000s**  **(95% CI)** | **ASPRs**  **per 100 000**  **(95% CI)** | **EAPC, %,**  **(95% CI)** |
| Global | 707118.754(680352.589,732523.134) | 40493.091(38948.234,41960.073) | 576745.248(556502.814,597031.183) | 29138.407(28142.174,30137.099) | -1.045(-1.118,-0.973) |
| SDI |  |  |  |  |  |
| High | 17913.732(16465.12,19791.115) | 9803.731(9018.566,10822.204) | 9072.673(8223.009,10197.178) | 5469.56(4964.148,6137.379) | -1.764(-1.887,-1.641) |
| High-middle | 63684.66(58267.984,69987.655) | 23477.803(21500.369,25778.013) | 25209.998(23215.816,27675.503) | 11382.002(10505.516,12462.942) | -2.392(-2.450,-2.334) |
| Middle | 209075.901(196990.871,222083.889) | 36293.594(34202.815,38544.07) | 114570.856(108720.098,121222.993) | 20854.273(19827.46,22017.071) | -1.726(-1.779,-1.673) |
| Low-middle | 264076.151(251243.31,277696.115) | 55391.185(52638.727,58314.894) | 210241.874(197737.744,223853.713) | 36747.312(34603.472,39076) | -1.330(-1.381,-1.280) |
| Low | 151823.267(146910.309,156645.775) | 65009.248(62774.738,67217.758) | 217239.171(208120.354,227652.419) | 46946.567(44953.006,49227.059) | -1.126(-1.196,-1.055) |
| Regions |  |  |  |  |  |
| Andean Latin America | 4893.689(4178.893,5914.273) | 32831.26(28004.899,39732.595) | 3462.052(2914.323,4548.589) | 19326.239(16291.633,25337.557) | -1.929(-2.002,-1.856) |
| Australasia | 323.021(180.415,703.083) | 7227.522(4024.867,15756.987) | 253.446(147.864,558.605) | 4693.401(2717.343,10394.755) | -1.315(-1.442,-1.188) |
| Caribbean | 4009.169(3657.192,4421.567) | 34920.979(31814.446,38563.485) | 3637.102(3176.301,4214.701) | 31912.522(27909.691,36902.019) | -0.272(-0.372,-0.172) |
| Central Asia | 9667.724(8757.091,11010.732) | 37995.014(34311.035,43452.61) | 8223.763(7198.093,9912.784) | 29438.507(25751.998,35508.925) | -0.948(-1.026,-0.869) |
| Central Europe | 9686.445(8810.766,10763.113) | 33675.762(30711.642,37328.095) | 3174.683(2825.011,3653.25) | 18448.56(16436.106,21204.339) | -2.063(-2.125,-2.001) |
| Central Latin America | 18355.074(16596.45,20231.264) | 28395.102(25656.824,31319.471) | 9643.64(8600.512,11060.207) | 15695.372(14056.835,17908.223) | -1.876(-1.905,-1.846) |
| Central Sub-Saharan Africa | 15656.308(14048.539,17332.306) | 60428.31(53792.602,67381.904) | 25660.858(22178.924,29584.962) | 43548.735(37591.899,50264.45) | -1.003(-1.272,-0.734) |
| East Asia | 87342.586(73042.263,104373.317) | 26478.267(22147.883,31647.274) | 19880.406(17030.397,23715.005) | 7668.424(6614.564,9085.849) | -4.022(-4.103,-3.941) |
| Eastern Europe | 7856.765(6819.822,9061.298) | 15520.195(13495.809,17869.887) | 3577.086(2944.962,4403.337) | 10903.303(9039.44,13350.767) | -1.273(-1.437,-1.110) |
| Eastern Sub-Saharan Africa | 58785.997(56194.389,61395.877) | 63590.696(60591.736,66600.234) | 75281.514(70362.109,81103.142) | 42010.88(39243.17,45288.996) | -1.507(-1.587,-1.426) |
| High-income Asia Pacific | 2842.269(2201.284,4014.39) | 8698.006(6757.854,12246.157) | 990.194(762.405,1571.396) | 4812.735(3711.016,7598.24) | -1.728(-1.878,-1.577) |
| High-income North America | 2934.369(2474.65,3541.188) | 4759.207(4013.146,5743.306) | 2048.262(1615.081,2745.139) | 3203.87(2527.767,4306.092) | -1.166(-1.254,-1.078) |
| North Africa and Middle East | 50156.943(47483.305,53410.077) | 35282.485(33378.966,37596.857) | 41340.159(38809.965,44242.472) | 22900.114(21517.19,24484.687) | -1.335(-1.418,-1.253) |
| Oceania | 1079.34(935.956,1307.631) | 39484.21(34092.4,48152.743) | 1755.588(1441.413,2264.57) | 33545.722(27359.957,43642.682) | -0.256(-0.353,-0.159) |
| South Asia | 261561.229(244379.779,279872.699) | 59920.332(55917.869,64190.154) | 214429.828(199383.638,231946.559) | 43395.245(40459.146,46801.576) | -1.032(-1.079,-0.984) |
| Southeast Asia | 75163.378(69331.156,81130.589) | 44328.129(40933.353,47799.623) | 38642.648(35144.408,42694.629) | 22963.922(20935.158,25309.908) | -2.155(-2.182,-2.127) |
| Southern Latin America | 4082.283(3120.066,5726.033) | 27496.965(21029.736,38541.191) | 2153.006(1428.501,3680.168) | 15798.159(10428.798,26749.9) | -1.716(-1.770,-1.662) |
| Southern Sub-Saharan Africa | 9528.161(8592.948,10485.042) | 45815.523(41263.43,50474.498) | 8091.643(7192.794,9172.766) | 33946.387(30236.908,38413.773) | -0.942(-0.979,-0.904) |
| Tropical Latin America | 22317.991(19129.64,26159.447) | 41990.458(36131.899,49038.033) | 12294.445(9894.164,15541.476) | 24601.42(19821.328,31078.043) | -1.829(-1.872,-1.785) |
| Western Europe | 6433.558(5808.281,7256.98) | 9319.267(8416.753,10507.425) | 3710.141(3275.689,4332.062) | 5677.2(5019.038,6621.008) | -1.487(-1.612,-1.362) |
| Western Sub-Saharan Africa | 54442.455(51775.658,57389.852) | 60631.333(57476.851,64127.857) | 98494.783(90782.889,107016.371) | 45290.905(41647.407,49318.156) | -0.974(-0.996,-0.953) |

ASPR = age standardized prevalence rate; EAPC = estimated annual percentage change; SDI = socio-demographic index; 95% CI = 95% confidence interval.
