# Supplementary material for: Trends and cross-country inequality in the global burden of nutritional deficiencies in children, with projections to 2035: results from the Global Burden of Disease study 2021
Source: Front Nutr. 2025 Jul 29;12:1615593. doi: 10.3389/fnut.2025.1615593 (PMC12340229; doi:10.3389/fnut.2025.1615593)
Supplement: Supplementary file 10 [file Table_2.docx]

**Table S2** Age standardized DALYs rate (ASDR) of nutritional deficiencies in 1990 and 2021, and estimated annual percentage change (EAPC) from 1990 to 2021 at the global and regional level.

| **Group** | **1990** | | **2021** | | **1990-2021** |
| --- | --- | --- | --- | --- | --- |
|  | **DALYs cases, 000s**  **(95% CI)** | **ASDRs**  **per 100 000**  **(95% CI)** | **DALYs cases, 000s**  **(95% CI)** | **ASDRs**  **per 100 000**  **(95% CI)** | **EAPC, %,**  **(95% CI)** |
| Global | 56338.352(46785.994,69879.472) | 3196.189(2652.839,3966.312) | 22777.008(17186.853,30100.109) | 1169.192(885.2,1540.544) | -3.380(-3.668,-3.091) |
| SDI |  |  |  |  |  |
| High | 327.296(224.441,467.265) | 179.801(123.499,256.388) | 159.296(106.462,233.103) | 94.916(63.394,138.867) | -1.900(-2.054,-1.746) |
| High-middle | 1963.396(1541.74,2556.759) | 730.313(574.506,949.528) | 506.848(343.541,721.05) | 229.384(155.944,325.345) | -3.968(-4.091,-3.845) |
| Middle | 10505.695(8793.728,12844.634) | 1829.273(1531.704,2235.872) | 3448.974(2495.876,4728.188) | 636.161(462.51,868.794) | -3.231(-3.291,-3.170) |
| Low-middle | 23842.493(19636.88,29367.733) | 4876.055(4009.572,6015.681) | 7975.534(5824.137,10762.26) | 1408.291(1031.841,1895.336) | -4.347(-4.686,-4.006) |
| Low | 19668.678(15427.284,25615.585) | 7770.082(6086.36,10126.389) | 10671.191(8081.032,13862.355) | 2283.981(1727.512,2970.08) | -3.920(-4.301,-3.538) |
| Regions |  |  |  |  |  |
| Andean Latin America | 502.331(400.251,631.13) | 3340.65(2660.128,4200.76) | 107.366(78.665,149.033) | 603.053(442.399,835.749) | -5.691(-5.898,-5.483) |
| Australasia | 3.226(1.427,7.985) | 71.649(31.645,177.561) | 2.514(1.081,6.157) | 45.515(19.371,111.58) | -1.440(-1.591,-1.288) |
| Caribbean | 380.111(299.594,476.31) | 3248.451(2556.894,4076.064) | 174.426(119.429,250.524) | 1549.46(1064.62,2219.73) | -2.111(-2.424,-1.797) |
| Central Asia | 349.083(243.282,495.131) | 1357.458(940.964,1934.021) | 230.946(148.899,347.439) | 827.254(532.86,1245.2) | -1.937(-2.064,-1.809) |
| Central Europe | 148.866(95.802,224.018) | 523.376(337.627,785.239) | 43.266(27.601,65.181) | 250.852(160.069,377.32) | -2.556(-2.649,-2.463) |
| Central Latin America | 1258.609(1126.697,1421.392) | 1925.337(1722.544,2176.05) | 271.125(207.736,354.925) | 451.529(347.023,589.815) | -4.468(-4.576,-4.360) |
| Central Sub-Saharan Africa | 2179.159(1593.094,3183.954) | 7523.143(5477.808,11007.192) | 929.607(617.555,1332.207) | 1560.729(1035.433,2240.817) | -5.172(-5.622,-4.720) |
| East Asia | 3545.256(2882.377,4371.964) | 1073.589(872.73,1324.184) | 340.968(241.262,469.972) | 133.678(95.07,183.5) | -9.684(-11.752,-7.567) |
| Eastern Europe | 228.779(150.341,342.796) | 450.622(296.812,673.763) | 86.8(53.285,133.859) | 255.708(158.059,393.18) | -2.214(-2.462,-1.966) |
| Eastern Sub-Saharan Africa | 9984.038(7742.524,13206.937) | 9870.165(7652.631,13054.41) | 4078.887(3122.344,5248.553) | 2254.984(1724.984,2904.184) | -4.711(-5.414,-4.002) |
| High-income Asia Pacific | 26.721(14.951,48.599) | 80.51(45.079,146.587) | 9.021(5.002,16.067) | 42.606(23.64,76.223) | -1.891(-2.111,-1.670) |
| High-income North America | 29.913(17.727,49.438) | 48.519(28.75,80.181) | 34.7(22.251,52.676) | 52.663(33.828,79.887) | 0.759(0.480,1.038) |
| North Africa and Middle East | 2387.238(1814.977,3161.663) | 1657.375(1258.331,2196.991) | 1315.399(942.16,1834.277) | 730.609(524.099,1018.319) | -2.696(-2.813,-2.579) |
| Oceania | 32.533(23.293,46.391) | 1160.702(825.945,1668.202) | 49.952(33.025,78.321) | 933.642(612.123,1479.737) | -0.416(-0.572,-0.261) |
| South Asia | 24452.207(19826.966,30382.117) | 5503.612(4457.223,6847.716) | 8227.655(5822.152,11468.777) | 1693.018(1204.4,2349.151) | -3.667(-3.713,-3.621) |
| Southeast Asia | 3287.511(2585.153,4290.533) | 1958.937(1541.829,2555.04) | 1072.634(768.275,1492.148) | 643.232(462.126,892.356) | -3.604(-3.717,-3.490) |
| Southern Latin America | 109.438(89.445,151.324) | 742.165(607.151,1024.686) | 19.453(10.895,38.645) | 145.998(82.297,286.337) | -5.146(-5.402,-4.889) |
| Southern Sub-Saharan Africa | 779.109(630.448,971.866) | 3681.335(2974.294,4596.809) | 580.836(446.409,743.497) | 2485.625(1914.898,3175.286) | -0.465(-0.814,-0.115) |
| Tropical Latin America | 1198.118(963.791,1494.358) | 2377.003(1931.068,2938.832) | 318.31(195.174,492.605) | 637.312(391.435,984.567) | -4.601(-4.802,-4.401) |
| Western Europe | 107.758(68.661,161.815) | 157.039(100.154,235.331) | 65.333(40.9,100.157) | 99.636(62.389,152.684) | -1.517(-1.670,-1.364) |
| Western Sub-Saharan Africa | 5348.348(4093.347,7347.666) | 5403.377(4122.856,7436.823) | 4817.808(3486.172,6526.995) | 2171.984(1566.052,2952.39) | -2.955(-3.072,-2.838) |

ASDR = age standardized deaths rate; EAPC = estimated annual percentage change; SDI = socio-demographic index; 95% CI = 95% confidence interval.
