# Supplementary material for: Trends and cross-country inequality in the global burden of nutritional deficiencies in children, with projections to 2035: results from the Global Burden of Disease study 2021
Source: Front Nutr. 2025 Jul 29;12:1615593. doi: 10.3389/fnut.2025.1615593 (PMC12340229; doi:10.3389/fnut.2025.1615593)
Supplement: Supplementary file 11 [file Table_3.docx]

**Table S3** Age standardized deaths rate (ASMR) of nutritional deficiencies in 1990 and 2021, and estimated annual percent age change (EAPC) from 1990 to 2021 at the global and regional level.

| Group | **1990** | | **2021** | | **1990-2021** |
| --- | --- | --- | --- | --- | --- |
|  | **Deaths cases,**  **(95%CI)** | **ASMRs**  **per 100000**  **(95%CI)** | **Deaths cases,**  **(95%CI)** | **ASMRs**  **per 100000**  **(95%CI)** | **EAPC, %,**  **(95%CI)** |
| Global | 431263.714(355345.528,544719.92) | 24.385(20.09,30.799) | 83824.622(64402.076,104264.061) | 4.423(3.395,5.505) | -5.522(-6.052,-4.989) |
| SDI |  |  |  |  |  |
| High | 430.475(329.884,599.669) | 0.242(0.185,0.337) | 494.933(407.629,593.01) | 0.05(0.044,0.055) | -5.190(-5.910,-4.464) |
| High-middle | 8747.305(7580.095,10082.331) | 3.287(2.848,3.789) | 79.391(69.846,87.494) | 0.239(0.196,0.286) | -8.684(-8.971,-8.396) |
| Middle | 64863.677(57510.269,74139.934) | 11.314(10.031,12.932) | 7030.774(5869.333,8377.876) | 1.365(1.137,1.629) | -6.365(-6.503,-6.226) |
| Low-middle | 181347.954(148228.935,221895.193) | 36.743(30.018,44.966) | 54358.831(38878.164,70494.678) | 3.954(3.147,4.874) | -7.469(-8.070,-6.864) |
| Low | 175648.188(132889.32,236038.658) | 68.328(51.689,91.792) | 21793.273(17358.031,26854.921) | 11.525(8.246,14.942) | -5.456(-6.052,-4.857) |
| Regions |  |  |  |  |  |
| Andean Latin America | 4427.309(3560.239,5452.55) | 29.379(23.625,36.183) | 450.745(323.096,601.045) | 2.548(1.826,3.398) | -7.931(-8.231,-7.629) |
| Australasia | 1.861(1.638,2.093) | 0.042(0.037,0.047) | 0.644(0.525,0.783) | 0.012(0.01,0.015) | -3.272(-3.565,-2.977) |
| Caribbean | 3213.941(2582.585,3963.281) | 27.294(21.932,33.659) | 908.88(631.815,1299.624) | 8.199(5.698,11.727) | -3.543(-3.909,-3.176) |
| Central Asia | 580.064(513.493,661.244) | 2.147(1.901,2.447) | 79.16(60.571,105.91) | 0.278(0.213,0.372) | -7.293(-7.684,-6.900) |
| Central Europe | 48.039(39.197,59.132) | 0.181(0.147,0.223) | 13.834(11.315,16.613) | 0.084(0.069,0.101) | -3.094(-3.697,-2.487) |
| Central Latin America | 11388.904(10505.316,12463.602) | 17.382(16.034,19.02) | 1450.456(1104.002,1914.205) | 2.471(1.874,3.271) | -5.946(-6.068,-5.825) |
| Central Sub-Saharan Africa | 21128.823(14997.508,31691.342) | 71.833(50.973,107.71) | 5800.681(3472.556,8685.882) | 9.662(5.786,14.466) | -6.322(-6.891,-5.750) |
| East Asia | 25224.256(20595.88,30734.204) | 7.631(6.231,9.298) | 641.658(506.092,796.254) | 0.272(0.214,0.338) | -14.657(-17.461,-11.759) |
| Eastern Europe | 233.683(207.128,261.658) | 0.471(0.417,0.527) | 22.968(20.654,25.213) | 0.075(0.067,0.083) | -7.572(-8.593,-6.539) |
| Eastern Sub-Saharan Africa | 98539.56(74848.204,133107.786) | 96.554(73.337,130.391) | 28097.585(20319.773,36301.356) | 15.46(11.184,19.97) | -5.624(-6.507,-4.734) |
| High-income Asia Pacific | 20.861(17.343,25.011) | 0.065(0.055,0.078) | 4.468(3.947,4.96) | 0.022(0.02,0.025) | -3.378(-3.537,-3.219) |
| High-income North America | 27.218(26.291,28.168) | 0.044(0.042,0.046) | 38.249(34.043,42.315) | 0.063(0.056,0.07) | 0.871(-0.047,1.797) |
| North Africa and Middle East | 11835.647(8918.684,17790.761) | 8.118(6.118,12.196) | 2417.777(1830.564,3135.871) | 1.372(1.039,1.78) | -5.699(-6.068,-5.329) |
| Oceania | 142.707(105.826,188.242) | 5.013(3.714,6.617) | 130.105(88.544,187.587) | 2.38(1.62,3.432) | -2.221(-2.466,-1.976) |
| South Asia | 173629.499(137606.376,213917.977) | 38.782(30.729,47.786) | 14669.383(10917.952,19439.246) | 3.197(2.377,4.241) | -7.397(-7.687,-7.107) |
| Southeast Asia | 20009.851(15344.346,27635.63) | 11.977(9.184,16.542) | 2601.29(2041.919,3213.744) | 1.593(1.249,1.97) | -6.391(-6.510,-6.273) |
| Southern Latin America | 887.531(825.613,954.485) | 6.03(5.61,6.485) | 51.234(42.433,62.322) | 0.401(0.331,0.489) | -8.383(-8.986,-7.776) |
| Southern Sub-Saharan Africa | 6099.787(4937.108,7727.56) | 28.631(23.173,36.268) | 3771.038(2808.418,4836.764) | 16.35(12.174,20.972) | -0.509(-1.115,0.100) |
| Tropical Latin America | 8377.932(7424.85,9358.552) | 17.098(15.153,19.101) | 454.81(356.85,565.741) | 0.92(0.722,1.145) | -9.538(-10.130,-8.942) |
| Western Europe | 36.205(33.906,38.577) | 0.054(0.051,0.058) | 14.107(11.985,16.18) | 0.022(0.019,0.026) | -2.387(-2.746,-2.028) |
| Western Sub-Saharan Africa | 45410.037(33206.807,64094.459) | 44.772(32.739,63.162) | 22205.551(14934.579,29929.395) | 9.764(6.571,13.157) | -4.727(-4.917,-4.536) |

ASMR=age standardized deaths rate;EAPC= estimated annual percentage change;SDI= socio-demographic index; 95%CI=95% confidence interval.
