# Supplementary material for: Trends and cross-country inequality in the global burden of nutritional deficiencies in children, with projections to 2035: results from the Global Burden of Disease study 2021
Source: Front Nutr. 2025 Jul 29;12:1615593. doi: 10.3389/fnut.2025.1615593 (PMC12340229; doi:10.3389/fnut.2025.1615593)
Supplement: Supplementary file 12 [file Table_4.docx]

**Table S4** Age standardized incidence, prevalence, DALYs, and deaths rate of of nutritional deficiencies in 1990 and 2021, and estimated annual percentage change (EAPC) from 1990 to 2021 by 204 countries and territories

| **Locations** | **Age standardized rate in 2021 (per 100 000 population) (95% CI)** | | | | **EAPC in age standardized rate (%) from 1990 to 2021 (95% CI)** | | | |
| --- | --- | --- | --- | --- | --- | --- | --- | --- |
|  | **Incidence** | **Prevalence** | **DALYs** | **Deaths** | **Incidence** | **Prevalence** | **DALYs** | **Deaths** |
| Afghanistan | 31564.133 ( 23286.09 , 40973.937 ) | 40189.402 ( 32388.399 , 48565.722 ) | 909.899 ( 601.896 , 1351.386 ) | 4.198 ( 2.67 , 6.343 ) | -1.943(-2.313,-1.572) | -1.501(-1.767,-1.234) | -3.790(-4.139,-3.440) | -5.099(-5.572,-4.624) |
| Albania | 14895.6 ( 10273.55 , 20561.472 ) | 25304.79 ( 19789.814 , 34335.348 ) | 314.611 ( 186.944 , 495.289 ) | 0.431 ( 0.217 , 0.777 ) | -3.204(-3.441,-2.967) | -2.525(-2.666,-2.384) | -4.275(-4.490,-4.060) | -5.516(-5.995,-5.035) |
| Algeria | 4874.453 ( 3492.474 , 6803.538 ) | 19635.741 ( 15764.977 , 26788.488 ) | 475.978 ( 268.251 , 773.877 ) | 0.264 ( 0.176 , 0.383 ) | -3.444(-3.532,-3.356) | -1.668(-1.688,-1.648) | -3.043(-3.133,-2.952) | -7.776(-8.128,-7.423) |
| American Samoa | 8993.509 ( 5773.776 , 13449.428 ) | 22209.963 ( 17625.936 , 27975.777 ) | 678.57 ( 456.271 , 979.459 ) | 2.468 ( 1.477 , 3.701 ) | -1.607(-1.853,-1.360) | -0.818(-0.899,-0.736) | -1.263(-1.370,-1.156) | -2.539(-2.814,-2.264) |
| Andorra | 1251.618 ( 872.815 , 1747.183 ) | 4872.204 ( 3669.561 , 6649.271 ) | 81.657 ( 36.955 , 152.712 ) | 0 ( 0 , 0 ) | -2.437(-2.528,-2.346) | -1.514(-1.665,-1.362) | -1.027(-1.242,-0.812) | -5.652(-6.097,-5.204) |
| Angola | 21729.122 ( 15107.176 , 29952.532 ) | 33205.247 ( 26650.127 , 41115.925 ) | 1576.249 ( 1041.358 , 2233.139 ) | 12.136 ( 7.496 , 17.134 ) | -3.078(-3.289,-2.867) | -1.951(-2.130,-1.772) | -7.517(-7.791,-7.241) | -8.450(-8.793,-8.106) |
| Antigua and Barbuda | 2900.13 ( 2102.151 , 4017.347 ) | 24907.664 ( 17320.343 , 41211.802 ) | 598.497 ( 401.079 , 982.834 ) | 1.899 ( 1.525 , 2.316 ) | -2.460(-2.592,-2.327) | -1.068(-1.133,-1.004) | -1.589(-1.859,-1.318) | -1.936(-2.691,-1.174) |
| Argentina | 8747.55 ( 5534.581 , 13223.019 ) | 18658.777 ( 11190.861 , 34308.825 ) | 158.846 ( 76.18 , 346.889 ) | 0.403 ( 0.323 , 0.501 ) | -2.158(-2.346,-1.971) | -1.618(-1.691,-1.545) | -5.548(-5.836,-5.260) | -9.157(-9.750,-8.561) |
| Armenia | 1515.073 ( 1110.744 , 1973.783 ) | 18003.05 ( 14444.987 , 22666.566 ) | 586.379 ( 335.656 , 956.889 ) | 0.182 ( 0.142 , 0.232 ) | -0.774(-0.978,-0.569) | -0.657(-0.697,-0.618) | -2.296(-2.381,-2.212) | -8.696(-9.220,-8.170) |
| Australia | 434.92 ( 310.562 , 586.599 ) | 4209.72 ( 2189.176 , 10152.728 ) | 40.129 ( 15.698 , 96.72 ) | 0.014 ( 0.011 , 0.017 ) | -0.775(-0.948,-0.602) | -1.415(-1.569,-1.261) | -1.485(-1.667,-1.302) | -3.333(-3.651,-3.015) |
| Austria | 1505.358 ( 1062.475 , 2096.283 ) | 5233.293 ( 3917.463 , 7038.799 ) | 81.853 ( 36.625 , 165.574 ) | 0.003 ( 0.002 , 0.003 ) | -2.164(-2.418,-1.908) | -1.752(-1.902,-1.601) | -1.843(-2.057,-1.629) | -2.303(-2.865,-1.737) |
| Azerbaijan | 4427.638 ( 3235.771 , 6013.816 ) | 21869.127 ( 17950.655 , 26790.949 ) | 735.987 ( 429.202 , 1148.377 ) | 0.459 ( 0.284 , 0.707 ) | -1.230(-1.762,-0.695) | -1.308(-1.493,-1.122) | -2.680(-2.829,-2.531) | -7.971(-8.306,-7.635) |
| Bahamas | 1718.519 ( 1194.634 , 2491.738 ) | 20837.35 ( 15303.27 , 33007.414 ) | 543.886 ( 332.154 , 881.318 ) | 0.97 ( 0.705 , 1.374 ) | -2.112(-2.241,-1.984) | -0.685(-0.806,-0.565) | -1.643(-1.935,-1.350) | -4.400(-5.149,-3.644) |
| Bahrain | 2943.891 ( 2212.477 , 3954.432 ) | 13638.821 ( 10645.423 , 17998.249 ) | 286.353 ( 153.372 , 506.606 ) | 0.235 ( 0.176 , 0.31 ) | -2.339(-2.496,-2.182) | -1.640(-1.668,-1.612) | -2.904(-3.042,-2.766) | -4.671(-5.090,-4.251) |
| Bangladesh | 6492.493 ( 4936.522 , 8524.805 ) | 24861.352 ( 20392.035 , 33610.826 ) | 869.715 ( 582.142 , 1264.55 ) | 2.696 ( 1.599 , 3.967 ) | -4.411(-4.656,-4.164) | -2.249(-2.278,-2.220) | -8.037(-8.251,-7.823) | -11.280(-11.611,-10.948) |
| Barbados | 2841.419 ( 2038.639 , 3944.618 ) | 20275.928 ( 15208.817 , 32546.37 ) | 468.114 ( 275.684 , 767.894 ) | 0.505 ( 0.34 , 0.728 ) | -1.494(-1.642,-1.346) | -0.741(-0.795,-0.688) | -1.775(-1.960,-1.589) | -5.784(-6.193,-5.373) |
| Belarus | 1043.288 ( 745.237 , 1410.268 ) | 9999.422 ( 7718.575 , 13831.029 ) | 221.278 ( 128.175 , 375.732 ) | 0.192 ( 0.139 , 0.259 ) | -2.825(-2.896,-2.754) | -1.600(-1.736,-1.462) | -2.405(-2.603,-2.208) | -5.634(-6.828,-4.426) |
| Belgium | 1193.176 ( 843.595 , 1648.635 ) | 5271.945 ( 3969.732 , 7375.651 ) | 91.964 ( 44.636 , 175.761 ) | 0.025 ( 0.02 , 0.03 ) | -2.708(-2.910,-2.506) | -1.711(-1.801,-1.620) | -1.658(-1.814,-1.503) | -2.420(-2.862,-1.976) |
| Belize | 5511.305 ( 3568.789 , 8201.564 ) | 26507.979 ( 20202.288 , 39827.64 ) | 891.184 ( 603.954 , 1315.317 ) | 3.3 ( 2.551 , 4.231 ) | -2.972(-3.054,-2.890) | -0.882(-0.940,-0.824) | -3.226(-3.646,-2.804) | -5.755(-6.533,-4.971) |
| Benin | 33893.941 ( 24837.575 , 43645.247 ) | 48177.604 ( 38490.647 , 58040.938 ) | 1451.067 ( 846.912 , 2269.046 ) | 6.478 ( 3.598 , 10.172 ) | -2.062(-2.155,-1.969) | -1.225(-1.269,-1.181) | -4.632(-4.800,-4.463) | -6.812(-7.097,-6.526) |
| Bermuda | 1625.336 ( 1183.343 , 2227.881 ) | 11830.107 ( 9038.625 , 17364.902 ) | 215.233 ( 120.19 , 351.775 ) | 0.133 ( 0.099 , 0.17 ) | -2.536(-2.579,-2.493) | -1.674(-1.806,-1.542) | -2.194(-2.438,-1.949) | -5.387(-6.094,-4.674) |
| Bhutan | 6574.515 ( 4644.251 , 9302.111 ) | 52113.229 ( 40794.525 , 65253.789 ) | 2020.794 ( 991.016 , 3304.227 ) | 0.88 ( 0.36 , 1.592 ) | -5.348(-5.490,-5.206) | -0.487(-0.554,-0.420) | -1.695(-1.828,-1.561) | -8.966(-9.287,-8.645) |
| Bolivia (Plurinational State of) | 6546.466 ( 4223.069 , 9890.445 ) | 29890.038 ( 23437.176 , 41674.91 ) | 1323.479 ( 911.142 , 1930.227 ) | 5.552 ( 3.662 , 8.244 ) | -2.662(-2.886,-2.438) | -0.921(-0.946,-0.896) | -5.217(-5.391,-5.043) | -7.540(-7.633,-7.446) |
| Bosnia and Herzegovina | 11439.879 ( 7544.276 , 16861.264 ) | 22282.391 ( 17437.832 , 29447.228 ) | 287.784 ( 160.143 , 490.401 ) | 0.029 ( 0.019 , 0.041 ) | -3.459(-3.714,-3.203) | -2.287(-2.474,-2.100) | -2.541(-2.797,-2.284) | 2.603(1.993,3.217) |
| Botswana | 12806.717 ( 8646.264 , 18163.252 ) | 35126.489 ( 26341.648 , 47547.456 ) | 1859.76 ( 1224.066 , 2929.698 ) | 12.907 ( 7.882 , 19.547 ) | -3.181(-3.257,-3.105) | -1.274(-1.342,-1.206) | -0.398(-0.675,-0.119) | 0.026(-0.513,0.567) |
| Brazil | 10773.67 ( 6945.449 , 15743.706 ) | 24838.642 ( 19900.221 , 31615.478 ) | 640.723 ( 388.895 , 1000.493 ) | 0.868 ( 0.687 , 1.079 ) | -3.213(-3.329,-3.096) | -1.825(-1.871,-1.780) | -4.660(-4.865,-4.454) | -9.774(-10.387,-9.157) |
| Brunei Darussalam | 2495.092 ( 1826.47 , 3362.714 ) | 7770.247 ( 5855.069 , 11912.808 ) | 75.681 ( 37.133 , 143.937 ) | 0.028 ( 0.018 , 0.041 ) | -2.514(-2.640,-2.387) | -1.538(-1.702,-1.373) | -1.439(-1.708,-1.170) | -2.020(-2.203,-1.836) |
| Bulgaria | 10852.888 ( 7731.805 , 15016.471 ) | 22859.191 ( 18250.397 , 29939.34 ) | 323.524 ( 174.507 , 544.521 ) | 0.078 ( 0.061 , 0.097 ) | -1.434(-1.637,-1.231) | -1.008(-1.137,-0.878) | -1.466(-1.621,-1.311) | 0.456(-0.113,1.029) |
| Burkina Faso | 32208.609 ( 22893.533 , 42671.437 ) | 55925.33 ( 47310.719 , 64640.571 ) | 3546.146 ( 2369.703 , 5193.804 ) | 20.106 ( 12.378 , 30.647 ) | -3.119(-3.259,-2.979) | -1.301(-1.344,-1.258) | -2.598(-2.913,-2.282) | -4.000(-4.405,-3.592) |
| Burundi | 18847.102 ( 13105.522 , 25914.024 ) | 38816.714 ( 29604.405 , 48746.657 ) | 2174.433 ( 1179.72 , 3633.283 ) | 14.805 ( 7.039 , 25.821 ) | -3.246(-3.552,-2.940) | -1.244(-1.342,-1.145) | -5.849(-6.076,-5.621) | -6.929(-7.276,-6.580) |
| Cabo Verde | 6355.566 ( 4013.995 , 9819.049 ) | 31423.207 ( 23579.361 , 45490.801 ) | 857.038 ( 509.409 , 1465.822 ) | 1.486 ( 0.985 , 2.254 ) | -5.662(-5.838,-5.485) | -1.899(-2.028,-1.770) | -5.151(-5.612,-4.688) | -10.024(-10.546,-9.498) |
| Cambodia | 12966.371 ( 8830.813 , 18630.999 ) | 36412.054 ( 28658.912 , 48183.395 ) | 987.691 ( 596.717 , 1596.554 ) | 2.405 ( 1.552 , 3.711 ) | -4.380(-4.439,-4.322) | -1.766(-1.818,-1.714) | -6.478(-6.817,-6.139) | -10.712(-11.225,-10.195) |
| Cameroon | 21760.919 ( 14827.562 , 30748.617 ) | 38691.191 ( 30839.884 , 48202.185 ) | 1100.774 ( 653.952 , 1753.945 ) | 3.914 ( 2.294 , 5.988 ) | -3.691(-3.937,-3.446) | -2.071(-2.252,-1.890) | -2.681(-2.888,-2.472) | -4.852(-5.202,-4.501) |
| Canada | 1564.235 ( 1064.437 , 2251.065 ) | 2894.626 ( 2237.477 , 3714.133 ) | 42.708 ( 18.235 , 72.048 ) | 0.026 ( 0.021 , 0.031 ) | -3.121(-3.330,-2.910) | -2.317(-2.532,-2.101) | 1.385(1.044,1.727) | 0.689(-0.126,1.511) |
| Central African Republic | 41504.864 ( 31730.578 , 52168.712 ) | 55765.096 ( 45774.895 , 66380.115 ) | 3852.049 ( 2505.075 , 5733.47 ) | 31.044 ( 19.325 , 46.055 ) | -0.967(-1.021,-0.912) | -0.567(-0.616,-0.518) | -0.935(-1.291,-0.579) | -1.071(-1.464,-0.676) |
| Chad | 49323.225 ( 38702.292 , 60548.479 ) | 63629.598 ( 54385.479 , 72797.147 ) | 2810.408 ( 1854.164 , 4021.383 ) | 14.6 ( 9.513 , 21.588 ) | -1.769(-1.848,-1.690) | -1.006(-1.040,-0.972) | -3.610(-3.739,-3.480) | -5.248(-5.422,-5.073) |
| Chile | 4595.407 ( 2876.049 , 7133.601 ) | 7993.996 ( 5960.164 , 10725.205 ) | 112.558 ( 75.538 , 172.021 ) | 0.392 ( 0.326 , 0.468 ) | -3.237(-3.458,-3.016) | -2.693(-2.805,-2.581) | -3.942(-4.230,-3.654) | -5.779(-6.467,-5.085) |
| China | 3783.742 ( 2677.964 , 5319.467 ) | 7432.958 ( 6348.418 , 8866.157 ) | 129.928 ( 92.728 , 178.154 ) | 0.268 ( 0.209 , 0.335 ) | -4.894(-5.021,-4.768) | -4.109(-4.187,-4.031) | -7.445(-7.827,-7.062) | -11.757(-12.328,-11.182) |
| Colombia | 2971.691 ( 1934.878 , 4326.4 ) | 10590.628 ( 8591.576 , 13038.419 ) | 498.285 ( 349.543 , 709.566 ) | 3.39 ( 2.296 , 4.827 ) | -3.724(-3.801,-3.647) | -2.449(-2.509,-2.389) | -2.410(-2.759,-2.060) | -2.656(-3.175,-2.134) |
| Comoros | 15754.461 ( 10883.931 , 22076.661 ) | 38055.966 ( 27576.797 , 53836.072 ) | 1734.68 ( 1117.263 , 2614.331 ) | 9.825 ( 6.118 , 15.003 ) | -3.551(-3.646,-3.456) | -1.467(-1.555,-1.378) | -4.484(-4.640,-4.328) | -6.062(-6.244,-5.879) |
| Congo | 41643.214 ( 31414.384 , 52902.181 ) | 55213.529 ( 45789.628 , 65406.982 ) | 1267.937 ( 794.508 , 1943.933 ) | 4.682 ( 3.005 , 6.769 ) | -0.964(-1.205,-0.721) | -0.519(-0.703,-0.334) | -2.744(-3.178,-2.308) | -4.831(-5.461,-4.197) |
| Cook Islands | 5847.412 ( 3817.382 , 8830.112 ) | 18168.615 ( 13843.504 , 24808.491 ) | 277.42 ( 156.056 , 448.052 ) | 0.053 ( 0.032 , 0.077 ) | -3.848(-3.984,-3.711) | -2.053(-2.088,-2.018) | -1.939(-2.044,-1.834) | -7.839(-8.812,-6.855) |
| Costa Rica | 3564.49 ( 2539.08 , 5041.792 ) | 11578.706 ( 9186.86 , 14743.682 ) | 215.413 ( 119.831 , 352.621 ) | 0.099 ( 0.077 , 0.126 ) | -2.299(-2.535,-2.061) | -1.320(-1.403,-1.237) | -0.994(-1.155,-0.834) | -7.004(-7.401,-6.605) |
| Croatia | 19452.022 ( 13455.471 , 27030.117 ) | 42168.188 ( 33131.087 , 52856.717 ) | 1404.269 ( 818.232 , 2200.567 ) | 3.971 ( 2.43 , 5.896 ) | -2.721(-2.914,-2.527) | -1.937(-2.041,-1.833) | -2.087(-2.167,-2.006) | -5.881(-6.235,-5.525) |
| Cuba | 7347.829 ( 4845.401 , 10764.066 ) | 15354.54 ( 12075.821 , 19617.08 ) | 198.335 ( 111.344 , 330.649 ) | 0.02 ( 0.015 , 0.025 ) | -2.458(-2.561,-2.355) | -0.768(-0.830,-0.706) | -0.894(-1.021,-0.766) | 0.622(-0.109,1.360) |
| Cyprus | 2688.711 ( 1751.665 , 4030.338 ) | 17021.112 ( 12824.467 , 25766.344 ) | 360.497 ( 202.534 , 596.792 ) | 0.205 ( 0.159 , 0.259 ) | -4.002(-4.414,-3.587) | -2.769(-3.001,-2.537) | -2.712(-2.951,-2.473) | -3.565(-4.688,-2.429) |
| Czechia | 1487.888 ( 1081.767 , 2027.857 ) | 5132.278 ( 3832.625 , 7079.812 ) | 76.665 ( 35.196 , 145.79 ) | 0.004 ( 0.003 , 0.006 ) | -2.606(-2.802,-2.410) | -1.794(-1.930,-1.658) | -1.971(-2.144,-1.798) | -5.926(-6.229,-5.621) |
| Côte d'Ivoire | 5352.281 ( 3560.256 , 7890.311 ) | 14335.531 ( 11178.842 , 18841.812 ) | 203.753 ( 116.63 , 335.601 ) | 0.14 ( 0.108 , 0.177 ) | -3.004(-3.099,-2.909) | -1.097(-1.173,-1.020) | -2.712(-2.950,-2.474) | -0.160(-0.787,0.470) |
| Democratic People's Republic of Korea | 10811.235 ( 7044.142 , 15748.515 ) | 20606.413 ( 16261.574 , 25789.777 ) | 334.629 ( 203.349 , 546.119 ) | 0.561 ( 0.351 , 0.839 ) | -2.674(-2.981,-2.366) | -2.148(-2.317,-1.978) | -12.428(-18.249,-6.192) | -16.583(-23.523,-9.013) |
| Democratic Republic of the Congo | 33069.771 ( 24347.147 , 43732.574 ) | 46734.222 ( 38048.863 , 56412.348 ) | 1440.337 ( 839.306 , 2320.296 ) | 7.74 ( 3.743 , 13.632 ) | -0.984(-1.490,-0.474) | -0.716(-1.029,-0.401) | -4.654(-5.247,-4.057) | -5.914(-6.696,-5.126) |
| Denmark | 926.852 ( 688.286 , 1225.042 ) | 5048.334 ( 3649.588 , 7220.018 ) | 81.946 ( 38.31 , 152.232 ) | 0.023 ( 0.018 , 0.028 ) | -2.402(-2.658,-2.146) | -1.832(-1.936,-1.728) | -2.187(-2.356,-2.018) | -3.095(-3.723,-2.462) |
| Djibouti | 16808.279 ( 12029.196 , 23201.526 ) | 39467.38 ( 30851.615 , 53164.497 ) | 1963.924 ( 1295.883 , 2953.924 ) | 10.343 ( 5.86 , 16.258 ) | -2.714(-2.908,-2.521) | -1.107(-1.198,-1.016) | -3.825(-4.223,-3.426) | -5.239(-5.779,-4.695) |
| Dominica | 2404.973 ( 1621.588 , 3527.393 ) | 19953.672 ( 16169.972 , 24935.905 ) | 820.754 ( 533.285 , 1229.189 ) | 2.737 ( 1.7 , 4.244 ) | -3.374(-3.552,-3.195) | -0.901(-1.004,-0.798) | -0.528(-0.799,-0.256) | -0.498(-1.304,0.316) |
| Dominican Republic | 5206.757 ( 3287.908 , 8053.676 ) | 20976.208 ( 17158.532 , 25771.895 ) | 903.24 ( 583.524 , 1340.932 ) | 4.06 ( 2.276 , 6.257 ) | -3.877(-3.969,-3.784) | -1.521(-1.648,-1.393) | -4.075(-4.610,-3.537) | -6.275(-6.880,-5.666) |
| Ecuador | 5033.088 ( 3307.375 , 7447.312 ) | 10592.074 ( 8430.623 , 13364.815 ) | 261.291 ( 195.936 , 349.453 ) | 1.744 ( 1.214 , 2.399 ) | -2.970(-3.218,-2.722) | -2.443(-2.483,-2.402) | -6.215(-6.873,-5.552) | -7.388(-8.255,-6.513) |
| Egypt | 3714.473 ( 2685.654 , 5136.745 ) | 17073.576 ( 14346.093 , 20208.582 ) | 420.149 ( 263.567 , 640.892 ) | 0.538 ( 0.363 , 0.772 ) | -2.581(-2.926,-2.234) | -1.409(-1.525,-1.292) | -2.437(-2.621,-2.254) | -4.997(-5.420,-4.572) |
| El Salvador | 6252.863 ( 3929.236 , 9559.683 ) | 16632.79 ( 13099.651 , 21241.214 ) | 324.993 ( 216.364 , 474.916 ) | 1.229 ( 0.794 , 1.736 ) | -4.272(-4.595,-3.948) | -2.162(-2.446,-1.878) | -4.706(-4.999,-4.413) | -7.120(-7.276,-6.964) |
| Equatorial Guinea | 8022.346 ( 5156.153 , 11859.133 ) | 28973.1 ( 20705.573 , 41428.442 ) | 958.034 ( 537.437 , 1603.563 ) | 3.899 ( 1.911 , 6.876 ) | -7.018(-7.262,-6.774) | -3.379(-3.653,-3.104) | -6.080(-6.563,-5.596) | -8.061(-8.544,-7.574) |
| Eritrea | 23550.076 ( 16739.157 , 32301.485 ) | 45710.195 ( 36381.965 , 59522.379 ) | 2804.131 ( 1908.998 , 4172.406 ) | 19.549 ( 12.31 , 28.93 ) | -2.957(-3.118,-2.796) | -1.300(-1.372,-1.229) | -5.499(-5.814,-5.184) | -6.676(-6.976,-6.375) |
| Estonia | 981.077 ( 712.755 , 1296.003 ) | 9177.836 ( 7152.757 , 12135.396 ) | 188.587 ( 108.605 , 318.214 ) | 0.098 ( 0.081 , 0.122 ) | -2.939(-3.064,-2.813) | -1.917(-2.024,-1.810) | -2.953(-3.165,-2.740) | -7.465(-8.612,-6.304) |
| Eswatini | 12264.265 ( 8065.499 , 17876.731 ) | 32545.856 ( 25000.548 , 44830.528 ) | 1613.838 ( 1110.933 , 2487.009 ) | 11.107 ( 7.182 , 16.582 ) | -3.333(-3.396,-3.271) | -1.221(-1.251,-1.190) | -1.274(-1.501,-1.047) | -1.757(-2.065,-1.447) |
| Ethiopia | 19738.831 ( 13675.61 , 27361.789 ) | 40555.047 ( 34863.459 , 47614.277 ) | 2189.335 ( 1605.554 , 2929.773 ) | 13.58 ( 9.459 , 18.195 ) | -3.994(-4.352,-3.635) | -2.012(-2.152,-1.872) | -5.905(-6.242,-5.567) | -7.305(-7.719,-6.889) |
| Fiji | 10125.631 ( 6603.223 , 15045.097 ) | 29348.118 ( 22480.769 , 42308.518 ) | 748.404 ( 476.184 , 1143.518 ) | 1.949 ( 1.152 , 3.129 ) | -2.433(-2.546,-2.320) | -0.682(-0.756,-0.607) | -0.766(-0.869,-0.664) | -0.942(-1.387,-0.494) |
| Finland | 1315.188 ( 941.568 , 1755.318 ) | 5117.761 ( 3859.122 , 7153.727 ) | 79.303 ( 36.66 , 147.339 ) | 0.005 ( 0.004 , 0.006 ) | -2.382(-2.526,-2.238) | -1.842(-1.963,-1.721) | -2.192(-2.408,-1.974) | -2.195(-2.499,-1.891) |
| France | 783.552 ( 582.574 , 1078.303 ) | 4758.335 ( 3504.333 , 6736.757 ) | 96.414 ( 48.427 , 173.454 ) | 0.082 ( 0.066 , 0.098 ) | -0.662(-0.884,-0.439) | -1.482(-1.544,-1.420) | -2.070(-2.267,-1.872) | -2.462(-2.937,-1.984) |
| Gabon | 8567.839 ( 5532.545 , 12927.587 ) | 34793.152 ( 25349.622 , 48011.388 ) | 1084.018 ( 558.617 , 1908.496 ) | 2.914 ( 1.586 , 4.857 ) | -2.858(-3.077,-2.638) | -1.131(-1.357,-0.904) | -2.356(-2.725,-1.986) | -3.387(-3.906,-2.864) |
| Gambia | 26894.651 ( 18707.835 , 36974.368 ) | 54753.103 ( 45422.564 , 64490.781 ) | 2184.759 ( 1364.812 , 3260.094 ) | 6.875 ( 4.547 , 9.968 ) | -2.534(-2.583,-2.484) | -1.073(-1.120,-1.027) | -3.643(-3.873,-3.411) | -6.330(-6.654,-6.006) |
| Georgia | 2264.668 ( 1518.75 , 3410.218 ) | 19499.114 ( 14972.696 , 27175.639 ) | 555.119 ( 306.669 , 939.504 ) | 0.033 ( 0.025 , 0.043 ) | -1.181(-1.748,-0.611) | -0.626(-0.674,-0.579) | -0.911(-0.979,-0.843) | -8.879(-9.635,-8.117) |
| Germany | 778.895 ( 517.389 , 1054.628 ) | 4685.269 ( 3412.833 , 6866.652 ) | 86.668 ( 40.904 , 152.847 ) | 0.007 ( 0.006 , 0.009 ) | -1.420(-1.611,-1.229) | -1.504(-1.673,-1.335) | -1.429(-1.566,-1.292) | -1.784(-2.274,-1.291) |
| Ghana | 25027.431 ( 17235.161 , 34752.142 ) | 47662.761 ( 37479.382 , 58843.816 ) | 1669.331 ( 987.433 , 2624.458 ) | 6.838 ( 3.931 , 10.558 ) | -2.790(-2.935,-2.645) | -1.189(-1.265,-1.114) | -3.688(-3.854,-3.522) | -5.727(-5.986,-5.467) |
| Greece | 2069.524 ( 1460.052 , 2949.774 ) | 6413.673 ( 4726.43 , 8900.369 ) | 99.214 ( 44.588 , 199.199 ) | 0.002 ( 0.001 , 0.002 ) | -2.159(-2.543,-1.772) | -1.272(-1.501,-1.042) | -1.137(-1.336,-0.938) | -0.430(-0.995,0.137) |
| Greenland | 1282.027 ( 864.119 , 1859.705 ) | 4239.48 ( 3122.2 , 5988.403 ) | 47.129 ( 22.321 , 85.56 ) | 0.023 ( 0.016 , 0.03 ) | -2.384(-2.707,-2.061) | -2.053(-2.144,-1.963) | -2.090(-2.264,-1.917) | -3.435(-4.090,-2.776) |
| Grenada | 4026.257 ( 2662.381 , 6049.33 ) | 23673.601 ( 18134.161 , 34824.847 ) | 728.072 ( 470.042 , 1116.374 ) | 2.107 ( 1.604 , 2.736 ) | -4.100(-4.361,-3.838) | -1.113(-1.229,-0.996) | -1.518(-1.849,-1.187) | -2.944(-4.059,-1.817) |
| Guam | 4592.978 ( 2983.527 , 6886.739 ) | 18513.356 ( 14090.191 , 25516.703 ) | 390.991 ( 249.511 , 586.526 ) | 1.027 ( 0.711 , 1.458 ) | -2.619(-2.776,-2.462) | -1.065(-1.213,-0.918) | -0.931(-1.139,-0.722) | -1.518(-1.837,-1.198) |
| Guatemala | 4984.556 ( 3246.607 , 7496.372 ) | 20190.483 ( 16077.064 , 28672.349 ) | 955.545 ( 723.758 , 1303.052 ) | 6.35 ( 4.864 , 8.355 ) | -4.156(-4.320,-3.991) | -1.422(-1.571,-1.274) | -4.280(-4.581,-3.979) | -5.354(-5.729,-4.978) |
| Guinea | 30124.573 ( 21651.904 , 40015.523 ) | 49401.43 ( 40207.407 , 59561.038 ) | 2681.467 ( 1676.866 , 4006.032 ) | 16.885 ( 10.029 , 25.515 ) | -2.588(-2.665,-2.511) | -1.315(-1.355,-1.274) | -3.536(-3.793,-3.278) | -4.713(-5.069,-4.357) |
| Guinea-Bissau | 30582.455 ( 21759.217 , 40765.31 ) | 53477.548 ( 44536.275 , 63145.636 ) | 1669.912 ( 984.89 , 2548.746 ) | 3.72 ( 2.025 , 6.153 ) | -2.349(-2.412,-2.286) | -1.009(-1.039,-0.979) | -4.734(-4.918,-4.550) | -8.815(-9.121,-8.508) |
| Guyana | 5188.858 ( 3529.186 , 7422.035 ) | 25964.068 ( 20005.079 , 38674.831 ) | 1033.096 ( 740.028 , 1492.372 ) | 5.408 ( 3.912 , 7.194 ) | -3.523(-3.612,-3.433) | -1.190(-1.270,-1.110) | -3.105(-3.482,-2.727) | -4.298(-4.832,-3.760) |
| Haiti | 16091.201 ( 11061.759 , 23048.912 ) | 49917.273 ( 40255.541 , 61596.401 ) | 2806.029 ( 1824.561 , 4271.848 ) | 16.211 ( 10.764 , 24.132 ) | -2.699(-2.775,-2.623) | -0.355(-0.411,-0.300) | -2.798(-3.029,-2.567) | -4.149(-4.410,-3.887) |
| Honduras | 5129.732 ( 3316.264 , 7748.698 ) | 19529.315 ( 15434.913 , 26122.316 ) | 431.868 ( 271.157 , 679.231 ) | 0.779 ( 0.395 , 1.32 ) | -3.259(-3.337,-3.181) | -1.154(-1.218,-1.089) | -3.450(-3.665,-3.234) | -7.630(-7.775,-7.485) |
| Hungary | 7028.198 ( 4580.544 , 10545.072 ) | 16737.676 ( 12930.237 , 22021.475 ) | 230.047 ( 128.235 , 394.654 ) | 0.079 ( 0.059 , 0.1 ) | -2.630(-2.751,-2.508) | -1.788(-1.865,-1.711) | -2.110(-2.264,-1.956) | 0.478(-0.117,1.076) |
| Iceland | 1097.708 ( 789.786 , 1495.021 ) | 4475.357 ( 3354.43 , 6196.664 ) | 70.594 ( 33.44 , 134.47 ) | 0.009 ( 0.007 , 0.011 ) | -2.545(-2.703,-2.386) | -1.700(-1.812,-1.589) | -1.777(-1.956,-1.597) | -1.911(-2.427,-1.392) |
| India | 15187.043 ( 11008.469 , 21057.574 ) | 46227.255 ( 42584.875 , 50477.447 ) | 1701.921 ( 1207.183 , 2391.877 ) | 2.98 ( 2.128 , 4.236 ) | -3.281(-3.643,-2.918) | -0.959(-1.026,-0.891) | -3.362(-3.475,-3.250) | -7.037(-7.474,-6.599) |
| Indonesia | 11043.947 ( 7687.006 , 15522.115 ) | 21858.158 ( 18250.058 , 26400.173 ) | 655.927 ( 464.606 , 903.827 ) | 2.035 ( 1.45 , 2.664 ) | -4.082(-4.172,-3.991) | -2.893(-2.979,-2.806) | -3.829(-3.936,-3.722) | -5.853(-5.965,-5.741) |
| Iran (Islamic Republic of) | 3186.711 ( 2302.604 , 4443.301 ) | 15098.358 ( 11865.083 , 19795.294 ) | 378.896 ( 217.128 , 637.73 ) | 0.118 ( 0.086 , 0.146 ) | -3.882(-4.206,-3.556) | -1.926(-2.038,-1.814) | -3.061(-3.180,-2.942) | -8.739(-9.591,-7.879) |
| Iraq | 5284.112 ( 3775.572 , 7478.18 ) | 19544.216 ( 15628.475 , 25150.785 ) | 504.404 ( 300.335 , 800.502 ) | 0.519 ( 0.354 , 0.744 ) | -3.875(-4.325,-3.423) | -1.862(-2.031,-1.692) | -3.132(-3.314,-2.949) | -6.443(-6.775,-6.110) |
| Ireland | 1126.394 ( 769.296 , 1593.713 ) | 4387.83 ( 3367.723 , 6225.925 ) | 77.095 ( 36.229 , 143.504 ) | 0.005 ( 0.004 , 0.007 ) | -3.143(-3.391,-2.893) | -2.399(-2.513,-2.285) | -2.429(-2.636,-2.221) | -3.261(-3.654,-2.867) |
| Israel | 6510.791 ( 4312.798 , 9638.791 ) | 10842.655 ( 8051.183 , 14603.261 ) | 107.468 ( 51.752 , 199.643 ) | 0.009 ( 0.007 , 0.011 ) | -2.974(-3.212,-2.736) | -2.378(-2.553,-2.203) | -1.939(-2.087,-1.791) | -3.327(-3.698,-2.954) |
| Italy | 2053.202 ( 1440.791 , 2815.17 ) | 5704.553 ( 4253.798 , 8690.175 ) | 80.858 ( 37.168 , 151.669 ) | 0.015 ( 0.012 , 0.017 ) | -2.268(-2.544,-1.991) | -1.695(-1.843,-1.545) | -1.307(-1.428,-1.186) | 0.635(0.176,1.096) |
| Jamaica | 3377.388 ( 2250.974 , 5077.436 ) | 25192.249 ( 18792.527 , 40570.309 ) | 560.567 ( 333.397 , 918.098 ) | 0.896 ( 0.643 , 1.214 ) | -2.883(-2.990,-2.775) | -0.615(-0.736,-0.494) | -2.815(-3.463,-2.163) | -7.062(-8.234,-5.875) |
| Japan | 1773.119 ( 1278.566 , 2331.088 ) | 5206.463 ( 3707.099 , 9010.425 ) | 45.594 ( 22.957 , 87.464 ) | 0.025 ( 0.022 , 0.028 ) | -1.539(-1.717,-1.360) | -1.156(-1.220,-1.092) | -1.112(-1.201,-1.023) | -1.755(-2.094,-1.415) |
| Jordan | 6024.281 ( 3942.587 , 8981.167 ) | 19614.701 ( 16080.522 , 24675.552 ) | 431.36 ( 244.721 , 714.444 ) | 0.157 ( 0.115 , 0.215 ) | -3.138(-3.635,-2.639) | -1.839(-2.003,-1.674) | -2.262(-2.381,-2.143) | -4.897(-5.100,-4.692) |
| Kazakhstan | 10273.192 ( 6585.395 , 15548.312 ) | 31448.585 ( 23313.147 , 45521.641 ) | 590.739 ( 313.795 , 1231.126 ) | 0.212 ( 0.163 , 0.281 ) | -2.563(-2.750,-2.375) | -1.433(-1.547,-1.318) | -3.030(-3.299,-2.761) | -6.380(-6.872,-5.885) |
| Kenya | 29924.336 ( 20961.638 , 40605.384 ) | 38593.696 ( 30705.578 , 48207.539 ) | 1493.059 ( 1186.453 , 1909.423 ) | 11.841 ( 8.924 , 15.589 ) | -2.295(-2.555,-2.033) | -1.717(-1.904,-1.529) | -3.110(-3.293,-2.926) | -3.461(-3.718,-3.203) |
| Kiribati | 33080.418 ( 23569.899 , 43765.233 ) | 46875.076 ( 38308.985 , 56637.178 ) | 2354.011 ( 1590.96 , 3272.177 ) | 17.096 ( 10.804 , 24.808 ) | -1.323(-1.377,-1.269) | -0.753(-0.785,-0.722) | -3.068(-3.123,-3.012) | -3.835(-3.945,-3.725) |
| Kuwait | 1423.137 ( 1047.876 , 1879.123 ) | 12322.671 ( 9522.441 , 17231.977 ) | 280.105 ( 153.885 , 474.238 ) | 0.04 ( 0.032 , 0.05 ) | -3.553(-3.714,-3.391) | -1.384(-1.475,-1.293) | -1.526(-1.711,-1.340) | -3.539(-5.420,-1.621) |
| Kyrgyzstan | 5256.232 ( 3530.692 , 7663.492 ) | 25733.307 ( 20024.618 , 36326.395 ) | 647.5 ( 361.887 , 1118.781 ) | 0.069 ( 0.057 , 0.084 ) | -0.881(-1.155,-0.606) | -0.692(-0.794,-0.591) | -1.701(-1.838,-1.563) | -9.008(-9.725,-8.286) |
| Lao People's Democratic Republic | 16896.754 ( 11502.17 , 23996.539 ) | 31761.842 ( 25657.684 , 41850.691 ) | 852.369 ( 558.43 , 1280.222 ) | 3.6 ( 2.207 , 5.555 ) | -4.082(-4.270,-3.895) | -2.454(-2.555,-2.354) | -6.039(-6.203,-5.874) | -8.310(-8.528,-8.091) |
| Latvia | 1102.326 ( 805.718 , 1437.14 ) | 10588.358 ( 8162.644 , 14081.349 ) | 223.178 ( 127.131 , 367.169 ) | 0.136 ( 0.112 , 0.166 ) | -2.535(-2.700,-2.370) | -1.295(-1.380,-1.210) | -2.329(-2.444,-2.214) | -6.807(-7.842,-5.759) |
| Lebanon | 3290.586 ( 2500.608 , 4394.94 ) | 16255.816 ( 13022.991 , 22043.378 ) | 369.822 ( 202.473 , 604.513 ) | 0.086 ( 0.054 , 0.128 ) | -2.961(-3.269,-2.652) | -1.706(-1.753,-1.658) | -2.282(-2.375,-2.188) | -5.461(-5.782,-5.139) |
| Lesotho | 20738.849 ( 14221.216 , 29586.534 ) | 38560.653 ( 31360.363 , 49476.542 ) | 2195.123 ( 1486.214 , 3235.679 ) | 16.398 ( 9.783 , 25.097 ) | -2.847(-2.892,-2.801) | -1.317(-1.351,-1.283) | -0.558(-0.827,-0.289) | -0.654(-1.053,-0.253) |
| Liberia | 19228.01 ( 12763.762 , 27167.389 ) | 40459.631 ( 31524.688 , 50343.48 ) | 1828.309 ( 1074.348 , 2822.991 ) | 9.169 ( 5.285 , 14.386 ) | -3.122(-3.282,-2.961) | -1.491(-1.609,-1.373) | -6.470(-6.969,-5.969) | -8.508(-9.186,-7.824) |
| Libya | 4086.397 ( 3017.327 , 5578.55 ) | 18100.293 ( 14456.653 , 24087.211 ) | 477.601 ( 275.525 , 774.846 ) | 0.44 ( 0.277 , 0.669 ) | -2.038(-2.281,-1.795) | -0.978(-1.127,-0.828) | -0.910(-1.137,-0.682) | -1.028(-1.690,-0.362) |
| Lithuania | 952.237 ( 689.783 , 1263.555 ) | 10518.248 ( 8013.601 , 14559.313 ) | 218.63 ( 123.949 , 363.273 ) | 0.109 ( 0.09 , 0.131 ) | -3.256(-3.352,-3.159) | -1.180(-1.214,-1.145) | -1.975(-2.043,-1.907) | -6.119(-7.176,-5.049) |
| Luxembourg | 1023.631 ( 748.645 , 1389.353 ) | 4532.782 ( 3376.992 , 6280.393 ) | 68.084 ( 32.963 , 131.17 ) | 0.018 ( 0.014 , 0.022 ) | -2.460(-2.643,-2.276) | -2.021(-2.168,-1.875) | -2.522(-2.767,-2.276) | -3.520(-4.341,-2.692) |
| Madagascar | 23168.619 ( 16566.608 , 30905.696 ) | 39207.636 ( 31728.977 , 49961.079 ) | 3089.184 ( 2084.333 , 4463.24 ) | 26.309 ( 16.803 , 38.023 ) | -1.988(-2.222,-1.754) | -1.127(-1.214,-1.040) | -4.638(-4.839,-4.437) | -5.158(-5.408,-4.908) |
| Malawi | 20829.069 ( 14448.684 , 28634.471 ) | 47016.293 ( 36670.199 , 58653.093 ) | 2305.155 ( 1443.926 , 3554.635 ) | 13.437 ( 7.984 , 19.082 ) | -3.331(-3.558,-3.104) | -1.440(-1.528,-1.351) | -5.323(-5.625,-5.021) | -6.588(-6.943,-6.232) |
| Malaysia | 3220.61 ( 2422.005 , 4150.748 ) | 16963.966 ( 13711.322 , 22552.334 ) | 447.853 ( 286.482 , 689.134 ) | 0.218 ( 0.139 , 0.341 ) | -3.236(-3.427,-3.045) | -1.328(-1.411,-1.246) | -2.277(-2.355,-2.198) | -4.232(-4.873,-3.587) |
| Maldives | 5787.97 ( 4084.196 , 8091.318 ) | 25664.504 ( 19628.852 , 37260.095 ) | 638.72 ( 359.486 , 1080.229 ) | 0.855 ( 0.535 , 1.307 ) | -6.494(-6.970,-6.015) | -3.016(-3.235,-2.797) | -5.970(-6.507,-5.429) | -10.306(-11.053,-9.553) |
| Mali | 38934.31 ( 28821.393 , 50149.872 ) | 64482.336 ( 56409.861 , 72631.873 ) | 6420.613 ( 4294.22 , 8991.214 ) | 48.303 ( 29.914 , 71.182 ) | -2.371(-2.430,-2.312) | -0.887(-0.963,-0.812) | -2.258(-2.597,-1.918) | -2.934(-3.330,-2.537) |
| Malta | 1826.859 ( 1273.872 , 2578.245 ) | 6142.59 ( 4610.626 , 8295.217 ) | 92.53 ( 44.98 , 174.597 ) | 0.005 ( 0.004 , 0.007 ) | -3.222(-3.446,-2.999) | -2.113(-2.230,-1.996) | -2.205(-2.309,-2.101) | -0.412(-0.776,-0.047) |
| Marshall Islands | 26735.818 ( 18434.196 , 37145.679 ) | 43395.073 ( 34643.207 , 54736.267 ) | 928.831 ( 591.643 , 1409.591 ) | 2.23 ( 1.373 , 3.504 ) | -1.791(-1.861,-1.720) | -0.967(-0.988,-0.946) | -1.046(-1.281,-0.811) | -2.646(-3.283,-2.005) |
| Mauritania | 13852.163 ( 9592.62 , 19983.273 ) | 41405.829 ( 32742.623 , 53369.621 ) | 1270.536 ( 743.973 , 2099.735 ) | 2.056 ( 1.109 , 3.713 ) | -3.600(-3.703,-3.497) | -1.358(-1.391,-1.325) | -4.266(-4.388,-4.144) | -8.892(-9.142,-8.642) |
| Mauritius | 4576.369 ( 3306.928 , 6193.641 ) | 24400.826 ( 17727.515 , 40217.423 ) | 481.87 ( 294.526 , 783.847 ) | 0.568 ( 0.451 , 0.669 ) | -3.851(-3.975,-3.726) | -1.524(-1.625,-1.422) | -2.148(-2.362,-1.934) | -2.521(-3.624,-1.405) |
| Mexico | 8998.622 ( 6052.336 , 13235.301 ) | 16265.404 ( 13477.868 , 20059.412 ) | 358.28 ( 275.281 , 473.165 ) | 1.756 ( 1.321 , 2.311 ) | -3.070(-3.216,-2.923) | -2.115(-2.179,-2.051) | -5.556(-5.720,-5.392) | -7.426(-7.615,-7.236) |
| Micronesia (Federated States of) | 40211.678 ( 29784.378 , 51682.427 ) | 51303.363 ( 41414.133 , 62184.942 ) | 786.233 ( 482.808 , 1238.217 ) | 1.432 ( 0.849 , 2.189 ) | -0.966(-1.026,-0.907) | -0.720(-0.745,-0.695) | -2.109(-2.203,-2.015) | -5.182(-5.336,-5.029) |
| Monaco | 883.957 ( 583.393 , 1240.944 ) | 3452.366 ( 2583.11 , 4950.873 ) | 49.623 ( 21.477 , 95.898 ) | 0.004 ( 0.003 , 0.006 ) | -1.218(-1.387,-1.048) | -1.178(-1.234,-1.122) | -0.942(-0.986,-0.898) | -2.752(-3.476,-2.022) |
| Mongolia | 2377.184 ( 1627.713 , 3445.386 ) | 21559.919 ( 17518.588 , 26213.365 ) | 836.338 ( 474.829 , 1348.298 ) | 0.103 ( 0.054 , 0.179 ) | -4.512(-4.676,-4.348) | -1.599(-1.681,-1.517) | -2.075(-2.193,-1.958) | -8.000(-8.219,-7.781) |
| Montenegro | 6907.595 ( 4547.973 , 10368.69 ) | 17087.915 ( 13341.386 , 22433.744 ) | 236.2 ( 129.249 , 399.662 ) | 0.017 ( 0.01 , 0.026 ) | -2.788(-3.203,-2.371) | -1.666(-1.905,-1.426) | -1.569(-1.776,-1.362) | -5.280(-5.878,-4.677) |
| Morocco | 8169.843 ( 5336.106 , 11832.421 ) | 23760.825 ( 18920.301 , 30333.69 ) | 572.195 ( 327.798 , 923.495 ) | 0.426 ( 0.252 , 0.668 ) | -3.443(-3.773,-3.112) | -1.734(-1.875,-1.592) | -2.539(-2.682,-2.396) | -6.894(-7.206,-6.581) |
| Mozambique | 27223.762 ( 18908.889 , 36864.509 ) | 46650.639 ( 38314.678 , 57312.054 ) | 2189.286 ( 1468.428 , 3168.164 ) | 12.336 ( 7.764 , 18.512 ) | -2.918(-3.076,-2.761) | -1.547(-1.623,-1.472) | -5.610(-5.807,-5.414) | -7.189(-7.389,-6.988) |
| Myanmar | 10245.477 ( 6833.212 , 14601.395 ) | 37314.613 ( 29183.478 , 47520.534 ) | 949.464 ( 538.571 , 1614.081 ) | 1.324 ( 0.837 , 2.064 ) | -5.127(-5.265,-4.989) | -1.631(-1.699,-1.564) | -3.680(-3.801,-3.557) | -8.140(-8.445,-7.833) |
| Namibia | 10264.121 ( 6925.356 , 14660.13 ) | 31474.899 ( 24936.127 , 42806.24 ) | 1688.632 ( 1130.298 , 2465.908 ) | 11.215 ( 7.142 , 17.031 ) | -2.984(-3.173,-2.794) | -1.322(-1.444,-1.199) | -1.995(-2.302,-1.687) | -2.233(-2.774,-1.689) |
| Nauru | 15755.756 ( 10264.98 , 23202.549 ) | 32862.025 ( 25502.675 , 43786.568 ) | 925.508 ( 614.751 , 1390.628 ) | 3.758 ( 2.328 , 5.739 ) | -0.989(-1.537,-0.438) | -0.539(-0.813,-0.263) | -1.237(-1.823,-0.647) | -2.136(-3.100,-1.162) |
| Nepal | 6956.025 ( 4778.151 , 10056.107 ) | 35360.441 ( 26473.839 , 52816.537 ) | 1133.725 ( 733.652 , 1847.914 ) | 3.606 ( 2.2 , 5.504 ) | -5.048(-5.108,-4.988) | -1.313(-1.424,-1.201) | -5.980(-6.327,-5.632) | -9.179(-9.365,-8.992) |
| Netherlands | 1105.538 ( 727.754 , 1572.064 ) | 4797.016 ( 3556.22 , 6374.354 ) | 94.158 ( 45.211 , 166.631 ) | 0.017 ( 0.014 , 0.02 ) | -1.838(-2.055,-1.621) | -1.583(-1.675,-1.491) | -1.452(-1.586,-1.319) | -1.804(-2.247,-1.359) |
| New Zealand | 821.72 ( 603.831 , 1084.98 ) | 7031.674 ( 3300.419 , 17963.002 ) | 71.564 ( 21.426 , 285.376 ) | 0.003 ( 0.002 , 0.004 ) | -1.200(-1.524,-0.875) | -0.938(-1.027,-0.848) | -1.220(-1.330,-1.110) | -2.902(-4.245,-1.540) |
| Nicaragua | 2004.354 ( 1365.391 , 2967.469 ) | 12340.478 ( 9758.339 , 15794.382 ) | 458.082 ( 299.15 , 686.502 ) | 2.09 ( 1.316 , 3.113 ) | -6.283(-6.888,-5.673) | -2.298(-2.498,-2.097) | -4.700(-4.925,-4.474) | -6.676(-7.047,-6.305) |
| Niger | 63339.431 ( 52791.263 , 73236.104 ) | 71506.918 ( 62638.679 , 79610.255 ) | 2274.038 ( 1449.473 , 3416.585 ) | 9.99 ( 6.142 , 14.865 ) | -1.187(-1.239,-1.134) | -0.760(-0.784,-0.736) | -6.076(-6.437,-5.714) | -8.561(-9.094,-8.024) |
| Nigeria | 8042.217 ( 5584.626 , 11244.683 ) | 37144.301 ( 30598.387 , 45105.939 ) | 1598.219 ( 1017.177 , 2412.238 ) | 3.556 ( 2.233 , 5.02 ) | -3.476(-3.829,-3.121) | -0.622(-0.716,-0.528) | -2.453(-2.670,-2.235) | -5.439(-5.800,-5.076) |
| Niue | 8852.282 ( 5760.256 , 13047.352 ) | 24419.52 ( 18802.88 , 33297.177 ) | 1077.861 ( 769.544 , 1493.905 ) | 7.445 ( 5.13 , 10.362 ) | -2.244(-2.370,-2.118) | -1.132(-1.169,-1.096) | -0.839(-1.287,-0.389) | -0.706(-1.644,0.241) |
| North Macedonia | 13860.847 ( 9095.563 , 20207.265 ) | 23953.966 ( 18862.882 , 30684.53 ) | 307.623 ( 174.058 , 522.441 ) | 0.025 ( 0.015 , 0.038 ) | -2.884(-3.021,-2.746) | -2.012(-2.102,-1.922) | -2.318(-2.451,-2.185) | -5.943(-6.636,-5.246) |
| Northern Mariana Islands | 6235.019 ( 4000.398 , 9249.186 ) | 20198.691 ( 15326.98 , 27917.765 ) | 387.478 ( 242.637 , 622.953 ) | 0.721 ( 0.497 , 1.029 ) | -0.449(-0.684,-0.214) | -0.336(-0.467,-0.205) | -0.383(-0.550,-0.216) | -1.253(-1.579,-0.927) |
| Norway | 1007.294 ( 714.63 , 1361.764 ) | 4469.54 ( 3151.873 , 7014.03 ) | 71.607 ( 34.07 , 134.656 ) | 0.008 ( 0.007 , 0.009 ) | -2.349(-2.628,-2.069) | -1.934(-2.075,-1.792) | -1.948(-2.206,-1.690) | 6.099(3.303,8.970) |
| Oman | 3619.956 ( 2717.774 , 4706.768 ) | 20334.077 ( 14908.166 , 33237.713 ) | 413.764 ( 252.92 , 681.312 ) | 0.703 ( 0.469 , 1.026 ) | -4.159(-4.463,-3.855) | -1.802(-1.944,-1.659) | -3.263(-3.723,-2.801) | -5.422(-6.599,-4.230) |
| Pakistan | 6411.116 ( 4663.005 , 8665.752 ) | 42650.633 ( 35400.643 , 51450.876 ) | 2152.354 ( 1413.979 , 3095.456 ) | 4.25 ( 2.862 , 6.092 ) | -4.094(-4.367,-3.819) | -0.785(-0.810,-0.761) | -1.311(-1.435,-1.187) | -2.857(-3.318,-2.394) |
| Palau | 7314.244 ( 4682.983 , 11134.048 ) | 23389.64 ( 17991.595 , 32615.06 ) | 513.221 ( 315.846 , 831.434 ) | 1.014 ( 0.658 , 1.505 ) | -1.348(-1.519,-1.176) | -0.706(-0.776,-0.636) | -1.174(-1.266,-1.083) | -2.547(-2.774,-2.320) |
| Palestine | 5233.932 ( 3507.685 , 7884.832 ) | 16628.08 ( 13217.661 , 22841.071 ) | 309.483 ( 173.605 , 528.086 ) | 0.188 ( 0.127 , 0.27 ) | -4.308(-4.510,-4.106) | -2.311(-2.386,-2.237) | -2.599(-2.692,-2.506) | -4.322(-4.656,-3.986) |
| Panama | 2895.917 ( 1859.686 , 4276.194 ) | 13176.028 ( 9960.421 , 18706.938 ) | 462.15 ( 339.722 , 647.081 ) | 2.951 ( 2.15 , 3.854 ) | -2.902(-3.239,-2.563) | -1.223(-1.360,-1.086) | -3.312(-3.865,-2.755) | -4.244(-5.068,-3.413) |
| Papua New Guinea | 13609.559 ( 9754.158 , 18849.833 ) | 32207.884 ( 24629.728 , 44608.789 ) | 943.435 ( 587.83 , 1577.852 ) | 2.295 ( 1.449 , 3.49 ) | -0.780(-1.030,-0.529) | 0.008(-0.110,0.125) | -0.092(-0.305,0.122) | -1.678(-1.985,-1.370) |
| Paraguay | 7082.409 ( 4434.092 , 10729.437 ) | 18954.279 ( 14734.525 , 25583.141 ) | 565.034 ( 379.244 , 835.903 ) | 2.256 ( 1.485 , 3.161 ) | -3.242(-3.301,-3.182) | -1.695(-1.729,-1.661) | -2.067(-2.351,-1.783) | -3.054(-3.610,-2.494) |
| Peru | 5881.578 ( 3732.442 , 8854.498 ) | 20083.042 ( 15541.007 , 31067.516 ) | 518.77 ( 346.293 , 766.088 ) | 1.868 ( 1.112 , 2.809 ) | -3.656(-3.927,-3.384) | -2.190(-2.314,-2.067) | -5.957(-6.233,-5.681) | -8.585(-9.029,-8.138) |
| Philippines | 12999.107 ( 8826.858 , 18019.617 ) | 24291.903 ( 19834.048 , 29631.156 ) | 730.035 ( 503.215 , 1048.181 ) | 2.166 ( 1.691 , 2.76 ) | -2.016(-2.316,-1.715) | -1.280(-1.413,-1.148) | -2.531(-2.708,-2.354) | -4.894(-5.188,-4.599) |
| Poland | 6074.959 ( 4006.215 , 8854.532 ) | 17346.387 ( 13084.765 , 24486.305 ) | 241.995 ( 133.212 , 422.081 ) | 0.028 ( 0.023 , 0.034 ) | -3.467(-3.522,-3.413) | -2.097(-2.119,-2.075) | -2.537(-2.631,-2.443) | -1.815(-2.608,-1.015) |
| Portugal | 2443.924 ( 1678.675 , 3540.319 ) | 7031.559 ( 5205.384 , 9399.511 ) | 100.333 ( 47.296 , 187.777 ) | 0.024 ( 0.019 , 0.029 ) | -3.004(-3.288,-2.720) | -2.265(-2.440,-2.089) | -2.449(-2.667,-2.230) | -4.465(-5.472,-3.448) |
| Puerto Rico | 1465.569 ( 1060.966 , 2040.302 ) | 13537.324 ( 10124.039 , 21951.444 ) | 295.527 ( 176.485 , 470.548 ) | 0.461 ( 0.376 , 0.567 ) | -3.379(-3.492,-3.266) | -1.528(-1.618,-1.437) | -1.902(-2.182,-1.622) | -3.629(-4.791,-2.453) |
| Qatar | 2379.375 ( 1766.151 , 3097.461 ) | 11941.788 ( 9651.024 , 15728.23 ) | 237.379 ( 135.828 , 397.452 ) | 0.108 ( 0.072 , 0.159 ) | -1.369(-1.602,-1.135) | -1.739(-1.765,-1.713) | -2.772(-2.898,-2.646) | -2.146(-2.894,-1.393) |
| Republic of Korea | 1081.8 ( 791.138 , 1457.08 ) | 3615.408 ( 2743.788 , 5137.849 ) | 35.05 ( 18.253 , 65.866 ) | 0.018 ( 0.013 , 0.024 ) | -3.932(-4.174,-3.690) | -3.162(-3.472,-2.852) | -3.433(-3.845,-3.019) | -5.695(-5.791,-5.599) |
| Republic of Moldova | 1654.174 ( 1186.739 , 2263.434 ) | 12701.713 ( 9984.65 , 17728.768 ) | 254.035 ( 144.975 , 415.656 ) | 0.184 ( 0.134 , 0.25 ) | -2.519(-2.777,-2.260) | -1.649(-1.798,-1.500) | -2.156(-2.307,-2.004) | -4.661(-6.316,-2.976) |
| Romania | 8321.595 ( 5494.195 , 12440.865 ) | 19512.951 ( 15220.559 , 25797.785 ) | 284.158 ( 162.419 , 463.358 ) | 0.187 ( 0.147 , 0.232 ) | -2.887(-3.048,-2.726) | -1.886(-1.973,-1.800) | -2.486(-2.579,-2.392) | -0.892(-1.616,-0.164) |
| Russian Federation | 1202.252 ( 871.644 , 1567.44 ) | 10220.003 ( 7939.406 , 13381.184 ) | 232.555 ( 132.488 , 380.912 ) | 0.061 ( 0.054 , 0.067 ) | -1.566(-1.790,-1.341) | -1.381(-1.582,-1.180) | -2.280(-2.584,-1.975) | -3.883(-4.582,-3.178) |
| Rwanda | 15617.809 ( 10549.428 , 21685.072 ) | 29264.721 ( 23378.419 , 37905.28 ) | 1307.078 ( 910.341 , 1902.476 ) | 9.287 ( 6.111 , 13.212 ) | -3.485(-3.844,-3.124) | -2.102(-2.303,-1.901) | -7.331(-7.993,-6.664) | -8.449(-9.205,-7.686) |
| Saint Kitts and Nevis | 2384.545 ( 1649.622 , 3403.91 ) | 21613.152 ( 16169.883 , 34452.48 ) | 934.635 ( 670.046 , 1321.663 ) | 5.297 ( 3.948 , 7.119 ) | -3.338(-3.468,-3.207) | -1.292(-1.431,-1.153) | -2.543(-3.119,-1.964) | -3.247(-4.178,-2.307) |
| Saint Lucia | 3846.411 ( 2594.625 , 5576.281 ) | 23360.137 ( 17592.486 , 34868.438 ) | 667.702 ( 423.788 , 1039.08 ) | 1.712 ( 1.172 , 2.446 ) | -2.794(-2.959,-2.629) | -0.998(-1.117,-0.879) | -2.209(-2.578,-1.839) | -4.498(-5.303,-3.687) |
| Saint Vincent and the Grenadines | 4596.035 ( 3016.158 , 7017.507 ) | 25344.058 ( 18935.296 , 39315.917 ) | 927.448 ( 645.205 , 1362.871 ) | 4.188 ( 3.051 , 5.68 ) | -3.137(-3.270,-3.004) | -0.903(-1.000,-0.805) | -2.378(-2.819,-1.936) | -4.002(-4.884,-3.112) |
| Samoa | 19835.106 ( 13278.571 , 28568.865 ) | 34139.608 ( 26366.503 , 45264.179 ) | 647.085 ( 401.576 , 1027.089 ) | 1.252 ( 0.701 , 1.926 ) | -1.464(-1.530,-1.398) | -0.845(-0.875,-0.816) | -1.533(-1.641,-1.425) | -3.646(-3.803,-3.489) |
| San Marino | 1022.501 ( 703.24 , 1417.708 ) | 4680.797 ( 3411.022 , 6788.796 ) | 76.321 ( 35.88 , 146.57 ) | 0.005 ( 0.003 , 0.008 ) | -1.567(-1.867,-1.266) | -1.054(-1.266,-0.843) | -1.117(-1.347,-0.887) | -4.680(-4.886,-4.474) |
| Sao Tome and Principe | 14661.102 ( 9719.326 , 21379.787 ) | 41333.877 ( 32155.94 , 55572.867 ) | 1296.254 ( 805.221 , 2117.617 ) | 4.041 ( 2.541 , 5.93 ) | -4.119(-4.348,-3.889) | -1.437(-1.574,-1.300) | -5.495(-6.060,-4.927) | -8.775(-9.333,-8.214) |
| Saudi Arabia | 2330.716 ( 1729.679 , 2968.083 ) | 12373.145 ( 9949.37 , 15795.412 ) | 341.193 ( 195.994 , 558.243 ) | 0.111 ( 0.068 , 0.172 ) | -2.551(-2.863,-2.238) | -1.650(-1.721,-1.579) | -3.714(-3.791,-3.636) | -10.490(-10.669,-10.311) |
| Senegal | 9958.094 ( 6490.833 , 14688.921 ) | 47986.889 ( 39567.774 , 57508.701 ) | 1823.805 ( 964.388 , 2814.399 ) | 2.878 ( 1.567 , 4.452 ) | -5.026(-5.480,-4.570) | -1.298(-1.486,-1.109) | -3.789(-3.978,-3.600) | -7.890(-8.388,-7.389) |
| Serbia | 14538.195 ( 9614.82 , 21092.553 ) | 23905.555 ( 18364.911 , 31679.415 ) | 253.704 ( 138.611 , 430.972 ) | 0.025 ( 0.017 , 0.036 ) | -3.227(-3.485,-2.969) | -2.339(-2.508,-2.169) | -2.480(-2.608,-2.351) | -5.876(-6.528,-5.219) |
| Seychelles | 3203.544 ( 2225.487 , 4537.978 ) | 16868.516 ( 13250.111 , 22562.949 ) | 409.333 ( 254.958 , 633.82 ) | 0.763 ( 0.56 , 1.029 ) | -3.384(-3.714,-3.053) | -1.484(-1.606,-1.362) | -1.287(-1.402,-1.171) | -1.154(-1.533,-0.774) |
| Sierra Leone | 28028.14 ( 19621.262 , 37388.993 ) | 46996.674 ( 37336.927 , 57445.315 ) | 7642.391 ( 4793.303 , 11515.77 ) | 73.968 ( 44.985 , 112.412 ) | -2.713(-2.932,-2.492) | -1.384(-1.517,-1.252) | -0.244(-0.830,0.345) | -0.251(-0.912,0.414) |
| Singapore | 2366.829 ( 1717.656 , 3082.813 ) | 5183.519 ( 3991.783 , 8142.517 ) | 31.893 ( 14.657 , 63.505 ) | 0.003 ( 0.003 , 0.004 ) | -2.842(-3.026,-2.658) | -2.572(-2.758,-2.385) | -3.389(-3.781,-2.995) | -4.859(-5.445,-4.270) |
| Slovakia | 6377.391 ( 4352.545 , 9235.758 ) | 16645.44 ( 13109.599 , 21726.852 ) | 233.049 ( 128.614 , 394.074 ) | 0.048 ( 0.034 , 0.066 ) | -3.015(-3.117,-2.913) | -1.886(-1.941,-1.831) | -2.120(-2.167,-2.074) | -0.877(-1.183,-0.571) |
| Slovenia | 4715.673 ( 3149.386 , 6985.503 ) | 12543.125 ( 9916.95 , 16396.319 ) | 163.871 ( 90.603 , 269.577 ) | 0.03 ( 0.023 , 0.038 ) | -2.543(-2.640,-2.446) | -2.050(-2.139,-1.961) | -2.540(-2.674,-2.405) | -0.686(-1.162,-0.207) |
| Solomon Islands | 38447.009 ( 28163.158 , 50647.008 ) | 49619.409 ( 39874.121 , 60833.455 ) | 964.384 ( 633.96 , 1477.103 ) | 2.439 ( 1.487 , 3.615 ) | -1.088(-1.211,-0.965) | -0.637(-0.716,-0.559) | -1.337(-1.444,-1.230) | -3.894(-4.134,-3.654) |
| Somalia | 85202.388 ( 77260.982 , 91973.803 ) | 86209.909 ( 80105.069 , 91443.963 ) | 3740.978 ( 2486.773 , 5445.09 ) | 27.688 ( 16.317 , 42.082 ) | -0.443(-0.473,-0.413) | -0.251(-0.266,-0.237) | -2.375(-4.905,0.221) | -3.013(-5.806,-0.137) |
| South Africa | 8819.966 ( 5876.085 , 13081.431 ) | 30167.616 ( 25740.253 , 35612.965 ) | 2060.749 ( 1521.447 , 2701.922 ) | 10.453 ( 7.814 , 13.17 ) | -3.769(-3.822,-3.716) | -1.300(-1.327,-1.273) | -1.241(-1.616,-0.865) | -1.828(-2.525,-1.127) |
| South Sudan | 37281.577 ( 27665.569 , 48240.434 ) | 50127.498 ( 40866.855 , 61349.696 ) | 6186.116 ( 4096.237 , 9294.664 ) | 57.173 ( 35.592 , 90.054 ) | -1.612(-1.712,-1.511) | -0.890(-0.953,-0.826) | -3.180(-3.749,-2.607) | -3.551(-4.280,-2.816) |
| Spain | 1512.698 ( 1108.074 , 2013.738 ) | 5170.822 ( 3940.844 , 6964.195 ) | 83.21 ( 39.263 , 157.919 ) | 0.011 ( 0.009 , 0.013 ) | -2.017(-2.241,-1.793) | -1.710(-1.849,-1.571) | -2.056(-2.249,-1.863) | -2.706(-3.021,-2.389) |
| Sri Lanka | 8475.405 ( 6157.157 , 11471.443 ) | 19482.15 ( 16576.864 , 22841.858 ) | 492.391 ( 310.764 , 763.807 ) | 0.175 ( 0.111 , 0.248 ) | -4.106(-4.222,-3.990) | -2.600(-2.670,-2.529) | -2.330(-2.423,-2.238) | -7.365(-7.688,-7.040) |
| Sudan | 12358.553 ( 8741.409 , 17047.985 ) | 32024.104 ( 26715.243 , 39589.92 ) | 1184.227 ( 743.033 , 1779.341 ) | 2.873 ( 1.615 , 4.868 ) | -3.489(-3.844,-3.132) | -1.520(-1.634,-1.407) | -4.547(-4.687,-4.407) | -7.736(-8.182,-7.288) |
| Suriname | 5176.328 ( 3398.803 , 7713.049 ) | 27631.73 ( 21213.533 , 40611.491 ) | 819.649 ( 521.598 , 1281.77 ) | 2.041 ( 1.274 , 3.06 ) | -2.683(-2.731,-2.635) | -0.781(-0.845,-0.717) | -2.725(-2.944,-2.506) | -5.834(-6.205,-5.462) |
| Sweden | 1150.666 ( 822.507 , 1540.388 ) | 4899.615 ( 3547.852 , 6980.84 ) | 79.689 ( 35.8 , 148.519 ) | 0.006 ( 0.005 , 0.007 ) | -2.034(-2.314,-1.754) | -1.484(-1.578,-1.390) | -1.501(-1.656,-1.344) | -0.781(-1.371,-0.187) |
| Switzerland | 734.477 ( 520.865 , 1043.361 ) | 4573.607 ( 3213.584 , 6661.487 ) | 71.819 ( 28.909 , 140.049 ) | 0.017 ( 0.013 , 0.02 ) | -1.694(-1.848,-1.540) | -1.450(-1.555,-1.345) | -1.651(-1.839,-1.463) | -3.497(-3.943,-3.050) |
| Syrian Arab Republic | 8483.839 ( 6220.091 , 11376.142 ) | 22873.063 ( 19109.508 , 28963.277 ) | 644.595 ( 405.202 , 972.64 ) | 0.647 ( 0.42 , 0.931 ) | -2.026(-2.196,-1.855) | -1.282(-1.387,-1.177) | -2.119(-2.441,-1.797) | -5.217(-6.193,-4.230) |
| Taiwan (Province of China) | 1831.274 ( 1367.702 , 2361.247 ) | 6849.086 ( 5517.598 , 8492.559 ) | 124.951 ( 75.224 , 202.681 ) | 0.157 ( 0.128 , 0.19 ) | -3.179(-3.533,-2.824) | -2.171(-2.398,-1.944) | -2.663(-3.069,-2.255) | -5.420(-6.993,-3.822) |
| Tajikistan | 12230.814 ( 8429.463 , 17338.761 ) | 30810.59 ( 25892.177 , 36588.492 ) | 1025.387 ( 601.353 , 1568.24 ) | 0.759 ( 0.364 , 1.403 ) | -1.375(-1.799,-0.950) | -0.810(-0.965,-0.655) | -1.582(-1.817,-1.347) | -5.715(-6.654,-4.766) |
| Thailand | 3984.595 ( 2976.35 , 5244.627 ) | 14047.411 ( 11456.08 , 17917.915 ) | 326.197 ( 206.674 , 504.461 ) | 0.498 ( 0.355 , 0.691 ) | -5.594(-5.834,-5.354) | -2.795(-2.986,-2.604) | -2.104(-2.252,-1.956) | -3.053(-3.646,-2.457) |
| Timor-Leste | 21959.727 ( 16317.372 , 29265.928 ) | 36990.593 ( 30496.462 , 49189.426 ) | 1310.454 ( 940.32 , 1862.757 ) | 7.045 ( 4.262 , 11.771 ) | -3.158(-3.219,-3.097) | -1.660(-1.782,-1.537) | -6.419(-6.870,-5.965) | -8.527(-8.986,-8.066) |
| Togo | 22005.326 ( 14822.225 , 30756.003 ) | 54249.429 ( 44482.179 , 63144.823 ) | 2021.673 ( 1145.942 , 3246.43 ) | 2.906 ( 1.727 , 4.613 ) | -2.720(-2.841,-2.599) | -0.658(-0.740,-0.576) | -2.247(-2.467,-2.027) | -7.261(-7.521,-6.999) |
| Tokelau | 11697.24 ( 7417.576 , 17332.09 ) | 27111.085 ( 20872.805 , 37385.803 ) | 1173.27 ( 802.148 , 1608.553 ) | 8.112 ( 5.236 , 11.493 ) | -2.645(-2.802,-2.487) | -1.390(-1.452,-1.328) | -1.684(-2.327,-1.037) | -3.310(-4.961,-1.630) |
| Tonga | 16618.19 ( 10754.35 , 24057.324 ) | 29346.268 ( 23207.533 , 37433.113 ) | 730.52 ( 492.91 , 1096.745 ) | 2.58 ( 1.692 , 3.844 ) | -1.422(-1.566,-1.278) | -0.847(-0.923,-0.771) | -1.176(-1.292,-1.061) | -2.461(-2.741,-2.181) |
| Trinidad and Tobago | 2796.975 ( 1986.102 , 3967.417 ) | 21943.526 ( 16605.184 , 34139.74 ) | 547.804 ( 334.616 , 877.393 ) | 0.691 ( 0.504 , 0.965 ) | -2.252(-2.372,-2.132) | -1.077(-1.139,-1.015) | -2.575(-2.706,-2.443) | -6.474(-6.796,-6.150) |
| Tunisia | 2847.185 ( 1993.266 , 4003.332 ) | 13531.255 ( 11020.346 , 16781.43 ) | 383.443 ( 219.2 , 634.029 ) | 0.13 ( 0.077 , 0.2 ) | -4.176(-4.337,-4.014) | -1.823(-1.878,-1.767) | -2.265(-2.364,-2.166) | -7.099(-7.406,-6.792) |
| Turkey | 3702.202 ( 2457.7 , 5407.086 ) | 15635.211 ( 12359.207 , 20564.983 ) | 366.599 ( 220.532 , 584.382 ) | 0.532 ( 0.367 , 0.741 ) | -2.013(-2.442,-1.581) | -0.952(-1.035,-0.870) | -1.950(-2.026,-1.874) | -7.563(-7.875,-7.250) |
| Turkmenistan | 4040.149 ( 2804.096 , 5793.69 ) | 24117.45 ( 19472.946 , 31208.249 ) | 776.637 ( 436.923 , 1265.129 ) | 0.247 ( 0.19 , 0.317 ) | -1.996(-2.103,-1.889) | -1.160(-1.217,-1.104) | -3.181(-3.435,-2.927) | -6.906(-7.396,-6.413) |
| Tuvalu | 21540.513 ( 14330.09 , 30989.877 ) | 37136.634 ( 29386.043 , 48044.595 ) | 769.839 ( 487.832 , 1211.45 ) | 1.894 ( 1.208 , 2.87 ) | -4.857(-5.052,-4.661) | -2.589(-2.658,-2.519) | -4.102(-4.300,-3.903) | -6.323(-6.625,-6.020) |
| Uganda | 11086.177 ( 7522.509 , 15718.417 ) | 27029.543 ( 20388.683 , 36072.302 ) | 1560.808 ( 934.182 , 2511.82 ) | 11.358 ( 6.159 , 17.967 ) | -3.783(-3.892,-3.674) | -2.080(-2.221,-1.939) | -4.639(-4.867,-4.411) | -5.304(-5.544,-5.064) |
| Ukraine | 3457.039 ( 2598.75 , 4439.229 ) | 14106.506 ( 11603.499 , 17523.484 ) | 376.418 ( 230.777 , 584.307 ) | 0.09 ( 0.073 , 0.108 ) | -0.312(-0.581,-0.041) | -0.657(-0.740,-0.574) | -1.711(-1.926,-1.495) | -10.808(-12.046,-9.554) |
| United Arab Emirates | 6697.369 ( 5209.77 , 8358.869 ) | 18914.095 ( 15543.887 , 23880.228 ) | 410.661 ( 243.438 , 678.347 ) | 0.099 ( 0.067 , 0.142 ) | -0.495(-0.774,-0.216) | -0.677(-0.794,-0.559) | -1.933(-2.066,-1.800) | -4.156(-4.707,-3.601) |
| United Kingdom | 1322.352 ( 957.152 , 1748.495 ) | 7310.888 ( 5210.436 , 10855.399 ) | 154.458 ( 81.219 , 279.759 ) | 0.008 ( 0.007 , 0.008 ) | -2.360(-2.625,-2.093) | -0.955(-1.069,-0.841) | -0.843(-0.957,-0.729) | -3.828(-4.222,-3.432) |
| United Republic of Tanzania | 11229.348 ( 7646.116 , 15983.109 ) | 38356.601 ( 29189.762 , 53188.239 ) | 1943.192 ( 1235.079 , 3139.116 ) | 12.08 ( 7.623 , 17.863 ) | -2.823(-3.037,-2.608) | -1.287(-1.378,-1.197) | -5.271(-5.460,-5.083) | -6.275(-6.500,-6.049) |
| United States of America | 1093.477 ( 783.632 , 1499.44 ) | 3235.676 ( 2512.202 , 4459.092 ) | 53.696 ( 34.158 , 83.07 ) | 0.067 ( 0.059 , 0.074 ) | -2.064(-2.453,-1.674) | -1.043(-1.136,-0.951) | 0.712(0.436,0.989) | 0.884(-0.046,1.823) |
| United States Virgin Islands | 1498.732 ( 1068.607 , 2118.144 ) | 17660.201 ( 12999.832 , 29612.887 ) | 392.175 ( 221.37 , 627.866 ) | 0.554 ( 0.322 , 0.876 ) | -3.538(-3.713,-3.364) | -0.978(-1.102,-0.853) | -1.889(-2.122,-1.654) | -4.661(-4.990,-4.331) |
| Uruguay | 6965.025 ( 4439.833 , 10734.236 ) | 15149.426 ( 9340.483 , 25471.636 ) | 135.628 ( 69.795 , 255.958 ) | 0.421 ( 0.323 , 0.533 ) | -2.375(-2.585,-2.165) | -1.757(-1.822,-1.692) | -4.411(-4.647,-4.174) | -7.093(-7.769,-6.412) |
| Uzbekistan | 4290.963 ( 3006.501 , 5994.941 ) | 33497.443 ( 25636.881 , 47438.087 ) | 986.947 ( 558.034 , 1658.685 ) | 0.196 ( 0.147 , 0.256 ) | -3.101(-3.177,-3.026) | -0.849(-0.896,-0.802) | -1.662(-1.737,-1.587) | -8.567(-9.290,-7.839) |
| Vanuatu | 37163.505 ( 27029.581 , 48589.564 ) | 47657.597 ( 38920.957 , 57359.686 ) | 974.376 ( 639.79 , 1456.359 ) | 2.726 ( 1.537 , 4.31 ) | -1.035(-1.141,-0.930) | -0.602(-0.664,-0.540) | -0.909(-1.152,-0.664) | -2.641(-3.250,-2.028) |
| Venezuela (Bolivarian Republic of) | 8179.386 ( 5447.238 , 12000.512 ) | 17604.888 ( 13784.302 , 22464.48 ) | 517.166 ( 359.333 , 733.12 ) | 3.013 ( 2.15 , 4.142 ) | -1.320(-1.799,-0.838) | -0.877(-1.123,-0.631) | -3.401(-4.089,-2.708) | -4.593(-5.654,-3.520) |
| Viet Nam | 6705.438 ( 4939.118 , 9188.898 ) | 17186.487 ( 14409.486 , 20507.978 ) | 400.547 ( 246.811 , 631.727 ) | 0.386 ( 0.237 , 0.593 ) | -3.183(-3.520,-2.845) | -1.995(-2.098,-1.893) | -3.870(-4.011,-3.729) | -8.838(-9.236,-8.438) |
| Yemen | 18788.109 ( 13435.146 , 25730.467 ) | 47819.789 ( 43015.838 , 52895.284 ) | 2949.262 ( 2124.367 , 4021.999 ) | 5.865 ( 3.545 , 8.715 ) | -3.372(-3.496,-3.248) | -0.977(-1.029,-0.925) | -2.051(-2.163,-1.938) | -5.524(-5.909,-5.138) |
| Zambia | 14030.275 ( 9660.429 , 20183.831 ) | 46258.992 ( 36554.957 , 56003.628 ) | 2166.285 ( 1233.521 , 3374.052 ) | 9.843 ( 5.731 , 14.833 ) | -3.620(-4.034,-3.204) | -0.996(-1.140,-0.851) | -4.717(-5.078,-4.354) | -6.651(-7.210,-6.089) |
| Zimbabwe | 23280.94 ( 16223.626 , 32160.62 ) | 42805.448 ( 35030.325 , 52830.398 ) | 3691.028 ( 2597.102 , 5218.236 ) | 31.056 ( 20.923 , 43.542 ) | -0.751(-0.999,-0.503) | -0.075(-0.220,0.070) | 1.571(1.200,1.943) | 2.120(1.593,2.651) |

EAPC = estimated annual percentage change; SDI = socio-demographic index; 95% CI = 95% confidence interval.
